# Fast 3D Imaging of Spine, Dendritic, and Neuronal Assemblies in Behaving Animals

**DOI:** 10.1016/j.neuron.2016.10.002

**Published:** 2016-11-23

**Authors:** Gergely Szalay, Linda Judák, Gergely Katona, Katalin Ócsai, Gábor Juhász, Máté Veress, Zoltán Szadai, András Fehér, Tamás Tompa, Balázs Chiovini, Pál Maák, Balázs Rózsa

**Affiliations:** 1 Laboratory of 3D Functional Network and Dendritic Imaging, Institute of Experimental Medicine, Hungarian Academy of Sciences, Budapest 1083, Hungary; 2 MTA-PPKE ITK-NAP B–2p Measurement Technology Group, Faculty of Information Technology, Pázmány Péter Catholic University, Budapest 1083, Hungary; 3 Two-Photon Laboratory, Faculty of Information Technology, Pázmány Péter Catholic University, Budapest 1083, Hungary; 4 Department of Atomic Physics, Budapest University of Technology and Economics, Budapest 1111, Hungary

## Abstract

Understanding neural computation requires methods such as 3D acousto-optical (AO) scanning that can simultaneously read out neural activity on both the somatic and dendritic scales. AO point scanning can increase measurement speed and signal-to-noise ratio (SNR) by several orders of magnitude, but high optical resolution requires long point-to-point switching time, which limits imaging capability. Here we present a novel technology, 3D DRIFT AO scanning, which can extend each scanning point to small 3D lines, surfaces, or volume elements for flexible and fast imaging of complex structures simultaneously in multiple locations. Our method was demonstrated by fast 3D recording of over 150 dendritic spines with 3D lines, over 100 somata with squares and cubes, or multiple spiny dendritic segments with surface and volume elements, including in behaving animals. Finally, a 4-fold improvement in total excitation efficiency resulted in about 500 × 500 × 650 μm scanning volume with genetically encoded calcium indicators (GECIs).

## Highlights

• 3D DRIFT AO scanning can extend each point to 3D lines, surfaces, or volume elements
• Retained spatial information is used for post hoc movement correction of signals
• Chessboard; snake; multi-cube; multi-3D line; and multi-layer, multi-frame scanning
• Improved excitation efficiency for large 3D scanning volume with GECIs

## Teaser

3D DRIFT acousto-optical microscopy designed by Szalay et al. allows confined two-photon scanning of multiple 3D regions of interest. Activity of hundreds of neurons or multiple dendritic processes can be measured simultaneously in large cortical volumes of behaving animals.

## Introduction

Neuronal diversity, layer specificity of information processing, area-wise specialization of neural mechanisms, internally generated patterns, and dynamic network properties all show that understanding neural computation requires fast readout of information flow and processing. Moreover, this fast recording is required not only from a single plane or point, but also at the level of large neuronal populations situated in large 3D volumes (Scanziani and Häusser, 2009, Katona et al., 2012, Khodagholy et al., 2015). In addition, coding and computation within neuronal networks are generated not only by the somatic integration domains, but also by highly non-linear dendritic integration centers that, in most cases, remain hidden from somatic recordings (Poirazi et al., 2003, Polsky et al., 2004, Losonczy and Magee, 2006, Johnston and Narayanan, 2008). Therefore, we need to simultaneously read out neural activity at both the population and single-cell levels. Moreover, it has been shown that neuronal signaling could be completely different in awake and behaving animals (Niell and Stryker, 2010, Barth and Poulet, 2012, Fu et al., 2014). We therefore need novel methods that can simultaneously record activity patterns of neuronal, dendritic, spinal, and axonal assemblies with high spatial and temporal resolution in large scanning volumes in the brain of behaving animals.

Several new optical methods have recently been developed for the fast readout of neuronal network activity in 3D. For example, it is possible to record 3D structures using spatial light modulators (Nikolenko et al., 2008), liquid lenses (Grewe et al., 2011), acousto-optical (AO) deflectors (Duemani Reddy et al., 2008, Katona et al., 2012, Fernández-Alfonso et al., 2014), temporal multiplexing (Cheng et al., 2011), axicon or planar illumination-based imaging (Holekamp et al., 2008), fast z-scanning based on an axially moving mirror (Botcherby et al., 2012), piezo actuators (Göbel et al., 2007, Katona et al., 2011), simultaneous multi-view light sheet microscopy (Tomer et al., 2012), two-photon light sheet microscopy (Wolf et al., 2015), optical fibers addressed by AO deflectors (Rózsa et al., 2007), light field microscopy (Prevedel et al., 2014), phase-locked ultrasound lens (Kong et al., 2015), or phase mask combined with holographic illumination (Quirin et al., 2014). Only a handful of these 3D technologies can provide single-point multi-photon excitation that allows whole-field detection of the scattered fluorescence photons required for deep-brain imaging (Helmchen and Denk, 2005). Among the available 3D scanning solutions for multi-photon microscopy, 3D AO scanning is capable of performing 3D random-access point scanning (Katona et al., 2012, Cotton et al., 2013, Fernández-Alfonso et al., 2014) to increase the measurement speed and signal collection efficiency by several orders of magnitude in comparison to classical raster scanning (Supplemental Note, available online). This is because the pre-selected regions of interest (ROIs) can be precisely and rapidly targeted without wasting measurement time on unnecessary background volumes. More quantitatively, if we compare the relative gain in measurement speed (*v*_*gain*_) and signal-to-noise ratio (*SNR*_*gain*_) for 3D AO scanning relative to traditional raster scanning of the same sample volume, we can say that the $v_{gain}*{\left(SNR_{gain}\right)}^{2}$ is equivalent to the ratio of the total image volume to the volume covered by the pre-selected scanning points (Equation S82). This ratio can be very large, up to about 10^6^ per ROI (Supplemental Note; Equation S85), which makes 3D AO scanning suitable for precise multisite activity measurements, especially when ROIs are sparsely dispersed in the 3D volume.

In spite of the evident benefits of 3D random-access AO microscopy, the method can face a major technical limitation: fluorescence data are lost or contaminated with large amplitude movement artifacts during in vivo recordings. This is because the actual location of the recorded ROIs is continuously changing during in vivo measurements due to tissue movement caused by heartbeats, blood flow in nearby vessels, respiration, and physical motion (Greenberg and Kerr, 2009). This results in fluorescence artifacts because of the spatial inhomogeneity in the baseline fluorescence signal and in relative fluorescence changes. In addition, the amplitudes of motion-induced transients can even be larger than the ones induced by one or a few action potentials (APs) detected by genetically encoded calcium indicators (GECIs) (Chen et al., 2013). Therefore, it is difficult to separate post hoc the genuine fluorescence changes associated with neural activity from the artifacts caused by brain movement.

Here we describe a novel method, 3D DRIFT AO microscopy, in which, instead of keeping the same scanning position, we can drift the excitation spot quickly in any direction in 3D space while continuously recording fluorescence data with no limitation on sampling rate (sampling rate is limited during drifts only by the bandwidth of the photomultipliers [PMTs]). In this way, we can extend the pre-selected individual scanning points to small 3D lines, surfaces, or volume elements to cover not only the pre-selected ROIs but also the neighboring background areas or volume elements while utilizing an increased data sampling rate. We developed six new scanning methods based on 3D DRIFT AO scanning and demonstrated that these techniques can decrease the amplitude of motion artifacts by more than one order of magnitude and, therefore, enable the fast functional measurement of neuronal networks at the level of tiny neuronal processes, such as spiny dendritic segments, even in head-restrained, behaving animals in a z-scanning range of more than 650 μm in 3D even under conditions when large-amplitude motion artifacts are generated by physical movement.

## Results

In the first section, we describe 3D DRIFT AO microscopy. In the second section, we show how to increase the scanning volume at longer wavelengths to image GECIs. Then we demonstrate step by step the six new scanning methods (chessboard scanning; multi-layer, multi-frame scanning; ribbon scanning; snake scanning; multi-cube scanning; and multi-3D line scanning) and a quantification of the improvement in SNR.

### Fast 3D DRIFT AO Microscopy

The robust performance of 3D point-by-point scanning performed with AO microscopes has been demonstrated in earlier works in slice preparations and in anesthetized animals (Katona et al., 2012, Cotton et al., 2013, Chiovini et al., 2014, Fernández-Alfonso et al., 2014). In these studies, 3D scanning was achieved by using two groups of x and y deflectors. During focusing, the second x (and y) deflector was supplemented with the same, but counter-propagating, acoustic wave with a linearly changing (chirped) frequency to fully compensate for the lateral drift of the focal spot. In this way, the point scanning method yields high pointing stability but requires relatively long switching times because it is necessary to fill the large AO deflector apertures each time when addressing a new point in 3D.

Our alternative continuous trajectory scanning method (Katona et al., 2012) allows fast lateral scanning in 2D; 3D trajectory scans, however, still need to be interrupted by time-consuming jumps when moving along the z axis. In other words, scanning along the z axis still suffers from the same limitation as during point-by-point scanning. Our aim was to generalize our previous methods by deriving a one-to-one relationship between the focal spot coordinates and speed, and the chirp parameters of the four AO deflectors to allow fast scanning drifts with the focal spot not only in the horizontal plane but also along any straight line in the 3D space, starting at any point in the scanning volume (3D DRIFT AO scanning). To realize this aim, we had to use non-linear chirps with parabolic frequency profiles. The partial drift compensation realized with these parabolic frequency profiles allows the directed and continuous movement of the focal spot in arbitrary directions and with arbitrary velocities determined by the temporal shape of the chirped acoustic signals. During these fast 3D drifts of the focal spot, the fluorescence collection is uninterrupted, lifting the pixel dwell time limitation of the previously used point scanning. In the Experimental Procedures, we briefly describe how to generate 3D drifts with AO scanning, while the expressions are detailed in the Supplemental Experimental Procedures (Equations S1–S70). In Table S1, we summarize the calculation of the ten parameters within which non-linear chirps need to be generated in the four AO deflectors to move a focal spot from a given starting point in any arbitrary direction and with any desired speed during 3D DRIFT AO scanning (Movie S1).

### Increased Scanning Volume for GECI Imaging

The scanning volume of our previous 3D AO microscope was 700 × 700 × 1,400 μm in transparent samples and was limited to only 400 × 400 × 500 μm during in vivo measurements with synthetic dyes due to the low transmitted laser intensity (about 10%; Katona et al., 2012). However, these measurements were performed at around 800 nm, where Ti:S lasers have an intensity maximum and AO deflectors work with high efficiency. In contrast, at the longer wavelengths required for GCaMP6 imaging (>880 nm) the laser intensity and the total transmission of the scan head dropped overall by about 3.5-fold, and, therefore, the scanning volume was reduced to about 150 × 150 × 200 μm. To increase the total transmission efficiency, we have improved both the 3D scan head and the optical pathway (Figure S1; see details in Supplemental Experimental Procedures). We installed AO deflectors developed for longer, 850–950 nm wavelengths (Figures S2A–S2C); increased the center frequency to extend the AO bandwidth; optimized AO deflector angles by carefully modeling the wavelength sensitivity of AO cell orientation (Mihajlik et al., 2014); added a motorized four-prism compressor for dispersion compensation (Figure S1B); and changed many further opto-mechanical elements (Figure S1A). Finally, we optimized the electric circuitry, coupling the driver signal to the piezo transducer, to increase the AO wave generation efficiency and bandwidth, and developed a novel board for AO signal synthesis (Figure S3). Thanks to these improvements, we were able to achieve about a 4-fold higher transmission in the range of 850–950 nm (Figure S2C). The broad field of view, the extended z-scanning range, and the large 3D scanning volume with preserved somatic resolution recorded at 800–810 nm in transparent samples in our earlier work (Katona et al., 2012) were also well preserved at longer wavelengths (650 × 650 × 1,100 μm at 880 nm; Figures S4 and S2D–S2H). The spatial resolution decreased as a function of distance from the center with a similar tendency as earlier (see Figure 1 in Katona et al., 2012) but remained smaller than the typical diameter of neuronal somata in the entire scanning volume (Figure S4). The maximal scanning volume was about 500 × 500 × 650 μm with the GCaMP6f sensor during in vivo measurements (Movie S2). This volume is more than two orders of magnitude larger than those previously reported with GECIs (Fernández-Alfonso et al., 2014). Importantly, in these measurements we used a high numerical aperture objective (1.0), which provided a good spatial resolution (about 400 nm in the center; Figure S4G). Obviously, with low-magnification objectives we can extend the scanning volume, but the lower numerical aperture of the currently available low-magnification objectives decreases spatial resolution (Katona et al., 2012).

### Extending Scanning Points to 3D Lines, Surfaces, and Volume Elements to Record in the Moving Brain

**Figure 1:**
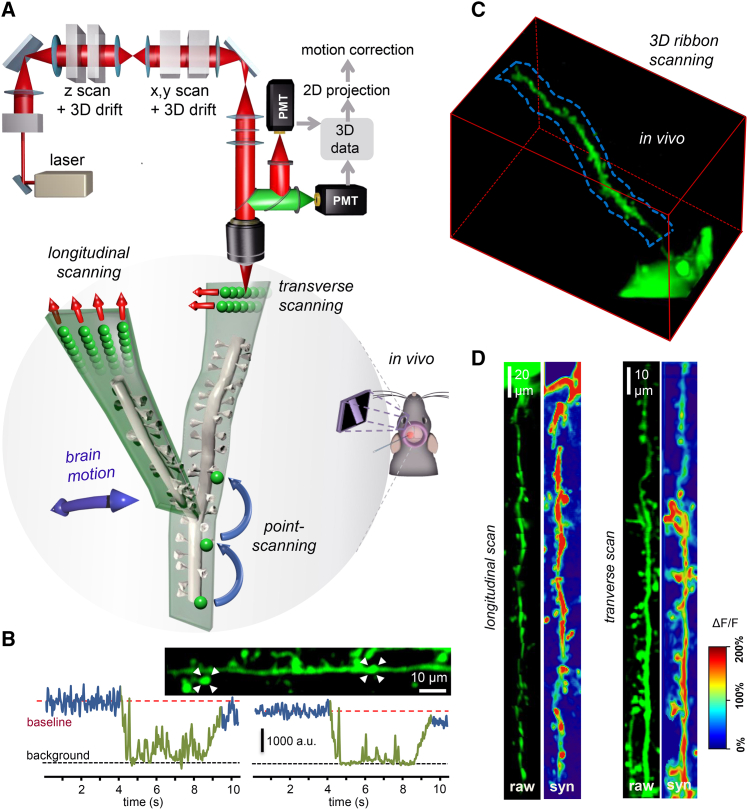
In Vivo Imaging of Spiny Dendritic Segments with 3D DRIFT AO Microscopy in Behaving Animals (A) Schematic of the in vivo measurements performed with 3D DRIFT AO microscopy. In contrast to traditional point-by-point scanning, here the z-scanning and x, y scanning units of the microscope can drift the focal spot in 3D in an arbitrary direction and with an arbitrary speed. Therefore, we can extend individual scanning points to small surface or even volume elements by using, for example, longitudinal or transverse scanning. These surface and volume elements can be set parallel to the average direction of brain movement to preserve fluorescence information for motion correction. (B) Exemplified transients were recorded using 3D random-access point scanning during motion (green) and rest (blue) from ROIs, indicated with white triangles in the inset. Note that fluorescence information can be completely lost in a running period, indicating that single points are not sufficient to monitor activity in behaving animals. (C) Three-dimensional image of a dendritic segment of a selected GCaMP6f-labeled neuron. Cre-dependent GCaMP6f-expressing AAV vectors were used to induce sparse labeling. A 3D ribbon (blue dashed lines) was selected for fast 3D DRIFT AO scanning (Movie S4). Red cube, 140 × 70 × 80 μm^3^. (D) Left, fast 3D ribbon scanning was performed at 139.3 and 70.1 Hz along the blue ribbon indicated in (C) using the longitudinal scanning mode. Raw fluorescence data (raw) were measured along the selected 3D ribbon and were projected into 2D along the longitudinal and transversal axes of the ribbon following elimination of motion artifacts. Average Ca^2+^ responses along the ribbon during spontaneous activity (syn.) are color coded. Movie S4 shows the same dendritic ROI during imaging with 3D ribbon scanning. Right, a similar measurement but with transverse scanning mode. See also Movies S1, S2, S3, S4, and S5.

During 3D DRIFT AO scanning, we can not only scan individual points, but also scan at high speed (up to 10 mm/ms) along any segment of any 3D lines situated in any location in the entire scanning volume with minimal loss of resolution at high speeds (Figure S5). In this way, fluorescence information can be continuously collected when scanning the entire 3D line in the same short period of time (≈20 μs) as required for single-point scanning in the point-by-point scanning mode. The data acquisition rate is limited only by the maximal sampling rate of the PMT units. Therefore, we can generate folded surface (or volume) elements, for example, from transversal or longitudinal 3D lines (Figure 1A); fit them to long, tortuous dendrite segments and branch points in an orientation that minimizes fluorescence loss during brain motion (3D ribbon scanning; Figure 1A); and image them at high speed (Equations S72 and S73). To achieve 3D ribbon scanning, the first step is to select guiding points along a dendritic segment (or along any cellular structure) based on the z stack taken at the beginning of the experiment (Supplemental Experimental Procedures; Movie S3). The second step is to fit a 3D trajectory to these guiding points using piecewise cubic Hermite interpolation. We can use two main strategies to form ribbons along the selected 3D trajectory: we can generate drifts either parallel to the trajectory (longitudinal drifts) or orthogonal to the trajectory (transverse drifts) (Figure 1). In both cases, we chose to maximize how parallel these surface elements lie to the plane of brain motion or to the nominal focal plane of the objective. The basic idea behind the latter is that the point spread function (PSF) is elongated along the z axis: fluorescence measurements are therefore less sensitive to motion along the z axis. Therefore, we can also follow this second strategy and generate multiple x-y frames for neuronal network and neuropil measurements (see below). In the following sections, we demonstrate the implementation and efficiency of the different scanning strategies that can be generated by 3D DRIFT AO scanning.

### 3D Ribbon Scanning to Compensate In Vivo Motion Artifacts

To demonstrate 3D ribbon scanning, we labeled a small number of pyramidal neurons in the V1 region of the visual cortex with a Ca^2+^ sensor, GCaMP6f, using an AAV vector for delivery (Supplemental Experimental Procedures). Then, according to the z stack taken in advance, we selected guiding points and fitted the 3D trajectory, which covered a spiny dendritic segment of a labeled pyramidal cell (Figure 1C). We used transversal drifts to scan along the selected 3D ribbons to measure the selected 140 μm dendritic segment and spines at 70.1 Hz (Figure 1D). Using longitudinal drifts allowed a much faster measurement (between 139.3 and 417.9 Hz) of the same dendritic segment because fewer (but longer) 3D lines were required to cover the same ROI. In the next step, 3D recorded data were projected into 2D as a function of perpendicular and transverse distances along the surface of the ribbon. Note that in this way, the dendritic segment was straightened to a frame (Figure 1D) to record its activity in 2D movies (Movies S4 and S5). This projection also allowed the use of an adapted version of previous methods developed for motion artifact elimination in 2D scanning (Greenberg and Kerr, 2009, Kaifosh et al., 2014) (Movie S4). The need to extend single scanning points to surface or volume elements in order to preserve the surrounding fluorescence information for motion artifact elimination is also indicated by the fact that fluorescence information could be completely lost during motion in behaving animals when using the point scanning method (Figure 1B).

**Figure 2:**
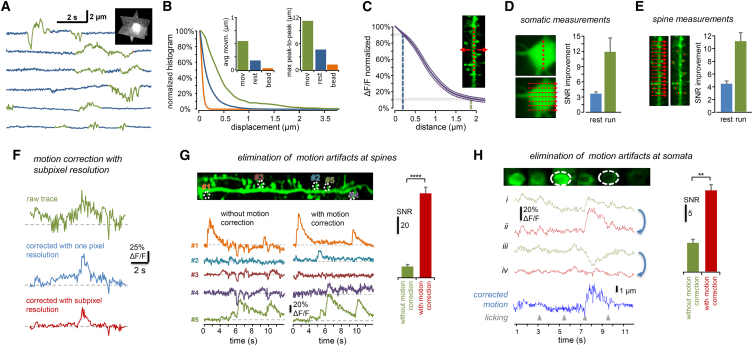
Quantitative Analysis of the Motion Artifact Elimination Capability of 3D DRIFT AO Scanning (A) Brain motion was recorded at 160 Hz by imaging a bright, compact fluorescent object with three orthogonal x-y, x-z, and y-z planes (fast 3D motion-detection method; Figure S6). Exemplified transient of brain displacement projected on the x axis from a 60 s measurement period when the mouse was running (green) or resting (blue) in a linear maze. (B) Displacement data were separated into two intervals according to the recorded locomotion information (running in green and resting in blue), and a normalized amplitude histogram of brain motion was calculated for the two periods. Inset shows average and average peak-to-peak displacements in the resting and running periods. Orange bars are control fluorescent data with fixed beads. (C) Normalized change in relative fluorescence amplitude as a function of distance from the center of GCaMP6f-labeled dendritic segments (*ΔF/F(x)*, mean ± SEM, n = 3). Blue and green dashed lines indicate average maximum peak-to-peak displacement values calculated for the resting and running periods, respectively. Note the >80% drop in ΔF/F amplitude for the average displacement value during running. Inset, dendritic segment example. ΔF/F was averaged along a dashed red line and then the line was shifted and averaging was repeated to calculate ΔF/F(x). (D) Left, image of a soma of a GCaMP6f-labeled neuron. Red points and dashed arrows indicate scanning points and scanning lines, respectively. Right, normalized increase in SNR calculated for resting (blue) and running (green) periods in awake animals when scanning points were extended to scanning lines in somatic recordings, as shown on the left. Calculations are detailed in Figure S8. (E) Similar calculations as in (D), but for dendritic recordings. SNR of point-by-point scanning of dendritic spines was compared to 3D ribbon scanning during resting (blue) and running (green) periods. Note the more than 10-fold improvement when using 3D ribbon scanning. (F) Left, exemplified individual Ca^2+^ transient from a single dendritic spine derived from the movie that was recorded with 3D ribbon scanning along a 49.2 μm spiny dendritic segment in a behaving mouse (green). When Ca^2+^ transients were derived following motion-artifact correction performed at pixel (blue) and subpixel resolution (red), the motion-induced artifacts were eliminated and SNR was improved. (G) Further example. Top, single frame from the movie recorded with 3D ribbon scanning from an awake mouse. Bottom, exemplified Ca^2+^ transients derived from the recorded movie frames from the color-coded regions. Note the improved SNR of the transients when they were derived following motion-artifact correction at subpixel resolution. Right, SNR of spine Ca^2+^ transients with and without motion correction at subpixel resolution (100 transients, n = 5/5 spines/mouse). (H) Left, simultaneous 3D imaging of VIP neuron somata during a classical behavior experiment where conditioned stimulus (water reward) and unconditioned stimulus (air puff, not shown) were given for two different sounds. Exemplified somatic transients are shown with (red) and without motion correction at subpixel resolution (green). Blue trace shows motion amplitude. Note that motion-induced and neuronal Ca^2+^ transients overlap. Moreover, transients could have negative amplitude without motion correction. Right, SNR of the transients with (red) and without (green) motion correction (mean ± SEM, n = 3). See also Figure S8. Bars are mean ± SEM.

In order to quantify motion-induced errors and the efficiency of motion artifact correction during ribbon scanning, we first measured brain movement by rapidly scanning a bright, compact fluorescent object that was surrounded by a darker background region. To do this, we centered three orthogonal squares (in the x-y, x-z, and y-z planes) on the fluorescent object, recorded fluorescence, and calculated displacement from the x, y, and z projections (defined as “fast 3D motion-detection” method) while the mouse was running in a linear virtual track (Figures 2A and S10; Supplemental Experimental Procedures). We separated resting and moving periods according to the simultaneously recorded locomotion information (Figures 2A and 2B). Brain motion can induce fluorescence artifacts because there is a spatial inhomogeneity in baseline fluorescence and also in the relative fluorescence signals (Figure 2C). The amplitude of motion-generated fluorescence transients can be calculated by substituting the average peak-to-peak motion error into the histogram of the relative fluorescence change (Figure 2C). The average ΔF/F(x) histogram was relatively sharp for dendrites (average of 150 cross-sections from n = 3, 100-μm-long dendritic segments; Figure 2C); therefore, the average motion amplitude during running corresponds to a relatively large (80.1% ± 3.1%) drop in the fluorescence amplitude, which is about 28.6- ± 7.74-fold higher than the average amplitude of a single AP-induced Ca^2+^ response (Figure S7) (Chen et al., 2013). These data indicate the need for motion artifact compensation.

Next, we analyzed the efficiency of our methods for motion correction (Supplemental Experimental Procedures) during in vivo measurements. As before, we labeled neurons and their processes with a GCaMP6f sensor, used 3D ribbon scanning, and projected the recorded fluorescence data to movie frames. We got the best results when each frame of the video recorded along 3D ribbons was corrected by shifting the frames at subpixel resolution to maximize the fluorescence cross-correlation between the successive frames (Figure 2F). Ribbon scanning and the successive frameshifts at subpixel resolution in running animals increased SNR by 7.56- ± 3.14-fold (p > 0.000015, n = 10) when compared to 3D random-access point scanning (Figure 2G).

Next, we separately investigated the effect of post hoc frameshifts on the SNR following ribbon scanning. For a precise analytical analysis, we added the same 1, 2, 5, and 10 AP-induced average transients to the images of a soma (Figure S8A). Then, we generated a series of frames by shifting each frame with the amplitude of brain motion recorded in advance (similarly to Figure 2A). Finally, we recalculated Ca^2+^ transients from the frame series with and without using the motion-correction algorithm, using ROIs of the same size to compare the SNR of point-by-point scanning and the motion-corrected 3D ribbon scanning on the same area (Figure S8B). Low-amplitude spine Ca^2+^ transients were barely visible when transients were derived from the raw video. Our data indicate that 3D ribbon scanning, which, in contrast to point-by-point scanning, allows motion correction, can largely improve the SNR in the case of small, one to five AP-associated signals recorded most frequently during in vivo measurements (11.72- ± 2.59-fold, p < 0.025, n = 4 somata, n = 44 repeats for one to ten APs; Figures 2D and S8C). We repeated the entire process on dendritic spines (Figures S8E–S8G). The method also significantly improved the SNR of single-spine Ca^2+^ responses (11.64- ± 1.25-fold, n = 4 spines, n = 44 repeats; Figures 2E and S8G). The average difference between the original motion trajectory and the movement artifact correction, which indicates the error of the method more directly, was 0.139 ± 0.106 μm for somatic and 0.118 ± 0.094 μm for dendritic measurements (Figures S8D and S8H). There was only a small increase in the error (to 0.154 ± 0.120 μm) when the simulated videos were downsampled from 160 to 40 Hz frame rate (Figure S8D). The sum of the FWHM (full width at half maximum) of the PSF and the average diameter of dendrites or spines was much larger (PSF + dendrite, 1.11 ± 0.17 μm; PSF + spine head, 1.31 ± 0.20 μm; spine and dendrite diameters were measured from z stacks shown in Figure 6 and were in good agreement with Tsay and Yuste, 2002) than the average residual error, indicating that the special precision of motion correction is appropriate for dendritic and spine measurements.

Finally, we quantified the efficiency of our method in a “classical” behavioral experimental protocol. We simultaneously recorded multiple somata of vasoactive intestinal polypeptide (VIP)-expressing interneurons during conditioned and unconditioned stimulation (Supplemental Experimental Procedures). Reward induced large responses in GCaMP6f-labeled neurons whose Ca^2+^ signals temporally overlapped with the behavior-induced motion, and, therefore, Ca^2+^ transients were associated with large motion artifacts, and even transients with negative amplitude could be generated. However, our method effectively restored Ca^2+^ transients and improved SNR in these experiments (Figure 2H).

### Recording of Spiny Dendritic Segments with Multiple 3D Ribbon Scanning

**Figure 3:**
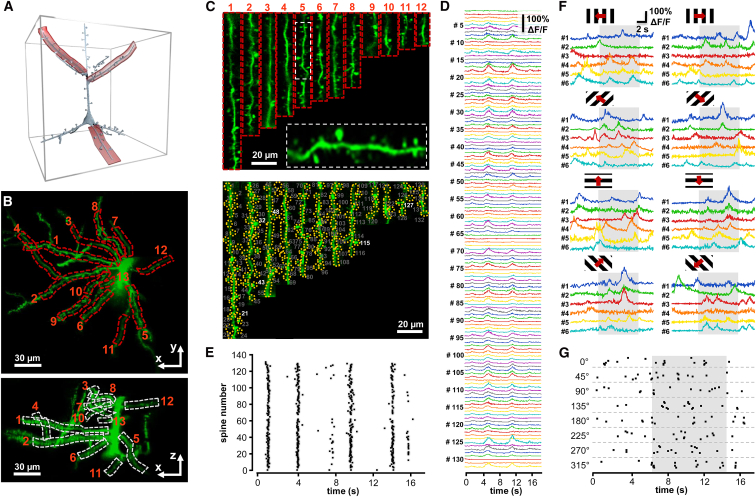
Imaging of Multiple Spiny Dendritic Segments with 3D Ribbon Scanning in Behaving Animals (A) Schematic of the measurement. (B) Maximal intensity projection in the x-y and x-z planes of a GCaMP6f-labeled layer II/III pyramidal neuron. Numbered frames indicate the twelve 3D ribbons used to simultaneously record twelve spiny dendritic segments using 3D ribbon scanning. (C) Top, fluorescence was recorded simultaneously along the twelve dendritic regions shown in (B) (Movie S6). Data were projected into a 2D image as a function of the distance along the longitudinal and transverse directions of each ribbon, and then all images were ordered next to each other. This transformation allowed motion artifact elimination and the simultaneous visualization of the activity of the twelve selected dendritic regions as a 2D movie (Movie S7). The image is a single frame from the movie recorded at 18.4 Hz. Inset, enlarged view shows the preserved resolution. Bottom, 132 ROIs, dendritic segments, and spines selected from the video. (D) Transients derived from the 132 numbered regions. (E) Raster plot of activity pattern of the dendritic spines indicated in (C). (F) Ca^2+^ transients from the six exemplified dendritic spines indicated with white numbers in (C). Transients were induced by moving grating stimulation. Note the variability in timing. (G) Raster plot of the activity pattern of the six dendritic spines from (F). Time of moving grating stimulation in eight different directions is indicated with a gray bar. See also Movies S6 and S7.

Although previous studies have demonstrated the simultaneous recording of multiple dendritic segments under in vitro conditions (Chiovini et al., 2014, Fernández-Alfonso et al., 2014), in vivo recording over large z-scanning ranges has remained an unsolved problem because the brain movement has inhibited the 3D measurement of these fine structures. Therefore, we implemented 3D ribbon scanning to simultaneously record the activity of multiple dendritic segments (Figure 3A). As in the 3D measurement of single dendritic segments, we took a z stack in advance, selected guiding points in 3D along multiple dendritic segments, and fitted 3D trajectories and, finally, 3D ribbons to each of the selected dendritic segments (Figure 3B). As above, the surface of the ribbons was set to be parallel to the average motion vector of the brain to minimize the effect of motion artifacts. We selected twelve dendritic segments from a GCaMP6f-labeled V1 pyramidal neuron for fast 3D ribbon scanning (Figure 3B). In the next step, 3D data recorded along each ribbon were 2D projected as a function of distance perpendicular to the trajectory and along the trajectory of the given ribbon. Then, these 2D projections of the dendritic segments were placed next to each other (Figure 3C). Note that in this way, all the dendritic segments are straightened and visualized in parallel. Hence, we are able to transform and visualize 3D functional data in real time as a standard video movie (raw measurement data; Movie S6). The 2D projection used here allows fast motion artifact elimination and simplifies data storage, data visualization, and manual ROI selection (Figure 3C).

Since each ribbon can be oriented differently in 3D space, the local coordinate system of measurements varies as a function of distance along a given ribbon, and also between ribbons covering different dendritic segments. Therefore, brain motion generates artifacts with different relative directions in each ribbon, so the 2D movement correction methods used previously (Kaifosh et al., 2014) cannot be directly used for the flattened 2D movie generated from ribbons. To solve this issue, we divided the recordings of each dendritic region into short segments (Figure S9). Then, the displacement of each 3D ribbon segment was calculated by cross-correlation, using the brightest image as a reference (Figure S9B). Knowing the original 3D orientation of each segment, the 3D displacement vector for each ribbon segment could be calculated (Figure S9C). Then, we calculated the median of these displacement vectors to estimate the net displacement of the recorded dendritic tree (Figure S9D). Next, we projected back the net displacement vector to each ribbon segment to calculate the required backshift for each image of each ribbon segment for motion elimination (Figure S9E; Movie S7). Finally, we repeated the algorithm separately in each and every segment to let the algorithm correct for local inhomogeneity in displacement (Movie S7). This allowed, for example, the depth-, vasculature-, and distance-dependent inhomogeneities in displacement to be eliminated. Following this 3D-to-2D transformation and motion artifact elimination, we were able to apply previously developed 2D methods to our 3D Ca^2+^ data to calculate regular Ca^2+^ transients from, for example, over 130 spines and dendritic regions (Figures 3C and 3D). Using our methods, we detected spontaneous and visual stimulation-induced activities (Figures 3D and 3F). Finally, we generated two raster plots from spine assembly patterns to demonstrate that both synchronous and asynchronous activities of dendritic spine assemblies can be recorded in behaving animals (Figures 3E and 3G).

### Multi-Layer, Multi-Square Imaging of Neuronal Networks: Chessboard Scanning

**Figure 4:**
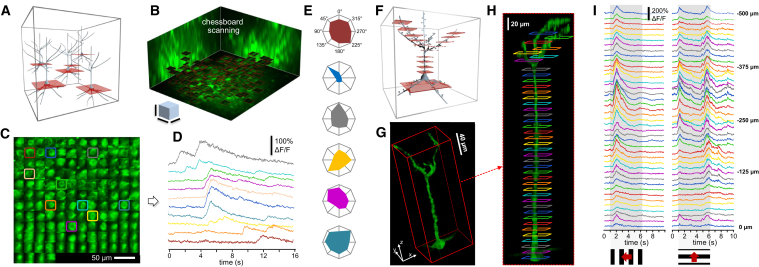
Chessboard Scanning of Neuronal Networks and Multi-Layer, Multi-Frame Imaging of Dendritic Activity of a Layer V Pyramidal Neuron in Behaving Animals (A) Schematic of chessboard scanning. (B) Neurons from a mouse V1 region were labeled with GCaMP6f sensor. Neuronal somata and surrounding background areas (small horizontal frames) were selected according to a z stack taken at the beginning of the measurements (Movie S8). Scale bars, 50 μm. (C) Selected frames are “transformed” into a 2D “chessboard,” where the “squares” correspond to single somata. Therefore, the activity can be recorded as a 2D movie. The image is a single frame from the video recording of 136 somata during visual stimulation (Movie S9). (D) Representative somatic Ca^2+^ responses derived from the color-coded regions in (C) following motion-artifact compensation. (E) Polar plot of average Ca^2+^ responses induced with moving grating stimulation from the color-coded neurons shown in (C). (F–I) Multi-layer, multi-frame imaging of the activity of long neuronal processes situated in a z range of over 500 μm. (F) Schematic of the measurements: multiple frames of different sizes and at any position in the scanning volume can be used to capture activities. (G) A layer V pyramidal neuron recorded. (H) Side view of the neuron shown in (G). Color-coded frames indicate the position of the simultaneously imaged squares. (I) Ca^2+^ transients calculated from the color-coded frames indicated in (G) are shown following motion correction with subpixel resolution. Transients were induced by moving grating stimulation in time periods shown in gray. See also Movies S8 and S9.

In order to preserve somatic fluorescence information during movement, we extended each scanning point to small squares (and, in other sets of measurements below, to small cubes). We can use the two main strategies described above to set the orientation of squares to be optimal for motion correction: namely, we can set the squares to be either parallel to the direction of motion or to be parallel to the nominal focal plane of the objective (Figure 4A). This second strategy will be demonstrated here. Similar to 3D ribbon scanning, we can generate a 2D projection of the 3D data during multi-layer, multi-frame recording, even during image acquisition, by simply arranging all the squares, and hence each soma, into a “chessboard” pattern for better visualization and movie recording (this version of multi-layer, multi-frame imaging is called “chessboard” scanning; Figure 4B; Movie S8). Similar to the 3D ribbon scanning, here we calculated the average brain displacement vector as a function of time and subtracted it from all frames to correct motion artifacts. Finally, we could select subregions from the 2D projection, calculate the corresponding Ca^2+^ transients as above (Figures 4C and 4D; Movie S9), and detect orientation- and direction-sensitive neurons with moving grating stimulation (Figure 4E; Movie S9). Chessboard scanning combines the advantage of low phototoxicity of low-power temporal oversampling (LOTOS) (Chen et al., 2011) with the special flexibility of the 3D scanning capability of AO microscopy by allowing simultaneous imaging along multiple small frames placed in arbitrary locations in the scanning volume with speeds exceeding that of resonant scanning (Supplemental Note).

### Multi-Layer, Multi-Frame Imaging of Long Neuronal Processes

Multi-layer, multi-frame scanning can also be used to measure neuronal processes (Figure 4F). Because the total z-scanning range with GECIs was extended to over 650 μm, we can, for example, simultaneously image apical and basal dendritic arbors of layer II/III or V neurons, or follow the activity of dendritic trees in this z-scanning range. To demonstrate the large dendritic imaging range, we selected a GCaMP6f-labeled layer V neuron from a sparsely labeled V1 network (Figure 4G). Visual stimulation-induced dendritic and somatic Ca^2+^ responses were simultaneously imaged at 30 Hz in multiple frames situated at 41 different depth levels over a 500 μm z range in an awake animal (Figures 4H). Motion artifacts along the x and y axes were eliminated from frames as above by subtracting the time-dependent net displacement vector. Finally, we derived the Ca^2+^ transients for each ROI (Figure 4I).

**Figure 5:**
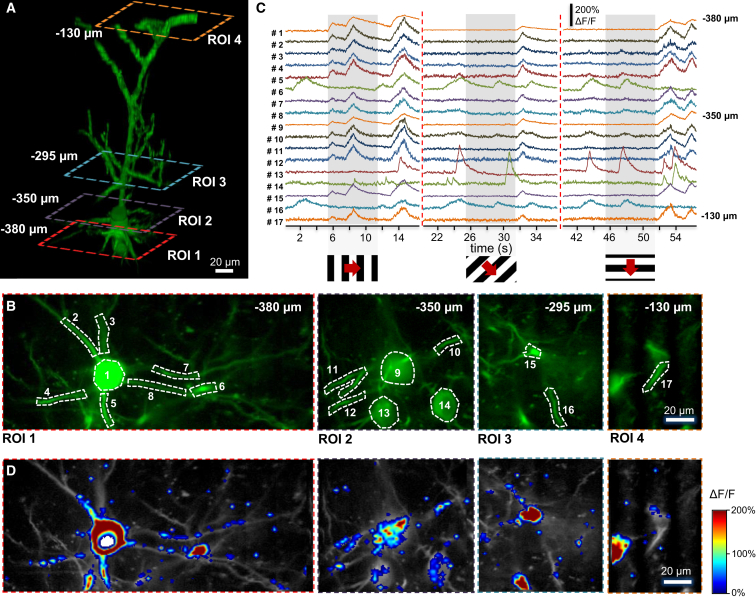
Multi-Layer Imaging Of Layer II/III Pyramidal Neurons and Their Dendritic Arbor during Visual Stimulation (A) Three-dimensional view of a layer II/III neuron labeled with the GCaMP6f sensor. Rectangles indicate four simultaneously imaged layers (ROI 1–4). Numbers indicate distances from the pia mater. Somata and neuronal processes of the three other sparsely labeled V1 neurons situated in the same scanning volume were removed from the z stack for clarity. (B) Average baseline fluorescence in the four simultaneously measured layers. (C) Representative Ca^2+^ transients were derived from the numbered subregions shown in (B) following motion artifact elimination. Responses were induced by moving grating stimulation into three different directions at the temporal intervals indicated with gray shadows. (D) The averaged baseline fluorescence images from (B) are shown in grayscale and were overlaid with the color-coded relative Ca^2+^ changes (ΔF/F). See also Movie S10.

Naturally, the multi-layer, multi-frame scanning method is not limited to a single dendrite of a single neuron; rather, we can simultaneously image many neurons with their dendritic (or axonal) arbor. To show this, we selected four layers of neuropil labeled with the GCaMP6f sensor using a nonspecific AAV vector and recorded activity at 101 Hz simultaneously in four layers (Figures 5A and 5B; Movie S10). Following motion artifact elimination, the maximal relative fluorescence changes were overlaid on the background fluorescence images (Figure 5D). To show an alternative quantitative analysis, we also calculated Ca^2+^ transients (Figure 5C) from some exemplified somatic and dendritic ROIs (Figure 5B).

### Volume Scanning with Multi-Cube and Snake Scanning

**Figure 6:**
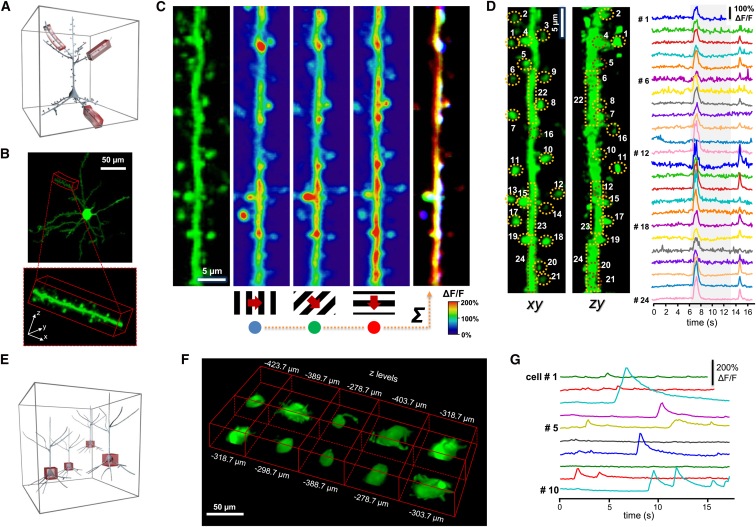
Fast 3D Volume Imaging of Dendritic and Somatic Activity in Behaving Animals (A–D) Snake scanning. (A) Schematic of the 3D measurement. Three-dimensional ribbons selected for 3D scanning can be extended to 3D volume elements (3D “snakes”) to fully preserve fluorescence information during movement (Movie S11). (B) z projection of a pyramidal neuron in the V1 region labeled with GCaMP6f sensor using sparse labeling. (C) Fast snake scanning was performed at 10 Hz in the selected dendritic region shown in (B). Fluorescence data were projected as a function of the distance along the longitudinal and the transverse directions, and then data were projected at maximal intensity along the second transverse direction. Left, average of 30 frames from the recorded video. Middle, peak Ca^2+^ responses following visual stimulation. Right, relative fluorescence responses for each of the three different stimuli were transformed to either red, green, or blue and summed; the result is shown as an RGB image. (D) The same dendritic segment as in (B) shown in x-y and z-y plane projections. Right, representative spontaneous Ca^2+^ responses derived from the coded subvolume elements. Transients were derived following 3D motion correction. (E–G) Multi-cube scanning. (E) Schematic of the 3D multi-cube measurements. (F) Volume-rendered image of ten representative cubes selecting individual neuronal somata for simultaneous 3D volume imaging (Movie S12). (G) Following 3D motion correction, Ca^2+^ transients were derived from the ten cubes shown in (F). See also Movies S11 and S12.

Our data demonstrated that even though the brain moves along all three spatial dimensions, we could still preserve fluorescence information and effectively eliminate motion artifacts by scanning at reduced dimensions, along surface elements, in 3D. However, under some circumstances, for example, in larger animals or at certain surgery or behavioral protocols, the amplitude of motion can be larger than the z dimension of the PSF and the missing third scanning dimension cannot be compensated for. To sufficiently preserve fluorescence information even in these cases, we may regain the missing scanning z dimension by extending the surface elements to volume elements by using an automatic algorithm until we reach the required noise elimination efficiency for measurements. To demonstrate this in two examples, we extended 3D ribbons to folded cuboids (called “snake scanning”; Figure 6A) and multi-frames to multi-cuboids (called “multi-cube scanning”). A spiny dendritic segment of a GCaMP6f-labeled layer II/III neuron was selected from a sparsely labeled V1 region of the cortex for snake scanning (Figure 6B). According to the z stack taken at the beginning, we selected guiding points, interpolated a 3D trajectory, and generated a 3D ribbon that covered the whole segment as described above. Then, we extended the ribbon to a volume and performed 3D snake scanning from the selected folded cuboid (Figures 6A–6D; Movie S11). Three-dimensional Ca^2+^ responses were induced by moving grating stimulation and were projected into 2D as a function of distances along the dendrite and along one of the perpendicular directions (Figure 6C). Finally, data were maximal-intensity projected along the second (and orthogonal) perpendicular axis to show average responses for three moving directions separately and together, following motion correction (Figure 6C). Alternatively, we were able to avoid maximal intensity projection to a single plane by transforming the folded snake form into a regular cube (Figure 6D). In this representation, Ca^2+^ transients could be calculated from different subvolumes (Figure 6D). Note that due to the preserved good spatial resolution, we can clearly separate each spine from each other and from the mother dendrite in moving animals. Therefore, we can simultaneously record and separate transients from spines even when they are located in the hidden and overlapping positions that are required to precisely understand dendritic computation (Figure 6D).

To demonstrate the second volume scanning method, multi-cube imaging, we simply extended frames to small cubes (Figure 6E) and added a slightly larger z dimension than the sum of the z diameter of somata and the peak z movement to preserve all somatic fluorescence points during motion. Simultaneous measurements of ten GCaMP6f-labeled somata were performed from 8.2 up to 25.2 Hz using relatively large cubes (each cube was between 46 × 14 × 15 voxels and 46 × 32 × 20 voxels, where one voxel was 1.5 × 3 × 4 μm and 1.5 × 1.5 × 4 μm). This spatial and temporal resolution made it possible to resolve the subcellular Ca^2+^ dynamic (Movie S12). We can further increase the scanning speed or the number of recorded cells inversely with the number of 3D drifts used to generate the cubes. For example, 50 somata can be recorded at 50 Hz when using cubes made of 50 × 10 × 5 voxels. Similar to multi-frame recordings, ROIs can be ordered next to each other for visualization (Figure 6F). Transients were derived from each cube using small subvolumes (Figure 6G). As above, here we calculated the net displacement vector and corrected subvolume positions at each time point during calculation of the Ca^2+^ transient in order to eliminate motion. We found that the use of volume scanning reduced the amplitude of motion artifacts in Ca^2+^ transients by 19.28- ± 4.19-fold during large-amplitude movements in behaving animals (Figures S9F and S9G). These data demonstrate that multi-cube and snake scanning can effectively be used for the 3D measurement of neuronal networks and spiny dendritic segments in multiple subvolumes distributed over the whole scanning volume. Moreover, these methods are capable of completely eliminating even large-amplitude motion artifacts.

### Multi-3D Line Scanning

**Figure 7:**
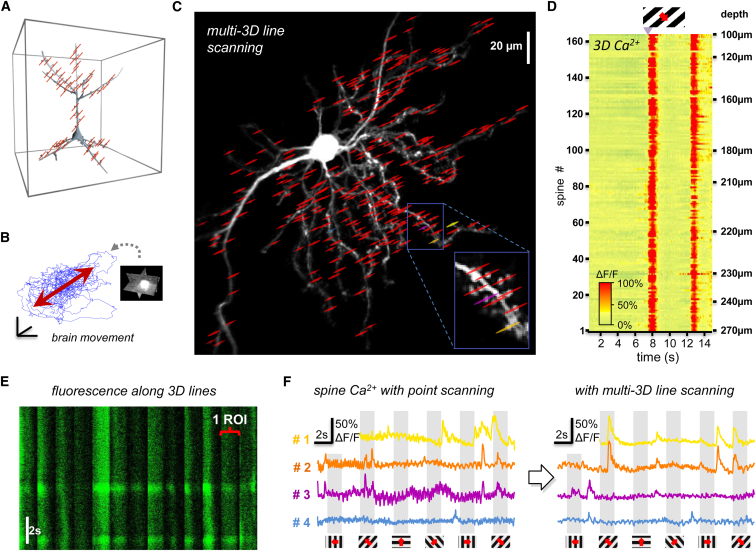
Multi-3D Line Scanning of Spine Assemblies in Behaving Animals (A) Schematic of the measurement. Each scanning line is associated with one spine. (B) Amplitude of brain motion (blue) was recorded by the fast 3D motion-detection method. Average motion direction is shown in red. Scale bars, 50 nm. (C) z projection of a layer II/III pyramidal cell, labeled with GCaMP6f. Red lines indicate the scanning lines running through 164 pre-selected spines. All scanning lines were set to be parallel to the average motion shown in (B). (D) Corresponding 3D Ca^2+^ responses. (E) Exemplified individual raw Ca^2+^ transients recorded along 14 spines. Note the movement artifacts in the raw fluorescence. (F) Exemplified Ca^2+^ transients measured using point scanning (left) and multi-3D line scanning (right). Note the improvement in the SNR when multi-3D line scanning was used. Transients were induced with moving grating stimulation.

In the previous sections, we extended 1D scanning points to 2D or 3D objects. In this section, we extend scanning points along only one dimension to perform measurements at a higher speed (Figure 7). We found that in many experiments, sample movement is small, and brain motion can be approximated with a movement along a single 3D trajectory (Figure 7B). In this case, we can extend each point of 3D random-access point scanning to only multiple short 3D lines instead of multiple surface and volume elements (Figure 7A). In the first step, we selected points from the z stack. Next, we recorded brain motion to calculate the average trajectory of motion, as above. In the third step, we generated short 3D lines with 3D DRIFT AO scanning to each pre-selected point in such a way that the center of the lines coincided with the pre-selected points, and the lines were set to be parallel to the average trajectory of motion (Figures 7B and 7C). Finally, we simultaneously detected the activity of 169 spines along the 3D lines (Figures 7C and 7E). If we switched back from the multi-3D line scanning mode to the classical 3D point-by-point scanning mode, oscillations induced by heartbeat, breathing, and physical motion appeared immediately in transients (Figure 7F). These data showed that in cases when the amplitude of the movement is small and mostly restricted to a 3D trajectory, we can effectively use multi-3D line scanning to rapidly record over 160 dendritic spines in behaving animals.

### Advantage of the Different Scanning Modes

**Figure 8:**
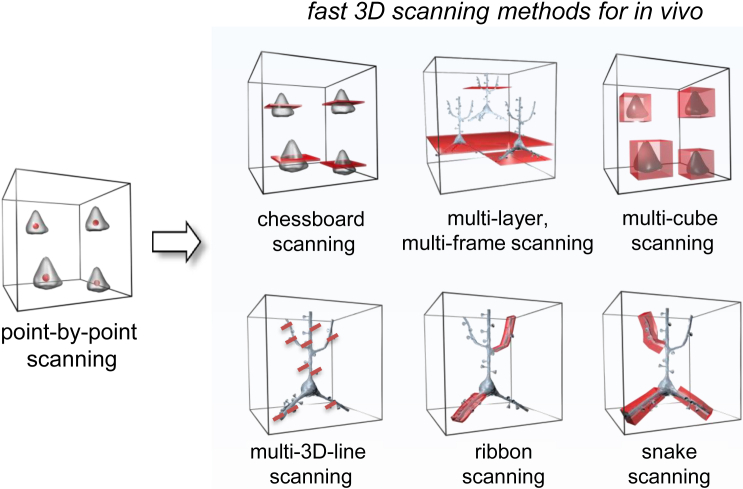
Summary of Fast 3D Scanning Methods Developed for In Vivo Imaging in Behaving Mice Schematics comparing the currently used 3D random-access point-by-point scanning method with the new 3D scanning methods developed for imaging the moving brain of behaving animals. Red points, lines, and surface and volume elements illustrate the ROIs selected for measurements. See also Figure S8I.

We have described six novel laser scanning methods for 3D imaging using drift AO scanning. These methods have different application fields based on how they are suited to different brain structures and measurement speed. The fastest method is multi-3D line scanning, which is as fast as random-access point-by-point scanning and can be used to measure spines or somata at 53 kHz per ROI (Figure 8). The number of ROIs is only limited by MATLAB and the memory of the PC (could be >10^5^). In the second group, multi-layer, multi-frame imaging; chessboard scanning; and 3D ribbon scanning can measure up to about 5.3 kHz per ROI along long neuronal processes and somata. Finally, the two volume scanning methods, multi-cube scanning and snake scanning, allow measurement of up to about 1.3 kHz per volume element and are ideal for measuring somata and spiny dendritic segments, respectively. The two volume scanning methods provide the best noise elimination capability because fluorescence information can be maximally preserved.

Finally, we quantified how the increased SNR of the new scanning strategies improves single AP resolution from individual Ca^2+^ transients when a large number of neurons were simultaneously recorded in the moving brain of behaving animals (Figure S8I). Chessboard scanning; multi-cube scanning; or multi-layer, multi-frame imaging in behaving animals improved the SD of the Ca^2+^ responses by a factor of 14.89 ± 1.73, 14.38 ± 1.67, and 5.55 ± 0.65, respectively (n = 20), as compared to 3D random-access point scanning (Figure S8I). Therefore the SD of the motion-artifact-corrected Ca^2+^ responses became smaller than the average amplitude of single APs, which made single AP detection possible in neuronal network measurements in behaving animals. Quantitatively, amplitudes of the motion-induced somatic fluorescence transients were 55.35% ± 4.67% larger than single AP-associated Ca^2+^ responses (Figures S7A–S7E, S7G, and S7H), and this value dropped to 1.66% ± 0.51% following motion correction, showing the efficiency of the method (Figure S7F). Single AP detection was not possible with 3D random-access point scanning during running because the SD of Ca^2+^ responses was 4.85- ± 0.11-fold higher than the amplitude of single APs (Figure S8I). It is important to note that in this paragraph, we based all quantifications on an a priori knowledge about AP timing, which artificially reduced the SNR improvement of our method because the detection of the timing of single APs is more sensitive to the increase of noise, and a quantitative comparison for spike timing estimation would result in a higher effect.

## Discussion

There are several ways to extend single scanning points to surface and volume elements with 3D DRIFT AO scanning, for the combinations of different 3D lines, surfaces, and volume elements are almost unlimited. In this work, we demonstrate new scanning methods for in vivo measurements in large scanning volumes: 3D ribbon scanning; multi-3D line scanning; snake scanning; multi-layer, multi-frame imaging; chessboard scanning; and multi-cube scanning (Figure 8). Each of them is optimal for a different neurobiological aim. For example, the first three are optimal for different dendritic measurements, while the last two are best for somatic recordings; the surface scanning methods are optimized for speed, while the methods based on volume imaging are optimized for large amplitude movements. Volume or area scanning used in these methods allows motion artifact correction on a fine spatial scale and, hence, the in vivo measurement of fine structures in behaving animals. Therefore, we can preserve fluorescence information from the pre-selected ROIs during 3D measurements even in the brain of behaving animals, while maintaining the 10–1,000 Hz sampling rate necessary to resolve neural activity at the individual ROIs.

### Benefits of the New 3D Methods in Neuroscience

We demonstrate several further technical advances over previous 3D methods in the present work: (1) it enables a scanning volume with GECIs that is more than two orders of magnitude larger than previous realizations, while the spatial resolution remains preserved; (2) it offers a method of fast 3D scanning in any direction, with an arbitrary velocity, and without any sampling rate limitation during drifts; (3) it is free of mechanical and electrical inertias, which makes it possible to flexibly select surface and volume elements matching multiple somatic and dendritic locations, thereby effectively focusing measurement time to the ROIs; (4) it compensates fast motion artifacts in 3D to preserve high spatial resolution, characteristic of two-photon microscopy, during 3D surface scanning and volume imaging even in behaving animals; and (5) it provides good SNR and enables generalization of the low-power temporal oversampling (LOTOS) strategy of 2D raster scanning (Chen et al., 2011) in fast 3D AO measurements to reduce phototoxicity. When raster scanning of the entire cubature is replaced with 3D DRIFT AO scanning, the $v_{gain}*{\left(SNR_{gain}\right)}^{2}$ is proportional to the ratio of the overall cubature versus the volume covered by the scanned ROIs, and this ratio can be high (over 10^6^; see Supplemental Note for quantitative examples).

These technical achievements enabled the realization of the following fast 3D measurements and analysis methods in awake, behaving animals: (1) simultaneous functional recording of over 150 spines; (2) fast parallel imaging of activity in over twelve spiny dendritic segments; (3) precise separation of fast signals in space and time from each individual spine (and dendritic segment) from the recorded volume, in which signals overlap with the currently available methods; (4) simultaneous imaging of large parts of the dendritic arbor and neuronal networks in a z-scanning range of over 650 μm; (5) imaging a large network of over 100 neurons at subcellular resolution in a scanning volume of up to 500 × 500 × 650 μm with the SNR more than an order of magnitude larger than for 3D random-access point scanning; and (6) more than 10-fold better single AP resolution during motion in neuronal network measurements.

The limits of our understanding of neural processes lie now at the fast dendritic and neuronal activity patterns occurring in living tissue in 3D, and their integration over larger network volumes. Until now, these aspects of neural circuit function have not been measured in awake, behaving animals. Our new 3D scanning methods, with preserved high spatial and temporal resolution, provide the missing tool for these activity measurements. Among other advantages, it will be possible to use these methods to investigate the origin of dendritic regenerative activities (Schiller et al., 2000, Larkum et al., 2009); the propagation of dendritic spikes (Katona et al., 2011, Chiovini et al., 2014, Fernández-Alfonso et al., 2014); spatiotemporal clustering of different input assemblies (Larkum and Nevian, 2008, Katona et al., 2011); associative learning (Kastellakis et al., 2015); the spatial and temporal structure of the activity of spine, dendritic, and somatic assemblies (Ikegaya et al., 2004, Takahashi et al., 2012, Villette et al., 2015); receptive field structures (Ohki et al., 2005); and function and interaction of sparsely distributed neuronal populations, such as parvalbumin-, somatostatin-, and VIP-expressing neurons (Klausberger and Somogyi, 2008, Kepecs and Fishell, 2014). Importantly, these complex functional questions can be addressed using our methods at the cellular and subcellular level, and simultaneously at multiple spiny (or aspiny) dendritic segments and at the neuronal network level in behaving animals.

### Simultaneous 3D Imaging of Apical and Basal Dendritic Activity during Motion

Two-dimensional in vivo recordings of spine Ca^2+^ responses have already been realized in anesthetized animals (Jia et al., 2010) and even in running animals, but in these works only a few spines were recorded with a relatively low SNR (Marvin et al., 2013, Cichon and Gan, 2015). However, fast 2D and 3D imaging of large spine assemblies and spiny dendritic segments in awake, running, and behaving animals has remained an important challenge because locomotion can more than double firing rate in most neurons (Niell and Stryker, 2010, Fu et al., 2014). Moreover, the majority of the top-down and bottom-up input integration occurs in complex, distant apical and basal dendritic segments separated by distances of several hundred micrometers in 3D (Schiller et al., 2000, Magee and Johnston, 2005, Larkum et al., 2009, Smith et al., 2013, Phillips, 2015). The maximal, over 1,000 μm z-scanning range of AO microscopy (Katona et al., 2012), which was limited during in vivo measurements with GECIs to about 650 μm by the maximal power of the currently available lasers, already permitted the simultaneous measurement of these apical and basal dendritic segments of layer II/III neurons and dendritic segments of layer V neurons in a range of over 500 μm during running periods, or in different behavioral experiments (Harvey et al., 2012, Hangya et al., 2015) where motion-induced fluorescence transients had similar amplitude and kinetics as behavior-related Ca^2+^ transients without motion compensation. Although 2D imaging in anesthetized animals can capture long neuronal processes (Jia et al., 2010), the location of horizontally oriented long segments is almost exclusively restricted to a few layers (for example, to layer I), and in all other regions we typically see only the cross-section or short segments of obliquely or orthogonally oriented dendrites. The main advantage of the multi-3D ribbon and snake scanning methods is that any ROI can be flexibly selected, shifted, tilted, and aligned to the ROIs; this means that complex dendritic integration processes can be recorded in a spatially and temporally precise manner. In summary, the 3D scanning methods demonstrated here, alone or in different combinations, will add new tools that have long been missing from the toolkit of neurophotonics for recording dendritic activity in behaving animals.

### Compensation of Movement of the Brain

Although closed-loop motion artifact compensation, with three degrees of freedom, has already been developed at low speed (≈10 Hz) (Cotton et al., 2013), the efficiency of the method has not been demonstrated in awake animals, in dendritic spine measurements, or at higher speed. Moreover, due to the complex arachnoidal suspension of the brain, and due to the fact that blood vessels generate spatially inhomogeneous pulsation in their local environment, the brain also exhibits significant deformation, not merely translational movements, and, therefore, the amplitude of displacement could be different in each and every subregion imaged. This is crucial when small-amplitude somatic responses (for example, single or a few AP-associated responses) or small structures such as dendritic spines are measured. Fortunately, our 3D imaging and the corresponding analysis methods also allow compensation with variable amplitude and direction in each subregion imaged, meaning that inhomogeneous displacement distributions can therefore be measured and effectively compensated in 3D.

The efficiency of our 3D scanning and motion artifact compensation methods is also validated by the fact that the SD of individual somatic Ca^2+^ transients was largely reduced (over 10-fold) and became smaller than the amplitude of a single AP-induced transient, especially when multi-cube or chessboard scanning was used. This allows single AP resolution in the moving brain of behaving animals using the currently favored GECI, GCaMP6f.

### Deep Scanning

Although there are many effective methods for fast volume scanning such as SPIM (Mickoleit et al., 2014), DSLM-SI, SimView (Tomer et al., 2012), SCAPE (Bouchard et al., 2015), and OPLUL (Kong et al., 2015), the z-scanning range of in vivo functional measurements in mice is currently limited to about 130 μm in the case of these technologies. Imaging deep-layer neurons is possible only by either causing mechanical injury (Andermann et al., 2013) or using single-point two-photon or three-photon excitation, which allows fluorescent photons scattered from the depth to be collected (Helmchen and Denk, 2005, Horton et al., 2013). One of the main advantages of 3D DRIFT AO scanning is that it is based on whole-field detection, and the extended fast z-scanning range is over 1,100 μm in transparent samples and about 650 μm during functional in vivo imaging in mouse brain (limited by the power of the laser). Using adaptive optics and regenerative amplifiers can improve resolution and SNR at depth (Mittmann et al., 2011, Wang et al., 2014). One of the main perspectives of the 3D scanning methods demonstrated here is that the main limitation to reach the maximal z-scanning ranges of over 1.6 mm (Theer et al., 2003, Kobat et al., 2011, Horton et al., 2013) is the relatively low laser intensity of the currently available lasers. Supporting this over a 3 mm z-scanning range has already been demonstrated with 3D AO imaging in transparent samples, where intensity and tissue scattering are not limiting (Katona et al., 2012). Therefore, in the future novel, high-power lasers, combined with fast adaptive optics and new red-shifted sensors, may allow a much larger 3D scanning range to be utilized, which will, for example, permit the measurement of the entire arbor of deep-layer neurons or 3D hippocampal imaging, without removing any tissue from the cortex.

Although there are several different arrangements of passive optical elements and the four AO deflectors with which it is possible to build microscopes for fast 3D scanning (Katona et al., 2012, Cotton et al., 2013, Chiovini et al., 2014, Fernández-Alfonso et al., 2014), all of these microscopes use drift compensation with counter-propagating AO waves at the second group of deflectors. The scanning methods demonstrated here can therefore easily be implemented in all 3D AO microscopes. Moreover, 3D AO microscopes could be simplified and used as an upgrade in any two-photon system. Hence, we anticipate that our new methods will open new horizons in high-resolution in vivo imaging in behaving animals.

## Experimental Procedures

### Mice, AAV Labeling, Surgical Procedure, and Electrophysiology

All experimental protocols were approved by the Animal Care and Experimentation Committee of the Institute of Experimental Medicine of the Hungarian Academy of Sciences (approval reference numbers PEI/001/194-4/2014 and PEI/001/1771-2/2015). The surgical process, electrophysiological recordings, and visual stimulation were similar to that described previously (Katona et al., 2012, Chiovini et al., 2014), with some minor modifications. For population imaging, we expressed GCaMP6s or GCaMP6f with AAV vectors. See Figure S10, Movie S13, and Supplemental Experimental Procedures for further details.

### 3D DRIFT AO Scanning

We derived a one-to-one relationship between the focal spot coordinates and speed, and the chirp parameters of the four AO deflectors to move the focal spot along any 3D line, starting at any point in the scanning volume (Movie S1). We need the following three groups of equations: (1) the simplified matrix equation of the AO microscope (Figure S1C; Equations S1–S4); (2) the basic equation of the AO deflectors (Equation S5); and (3) temporally non-linear chirps for the acoustic frequencies (*f*) in the deflectors deflecting in the x-z (and y-z) plane,

(Equation 1) $$f_{i}\left(x,t\right)=f_{xi}\left(0,0\right)+\left(b_{xi}*\left(t-\frac{D}{2*v_{a}}-\frac{x}{v_{a}}\right)+c_{xi}\right)*\left(t-\frac{D}{2*v_{a}}-\frac{x}{v_{a}}\right)$$

(where *i* = 1 or *i* = 2 indicates the first and second x deflector, *D* is the diameter of the AO deflector, and *v*_*a*_ is the propagation speed of the acoustic wave within the deflector). When we calculate and then combine the expression *z*_*x*_*(t)* (Equation S17) with *x*_*0*_*(t)*, the similarly calculated *z*_*y*_*(t)*, and *y*_*0*_*(t)*, and add all the required initial positions (*x*_*0*_*, y*_*0*_*, z*_*0*_) and speed parameter values (*v*_*x0*_*, v*_*y0*_*, v*_*zx0*_ *= v*_*zy0*_) of the focal spot, we can determine all the parameters required to calculate the non-linear chirps according to Equation 1 in the four AO deflectors (*Δf*_*0x*_ *= f*_*1x*_*(0,0) − f*_*2x*_*(0,0), b*_*x1*_*, b*_*x2*_*, c*_*x1*_*, c*_*x2*_ and *Δf*_*0y*_ *= f*_*1y*_*(0,0) − f*_*2y*_*(0,0), b*_*y1*_*, b*_*y2*_*, c*_*y1*_*, c*_*y2*_). The calculations and AO driver signal generation are detailed in the Supplemental Experimental Procedures (Equations S1–S70; Data S1, S2, and S3; Figure S3). We have summarized the calculations in Table S1.

### Data Analysis and Statistics

The statistical difference was measured using the Student’s t test (^∗^p < 0.05, ^∗∗^p < 0.01, ^∗∗∗^p < 0.001). If not otherwise indicated, data are presented as means ± SEM. Raw fluorescence (*F*) recorded along ribbons in 3D was spatially normalized and then projected onto a 2D plot by applying the formula *ΔF/F = (F(d*_*L*_*,d*_*tr1*_*,t) – F*_*0*_*(d*_*L*_*,d*_*tr1*_*))/F*_*0*_*(d*_*L*_*,d*_*tr1*_*)*, where *d*_*L*_ and *d*_*tr1*_ indicate the longitudinal and transversal distance along the ribbon; see Supplemental Experimental Procedures for further details.

## Author Contributions

Optical development was performed by P.M., A.F., and M.V. Software was written by G.K. and K.Ó. Measurements were performed by G.S., L.J., Z.S., G.J., and B.C. Analysis was carried out by G.S., L.J., G.K., P.M., K.Ó., M.V., and B.R. This manuscript was written by B.R., G.S., L.J., G.K., P.M., and T.T. with comments from all authors. B.R. and G.K. supervised the project and designed the scanning strategies.

## Supplemental Information

Document S1. Supplemental Experimental Procedures, Figures S1–S10, and Tables S1 and S2

Movie S1. Validation of 3D DRIFT AO Scanning by Bleaching a Polyhedron Shape in a Homogeneous Fluorescent Sample Related to Figure 1. Resolution and point stability of the 3D DRIFT AO scanning method was validated by “burning” a polyhedron shape in a homogeneous fluorescent sample using photo bleaching. Each edge of the polyhedron was scanned by a 3D line that was generated within a single AO-switch time period (33 μs). The precision and reproducibility of the 3D scanning were verified by a z stack performed at a much lower intensity. The movie shows the inverted fluorescence intensity of the z stack. Note the stability of the 3D lines during scanning.

Movie S2. Large AO 3D Scanning Volume Using GECIs Related to Figure 1. z stack from 650 to 100 μm under the pia mater in the visual cortex of a Thy1-Cre mouse was taken using the 3D DRIFT AO microscope. For cre-dependent expression of the GCaMP6f Ca^2+^ sensor, we used an AAV vector injected into the V1 region. Raw fluorescence data were normalized for radially inhomogeneous illumination as previously described (Katona et al., 2012).

Movie S3. Method of Selection of 3D Ribbons for Fast Scanning of Spiny Dendritic Segments Related to Figure 1. The video demonstrates how guiding points are selected, how the 3D trajectory is fitted according to the guiding points, and, finally, how the plan of the ribbon is generated for 3D ribbon scanning. See also the “Selection of the 3D trajectory and the ribbons for 3D ribbon and snake scanning” section in the Supplemental Experimental Procedures.

Movie S4. 3D Ribbon Scanning of a Spiny Dendritic Segment Related to Figure 1. A 140 μm GCaMP6-labeled spiny dendritic segment situated in a 60 μm range of z-scanning was imaged using 3D ribbon scanning.

Movie S5. 3D Ribbon Scanning of a Spiny Dendritic Segment during Visual Stimulation Related to Figure 1. Similar example as Movie S4. A GCaMP6-labeled spiny dendritic segment from a different layer II/III neuron was imaged using 3D ribbon scanning.

Movie S6. Multi-3D Ribbon Scanning: Raw Data Recorded in Behaving Animal Related to Figures 2 and 3. Twelve spiny dendritic segments of a GCaMP6-labeled layer II/III pyramidal cell located in V1 were simultaneously imaged using 3D ribbon scanning. Note the large and spatially inhomogeneous movement artifacts during recording of activity in an awake animal.

Movie S7. Multi-3D Ribbon Scanning Movie Before and After the Correction of Motion Artifacts Related to Figure 3. The first block is the same movie as Movie S5. In the second block of the movie, motion artifacts were eliminated by shifting back each frame of all regions by the local projection of the net displacement vector of the brain (see text for details). This correction was made with subpixel resolution. Note the small residual motion in the video. Finally, in the last block of the movie residual motion, artifacts were removed by repeating the cross-correlation-based displacement calculations and the consequent back shifts for each frame separately on all dendritic segments.

Movie S8. Representative Example of Cell Selection and Image Acquisition during Chessboard Scanning in 3D Related to Figures 2, 3, and 4. Populations of V1 neurons were labeled with GCaMP6f protein expressed by non-specific promoter. First, a z stack was obtained using the 3D AO microscope. Second, ROIs, here small squares centered on neuronal somata, were selected. Finally, the measured ROIs were arranged into the form of a chessboard. In this way, 3D activity was visualized during data acquisition and then stored as a 2D movie.

Movie S9. Three-Dimensional Recording Neuronal Networks in V1 with Chessboard Scanning during Visual Stimulation Related to Figure 4. The same populations of V1 neurons as in Movie S8. Activity was recorded in awake, head-restrained animals during visual stimulation. The upper left corner shows the video of raw fluorescence data arranged in chessboard form. The upper right corner shows the motion-corrected video converted to relative fluorescence changes (ΔF/F). In the lower left corner, the actual visual stimulation is presented, while in the lower right corner, the transients of nine exemplified neurons are shown during the actual visual stimulation.

Movie S10. Multi-Layer, Multi-Frame Imaging of Neuropil Activity with Over 100 Hz Temporal Resolution Related to Figure 5. V1 neurons were sparsely labeled using the GCaMP6f sensor and measured simultaneously in four different z planes (z_1_ = −380 μm, z_2_ = −350 μm, z_3_ = −295 μm, z_4_ = −130 μm) with 101 Hz temporal resolution during visual stimulation with moving gratings. Movies were converted to relative fluorescence changes (ΔF/F) and aligned next to each other.

Movie S11. Snake Scanning of a Spiny Dendritic Segment in the Moving Brain of a Behaving Mouse Related to Figure 6. V1 neurons were sparsely labeled using the GCaMP6f sensor. A spiny dendritic segment was selected for volume imaging using snake scanning at 10 Hz. Motion artifacts were removed and data are shown as relative fluorescence changes (ΔF/F) overlaid on the averaged baseline fluorescence.

Movie S12. Multi-Cube Imaging of Neuronal Activity Related to Figure 6. V1 neurons were sparsely labeled using the GCaMP6f sensor. Ten labeled neurons were centered in cubes. Volume imaging was performed simultaneously in the ten cubes with 10 Hz temporal resolution. Finally, motion artifacts were removed and data are shown as relative fluorescence changes (ΔF/F). Note the fine Ca^2+^ dynamic in the somatic and proximal dendritic segments.

Movie S13. z Stack of a GCaMP6f-Labeled Neuronal Population Related to Experimental Procedures. z stack from 0 to 820 μm under the pia mater in the visual cortex of a Thy1-Cre mouse was taken using 2D galvo scanning. Detectors were fixed to the objective arm (traveling detector system) to minimize the detection pathway and maximize detection of backscattered fluorescent light. For cre-dependent expression of the GCaMP6f Ca^2+^ sensor, we used an AAV vector injected into the V1 region. See Supplemental Experimental Procedures.

Data S1. Verilog Code of the Signal Generation Related to Experimental Procedures. Signal generation and high-speed communication with the DAC chip performed by the FPGA (see also Figure S3).

Data S2. MATLAB Function Calculating AO Driving Signal Related to Experimental Procedures. This MATLAB function returns the four center frequencies in MHz, the four frequency step speeds in MHz/s, and the four frequency derivate step values in MHz/s^2^ for any desired point in the field of view with any desired speed vector.

Data S3. MATLAB Function Calculating Register Values for the FPGA Related to Experimental Procedures. This MATLAB function returns the register values transferred to the FPGA for synthesis. Center frequencies, frequency steps, and frequency derivate steps calculated for each scanning segment are the inputs, while register values FR, FSR, and FDSR are the output.

Document S2. Article plus Supplemental Information
