# Supplementary material for: Fast 3D Imaging of Spine, Dendritic, and Neuronal Assemblies in Behaving Animals
Source: Neuron. 2016 Nov 23;92(4):723–38. doi: 10.1016/j.neuron.2016.10.002 (PMC5167293; doi:10.1016/j.neuron.2016.10.002)
Supplement: Document S1. Supplemental Experimental Procedures, Figures S1–S10, and Tables S1 and S2 [file mmc1.pdf]

Neuron, Volume *92*

Supplemental Information

Fast 3D Imaging of Spine, Dendritic, and Neuronal Assemblies in Behaving Animals

Gergely Szalay, Linda Judák, Gergely Katona, Katalin Ócsai, Gábor Juhász, Máté Veress, Zoltán Szadai, András Fehér, Tamás Tompa, Balázs Chiovini, Pál Maák, and Balázs Rózsa

Supplemental Information

Supplemental Figures

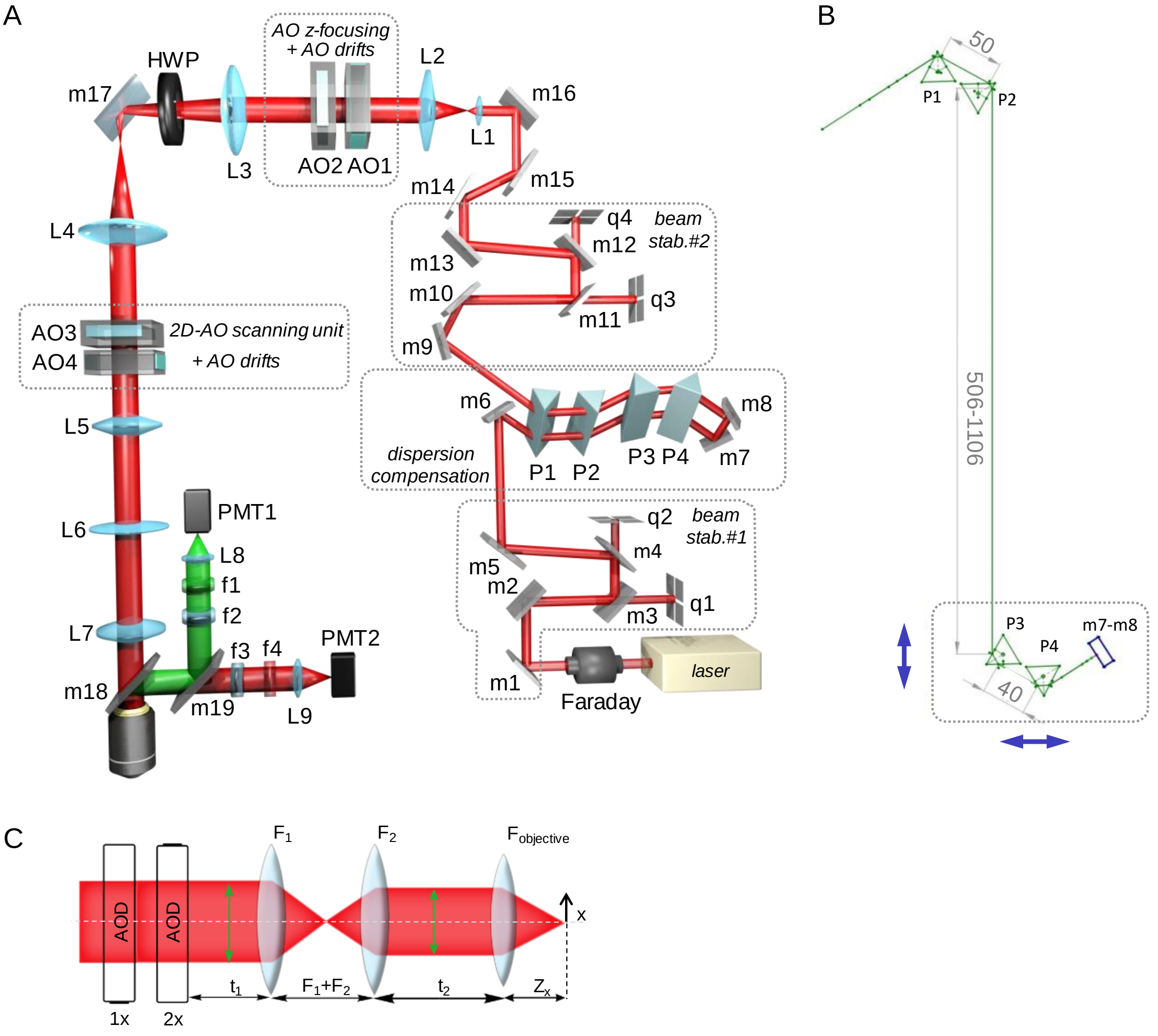

Figure S1, related to Figure 1. Block diagram of the 3D DRIFT AO microscope and four-prism compressor unit.

(A) A Faraday isolator (*Faraday*) eliminated coherent back reflections. Motorized mirrors (*m1, m2, m9,* and *m10*) stabilized the position of the beam on the surface of the quadrant detectors (*q1, q2, q3,* and *q4*) in the two beam stabilization units (*beam stab. #1* and *beam stab. #2*). Temporal dispersion compensation was adjusted with a motorized four-prism compressor (*P1-P4*). In contrast to the previous realization (Katona et al. 2012), a retro reflector (*m7* and *m8*) was used to make the

two laser beams, crossing the four prism sequence, parallel. In contrast, using only a single mirror, this combination preserved the Gaussian beam shape. The beam was then expanded by the *L1* and *L2* lenses. The z coordinates of the desired locations were targeted by two AO deflectors (*AO1* and *AO2*) optimized for diffraction efficiency and bandwidth. These generated two cylindrical lenses (*AO z-focusing* unit). A separate two-dimensional AO scanner unit (*AO3* and *AO4, 2D-AO scanning unit*) targeted the x and y coordinates. The *AO3* and *AO4* deflectors were introduced with chirped sine waves to generate lateral scanning drifts and also partially compensate the lateral drifts generated by the *AO1* and *AO2* deflectors. The joint work of the deflectors of the *AO z-focusing* and the *2D-AO scanning* units generated the drifts along 3D lines according to the **Supplemental Experimental Procedures**. A half wave plate (*HWP*) between the *2D-AO scanning* and *AO z-focusing* units ensures optimal incident polarization direction for maximum diffraction efficiency in the AO3 and AO4 deflectors. Angular dispersion compensation was provided by a spherical field lens (*L6*) in the second telecentric lens system (*L5* and *L7*). The backreflected fluorescent light was separated from the IR excitation by a dichroic mirror (*m18*) and then split into two parts ("green" and "red" channels) by a dichroic mirror (*m19*). In both channels, the remaining IR light was filtered out (*f2* and *f3*), then the light was collected by *L8* and *L9* lenses into the large aperture photomultipliers (*PMT1* and *PMT2*) following band-pass filtering (*f1* and *f4*). Optical design is detailed in *the 3D microscope design* section in the **Supplemental Experimental Procedures.**

(B) Schematic of the motorized four-prism compressor (indicated as *P1-P4*, *m7*, and *m8* in A; *m6* is not shown). Numbers are distances in mm. The blue square indicates the two retro mirrors (*m7* and *m8*) which back reflect the beam. Components in the gray dotted box were moved simultaneously and parallel to the blue arrows during wavelength tuning.

(C) The optical geometry of the 3D focusing system used for calculating fast 3D drifts along arbitrary directions in space. For the optical calculation we use a paraxial approximation of the whole AO microscope applied in two perpendicular planes (x-z and y-z planes) whose orientations are set by the deflection directions of the AO deflectors. Here we show the x-z plane.

See also the *Detailed description of the mathematical background of 3D DRIFT AO scanning* section in the **Supplemental Experimental Procedures**.

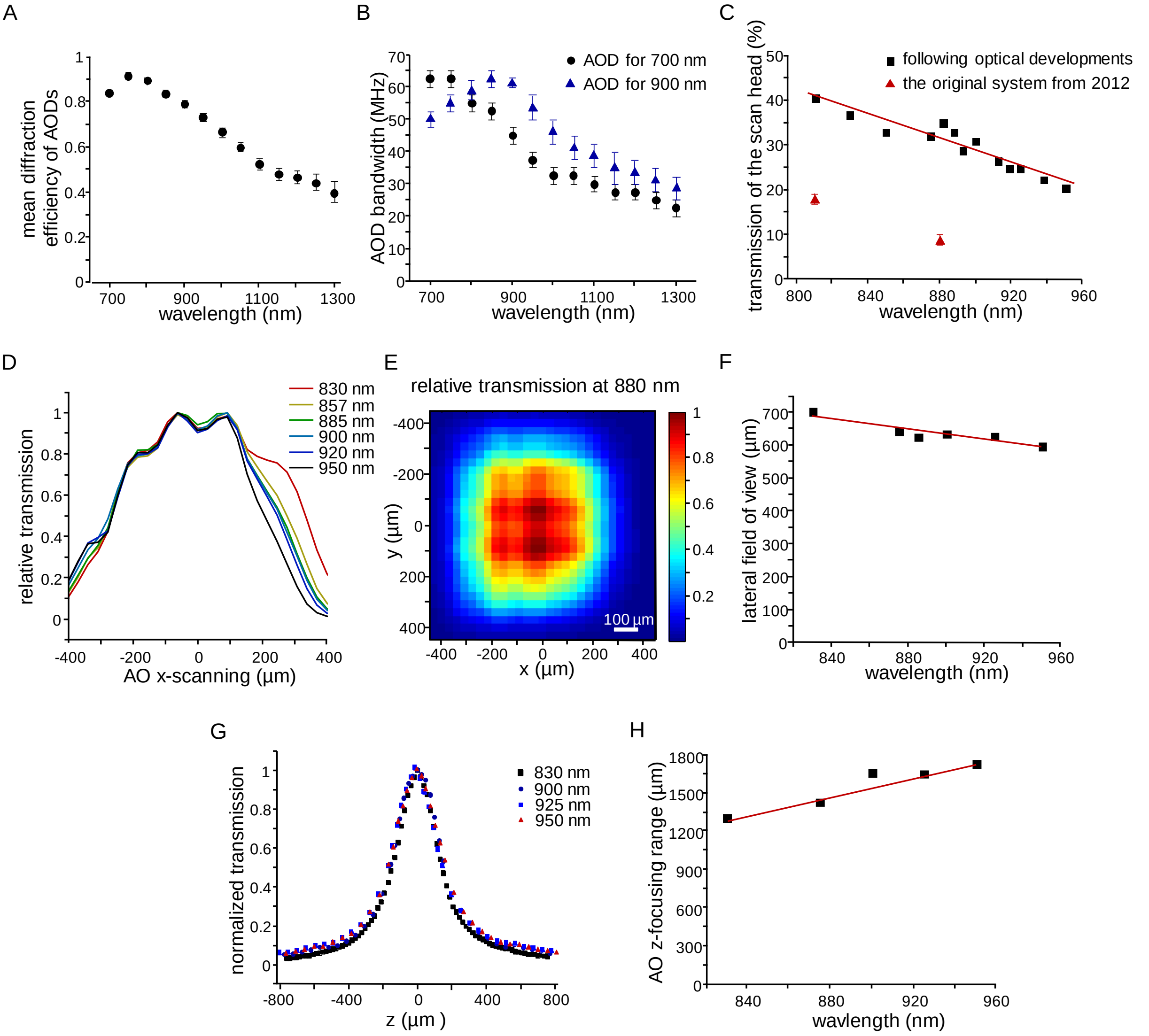

Figure S2, related to Figure 1. Characterization of the AO deflectors and 3D DRIFT AO scanning as a function of wavelength. (A) Mean diffraction efficiency (mean ± SEM) of AO deflectors as a function of wavelength. The mean diffraction efficiency was independent of crystal orientation: data from AO deflectors developed for different wavelengths were therefore pooled. (B) In contrast, AOD bandwidth did depend on crystal orientation. Full width at half maximum (FWHM) of diffraction efficiency as a function of wavelength for deflectors where the acoustic wave propagated at a higher angle (10°) relative to the [1,1,0] crystallographic axis (blue) was compared to the one of deflectors with a lower relative propagation angle (8.5°) (black). The first deflector with a 10° relative angle was optimized for 900 nm and the second for 700 nm. (C) Maximal transmission of the scan head as a function of wavelength (black). The maximal transmission was measured in the center (at x,y,z=0) and decreased as a function of wavelength. Red triangles show transmission of the scan head published earlier (Katona et al., 2012). (D) Normalized maximal transmission of the 3D AO scan head as a function of distance along the x axis at six different wavelengths. (E) Normalized intensity profile

shows lateral field of view in the nominal focal plane at 880 nm. (F) Full width at 30% of the maximum transmission for the x-profile as a function of wavelength. (G) Normalized transmission of the 3D AO scan head at different states of AO z focusing (from −800 µm to +800 µm) for four different wavelengths (830 nm, 900 nm, 925 nm, 950 nm). Note that the z profile of the transmission becomes wider as a function of increasing wavelength. (H) Full width at 5% of the maximum transmission for the normalized z-profiles (AO z-focusing range) as a function of wavelength.

See also the *Optical performance of the 3D DRIFT AO microscope* and the *AO deflectors and drivers* sections in the **Supplemental Experimental Procedures.**

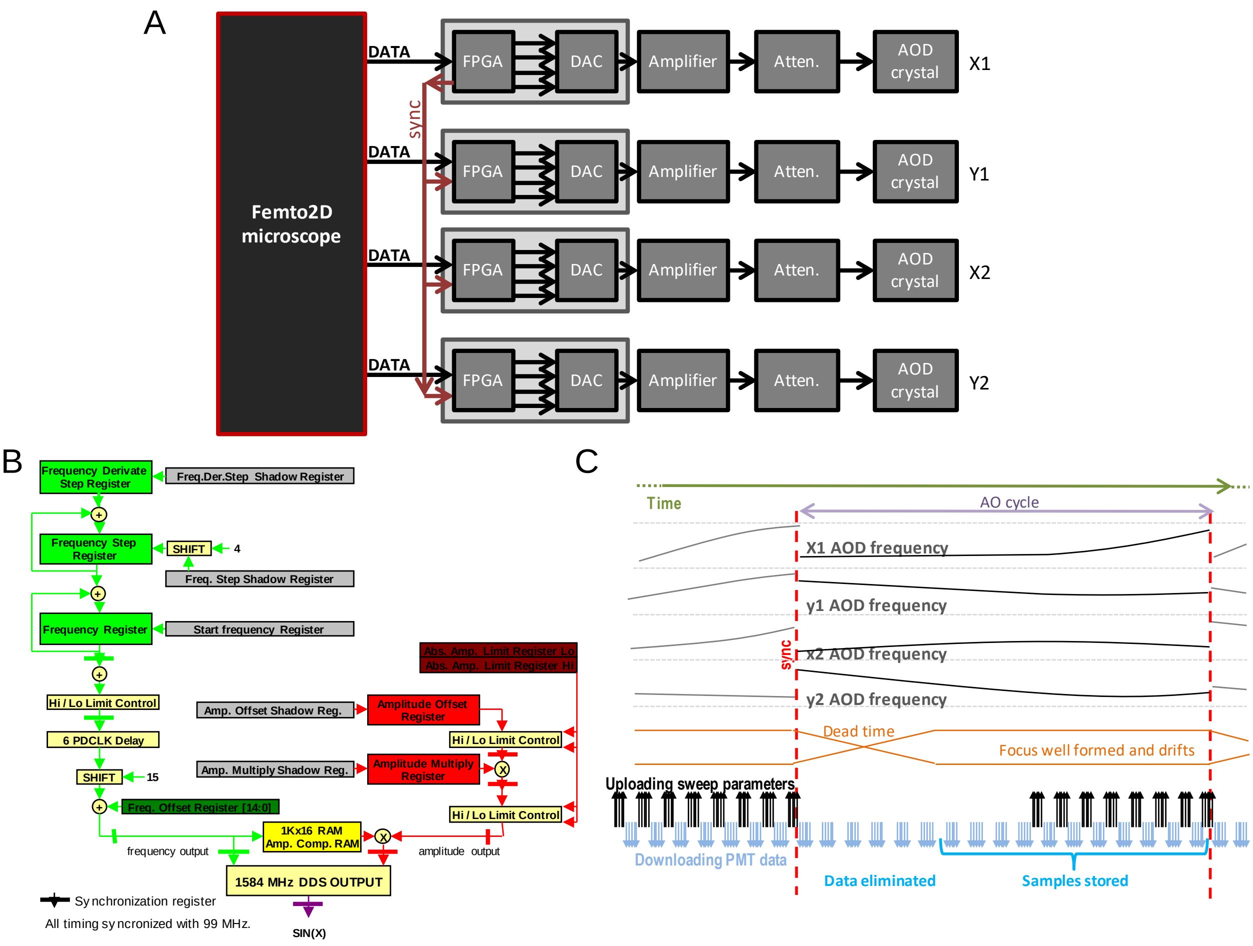

Figure S3, related to Figure 1. Electronics to generate driver signals for the AO deflectors.

(A) Architecture of the microscope control electronics of the AO deflectors of the AO z-focusing (AO1, AO2 in **Figure S1**) and the 2D-AO scanning (AO3, AO4 in **Figure S1**) units. *Amplifier* means the radiofrequency amplifier and *Atten.* is the radiofrequency attenuator (with –3dB attenuation). The precise synchronization of the high-speed output was provided by a separate digital line (sync) between each AO signal synthesis cards incorporating both the FPGA and the DAC chips.

(B) Block diagram of the frequency and amplitude generation registers and the related calculations within the FPGA.

(C) Input-output timing diagram of the 3D microscope. AO cycle starts from the *sync* signal. In the first part of the AO cycle, the focus is still not fully formed (*dead time*), therefore PMT data captured in this interval were eliminated. At the end of the cycle the AO scanning parameters for the next cycle are uploaded to the FPGA chips.

See also the *Signal generation and flow in the 3D DRIFT AO scanning two-photon microscope* section in the **Supplemental Experimental Procedures.**

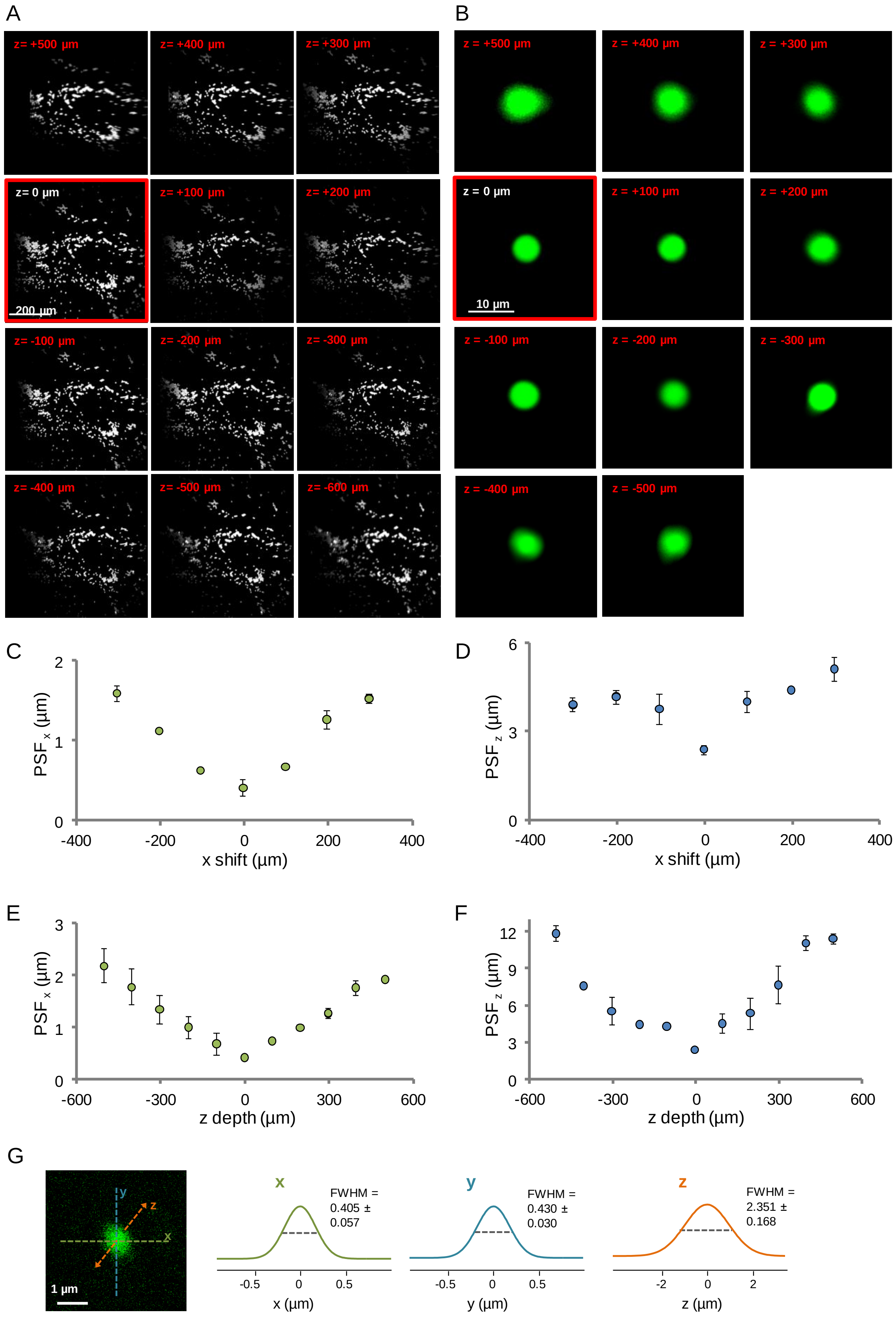
A
z= +500 µm
z= +400 µm
z= +300 µm
z= 0 µm
z= +100 µm
z= +200 µm
200 µm
z= -100 µm
z= -200 µm
z= -300 µm
z= -400 µm
z= -500 µm
z= -600 µm
B
z = +500 µm
z = +400 µm
z = +300 µm
z = 0 µm
z = +100 µm
z = +200 µm
10 µm
z = -100 µm
z = -200 µm
z = -300 µm
z = -400 µm
z = -500 µm
C
PSF x (µm)
x shift (µm)
D
PSF z (µm)
x shift (µm)
E
PSF x (µm)
z depth (µm)
F
PSF z (µm)
z depth (µm)
G
1 µm
x
FWHM =
0.405 ±
0.057
x (µm)
y
FWHM =
0.430 ±
0.030
y (µm)
z
FWHM =
2.351 ±
0.168
z (µm)

Figure S4, related to Figure 1. Characterization of the spatial resolution of the 3D DRIFT AO microscope.

(A) Maximal field of view of the 3D DRIFT AO microscope with the 20× objective at different AO z-focusing levels (from −600 µm to +500 µm). Images were taken at 880 nm. The fluorescent sample consisted of 6 µm-diameter beads on a cover glass. We moved the objective arm to compensate for the different AO z-focusing levels to keep the same beads in focus. Red numbers show the z shifts required for refocusing. The maximal field of view was ≈650 µm at z = 0 µm and beads remained visible in over a scanning range of 1100 µm. The z = 0 was the nominal focal plane of the objective.

(B) Image of a 6 µm diameter fluorescent bead at 880 nm. We moved the objective arm to compensate for the different AO z-focusing levels to keep the same bead in focus. Red numbers show the z shifts required for refocusing.

(C-F) Full width at half maximum values (mean ± SEM) of the point spread function along the z and x axes were plotted as a function of distance along the x and z axes.

(G) Spatial resolution in the center. Left, fluorescent image of a 170 nm fluorescence bead with 3D DRIFT AO microscope. Right, average normalized fluorescence histograms along the x, y and z axis for of n = 3 beads. Resolution was determined as the full width at half maximum in every histogram (mean ± SEM).

See also the *Characterization of the spatial resolution of the 3D DRIFT AO microscope* section in the **Supplemental Experimental Procedures.**

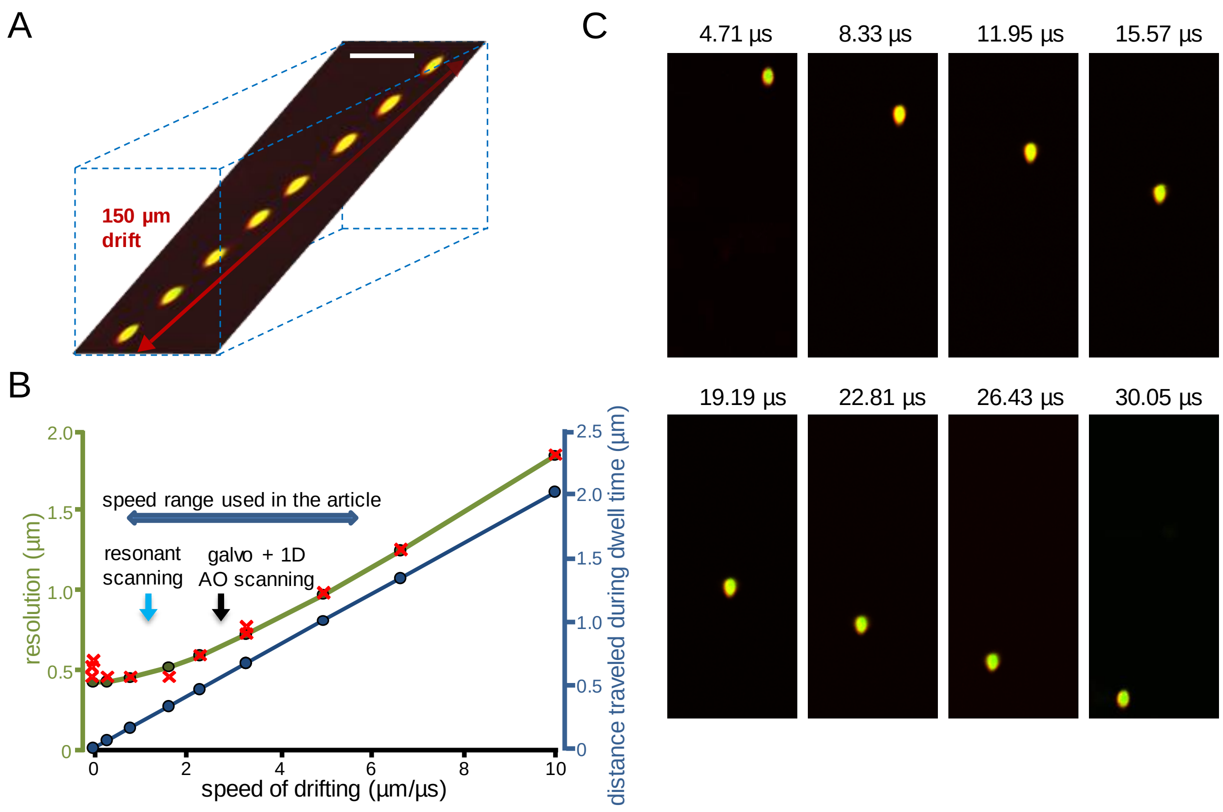

Figure S5, related to Figure 1. Effect of fast 3D AO drifts on the size of the PSF. 3D AO drifts were generated with up to 10 µm/µs speed in different directions to analyze elongation of the PSF.

(A) Maximal intensity projection image of a 6 µm fluorescent bead during 3D DRIFT AO scanning. Images of the fluorescent beads at different time points were overlaid. The 3D AO drift was generated with 4.55 µm/µs speed.

(B) FWHM of the PSF along the direction of the fast drift as a function of the speed of 3D drifts (green line). Note the increasing PSF at higher speeds. FWHM along the perpendicular direction did not change. This blurring effect of the PSF can be explained by the movement within the pixel dwell time (blue line). The blue arrow indicates the maximal scanning speed of the typical resonant galvo system (see for example Varga et al., 2011). The black arrow indicates the scanning speed of a typical 2D system with one integrated AO deflector (see for example Chen et al., 2012).

(C) Maximal intensity projection image of a 6 µm fluorescent bead with 3D DRIFT AO scanning at different time points.

For further limitations of see the *Limitations of 3D DRIFT AO microscopy* section in the **Supplemental Experimental Procedures.**

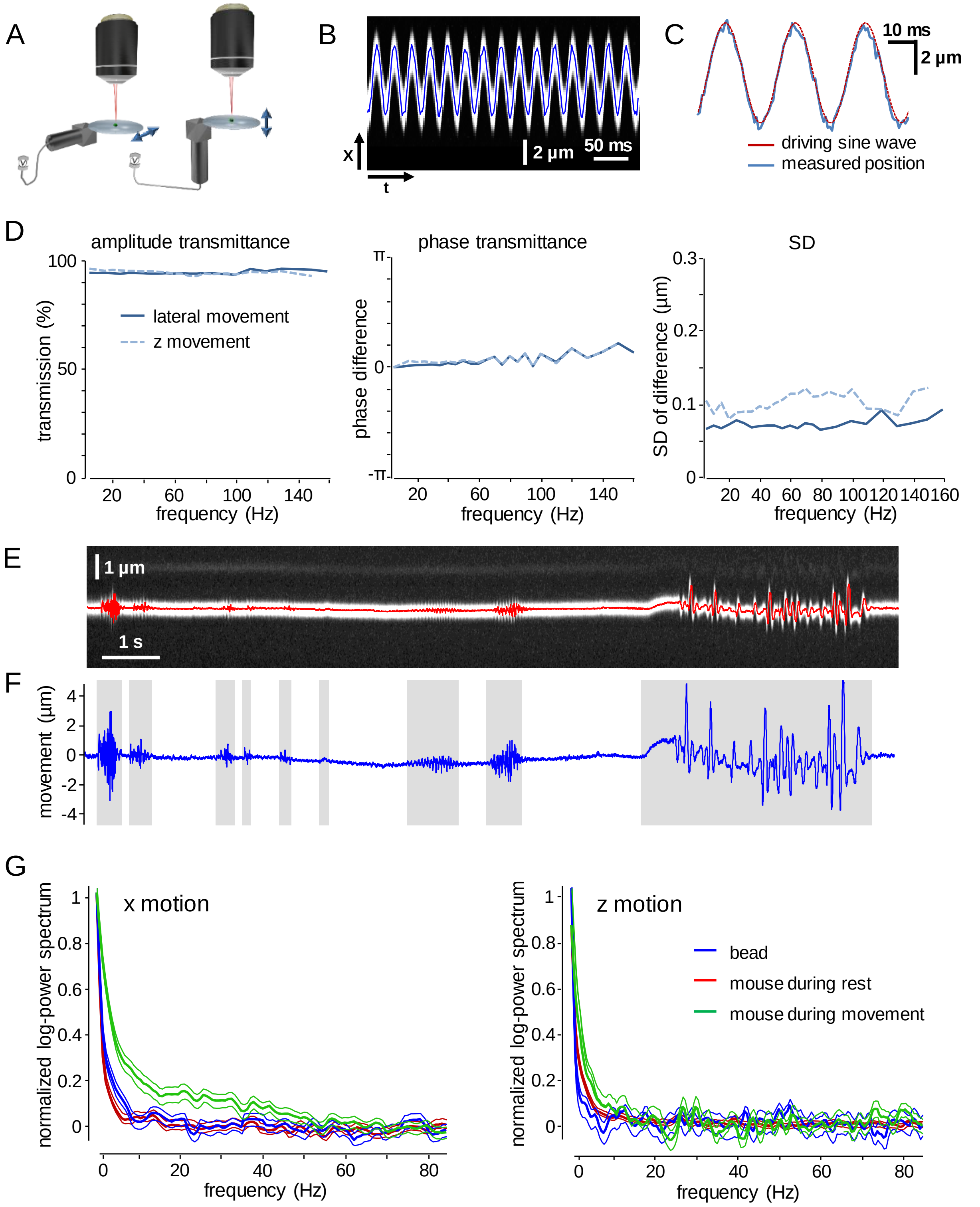

Figure S6, related to Figure 2. Validation of the fast 3D motion-detection method and measuring brain movement.

(A) The amplitude and phase transfer function of the fast 3D motion-detection method was measured in the 0-160 Hz bandwidth by using fluorescent beads and a fast piezo actuator. Fluorescent beads (diameter 6 µm) were fixed through a small cover glass onto the fast piezo actuator, which had a high resonant frequency (up to 2 kHz). This provided smooth mechanical

transmission in the 0-160 Hz frequency range. The piezo actuator oscillated either along the horizontal or the perpendicular directions by being driven with sine waves of 0-160 Hz frequency. The movement of a selected bead was detected by recording the position of its fluorescence using the fast 3D motion-detection method along the xy and xz planes.

(B) Example, fluorescence of the bead oscillating (at 40 Hz) along the x axis was projected to the x axis and plotted as a function of time and distance. The blue trace is the position of the maximum fluorescence, determined by Gaussian fitting for each time point.

(C) Maximal fluorescence was plotted as a function of time and distance and was fitted with a sine wave to measure amplitude transmittance and phase shift relative to the driver signal.

(D) Amplitude transmittance and phase shifts plotted against the frequency showed only modest changes in the 0-160 Hz range. The SD of the difference between the recorded bead position and the fitted sine wave was also less than 0.12 μm in the entire range for both axis movements. These data indicate that the transfer function of the fast 3D motion-detection allows measurement of brain movement with high spatial resolution in the 0-160 Hz range.

(E-G) The fast 3D motion-detection method was used to detect fast brain motion in awake mice.

(E) Position of a bright compact fluorescent object in the brain was recorded with the fast 3D motion-detection method at 160 Hz and plotted as a function of time. The red trace is the location of the maximum fluorescence determined by Gaussian fitting.

(F) Locomotion was simultaneously detected in the one dimensional virtual reality (see **Supplemental Experimental Procedures**) using an optical mouse. Gray bands indicate periods of motion.

(G) Fourier spectra of the movement of bright fluorescent objects were averaged (mean ± SEM) during rest (*red*) and running (*green*) periods (n=17 measurements) and were compared to the motion spectrum of fluorescent beads which were fixed to the animal holder (*blue*). Note that the spectral amplitude of brain movement was not significantly larger above 40-50 Hz from the spectral amplitude of the movement of fixed fluorescent beads, indicating that the 0-160 Hz bandwidth of the fast 3D motion-detection method is sufficient. As the SD of the movement of the fixed fluorescent beads was also small (0.075 ± 0.031 μm), these data indicate that the fast 3D motion-detection method can reliable capture *in vivo* brain movement.

See also the *Validation of the fast 3D motion-detection method and recording brain movement* section in the **Supplemental Experimental Procedures.**

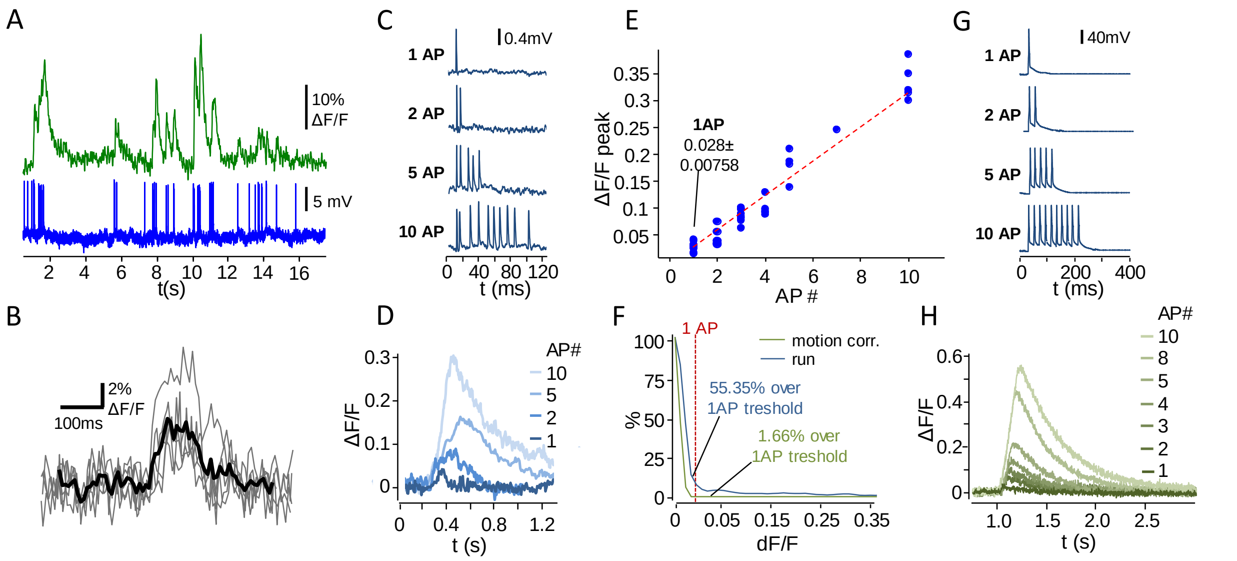

Figure S7, related to Figure 2. Simultaneous electrophysiological recording and 3D DRIFT AO imaging of APs.

(A) Ca^{2+} responses were recorded with chessboard scanning in an anesthetized mouse. Exemplified Ca^{2+} response (*green*) from the neuron recorded simultaneously with cell-attached pipette (voltage signal: *blue*).

(B) Individual single AP-induced Ca^{2+} responses (*gray*) and average of five responses (*black*).

(C) Exemplified cell-attached voltage signals with 1,2,5 and 10 APs recorded during *in vivo* conditions.

(D) Corresponding somatic Ca^{2+} responses.

(E) Amplitude of the somatic Ca^{2+} response as a function of the number of APs.

(F) Amplitude histogram of the motion-induced fluorescence intensity transients during running before (blue) and after motion correction (green). Average amplitude of single AP associated Ca^{2+} responses are indicated by red dashed line.

(G) Exemplified whole-cell voltage signals with 1,2,5, and 10 APs form an in vitro recording.

(H) Corresponding somatic Ca^{2+} responses.

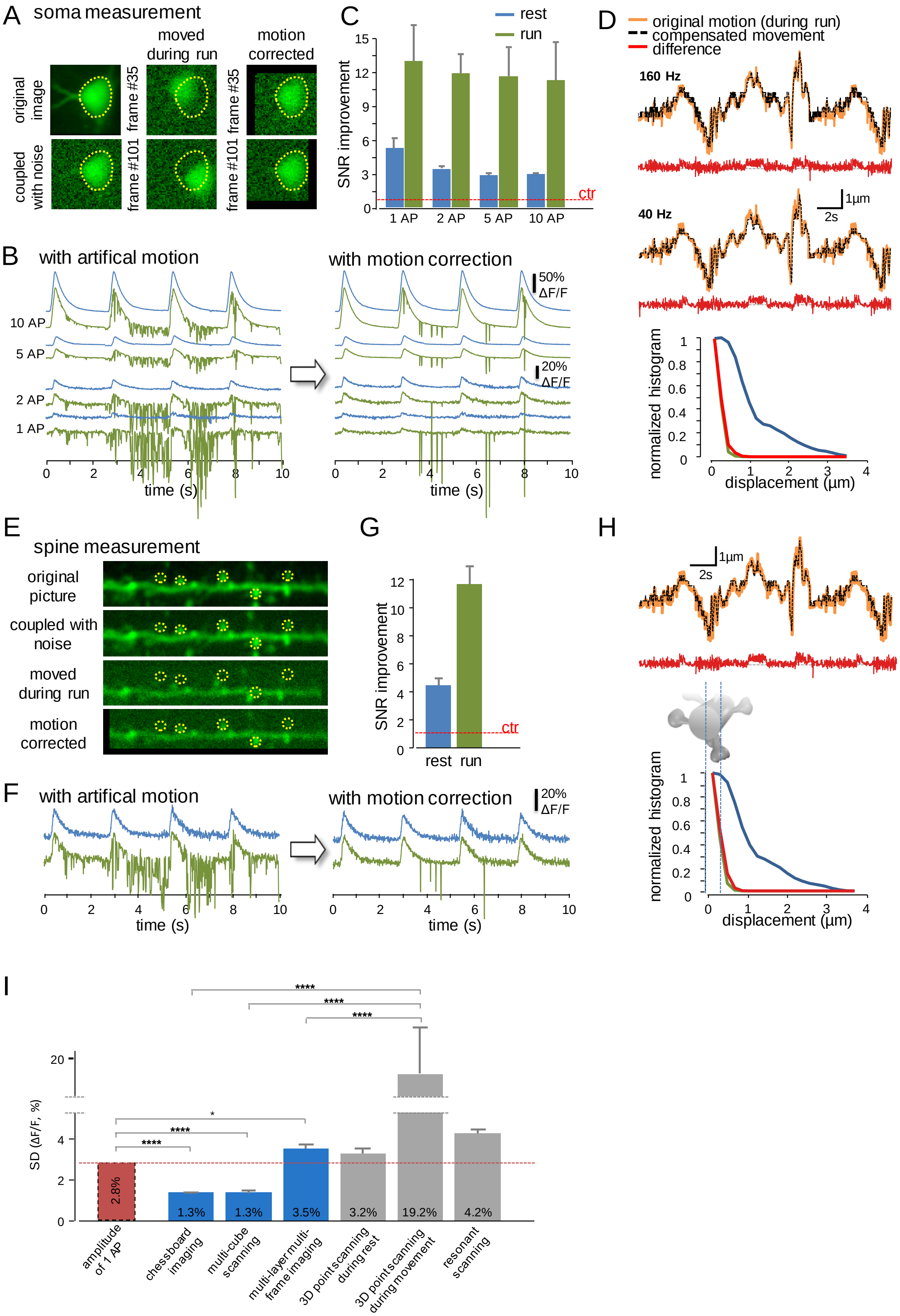

Figure S8, related to Figure 2. Estimating SNR improvement following the elimination of motion artifacts in multi-layer, multi-frame imaging, chessboard scanning, and ribbon scanning methods.

(A) Top left, smoothed image of an exemplified neuronal soma (*original image*). Bottom left, SD of the raw fluorescent pixel values was measured and averaged for multiple regions with no somatic or dendritic structure from the original raw videos, then white noise with the same SD (485.26 ± 88.66 DAC unit; n = 10) was added to each frame of the simulated video.

Middle column, displacement by brain movement was measured in advance as a function of time (see **Figure 2A**) using the fast 3D motion-detection method (at 160 Hz) and also added to the soma images at every time point to generate a time-lapse movie. In this way, we generated artificial videos for a more quantitative analysis. The two images are two example frames from the movie with different displacement amplitudes (*moved during run*). Yellow dotted lines indicate the initial location of the soma.

Right column, the same two example frames following the elimination of motion artifacts (*motion corrected*).

(B) Left, time-lapse movies were generated as in A, adding white noise, and brain displacement measured in advance to the original image, but here we also added ten, five, two, and one AP-generated responses and calculated somatic Ca^{2+} transients (*green traces*) from the somatic ROI. Control transients (*blue traces*) were generated without adding the brain displacement to the frames of the video. Right, the same ten, five, two, and one AP-generated responses, but transients were generated following motion artifact elimination. Note that the manual detection of one or two AP-induced transients is virtually impossible without the elimination of motion artifacts.

(C) Averaged improvement in SNR of Ca^{2+} transients following the elimination of motion artifacts was plotted as a function of the number of APs for resting (*blue*) and running (*green*) periods (mean ± SEM). Red dashed (*ctr.*) line indicates the SNR of Ca^{2+} transients without compensation.

(D) Top, exemplified transients. The original brain motion trajectory (*orange*) recorded with the fast 3D motion-detection method (160 Hz) was overlaid with the movement derived from the images (at 160 Hz imaging rate) during the off-line 3D motion-correction method (*black dashed line*). The difference between the two trajectories shows the error of the 3D motion-correction method (*red*). Note the small error (0.139 µm ± 0.106 µm) following motion artifact compensation. Middle, the same, but imaging rate was decreased to 40 Hz. As the 40 Hz imaging speed is about at the cut-off frequency of brain movement the reduced imaging speed did not significantly increased the error of the method. Bottom, normalized amplitude histogram of the motion during resting (*green*), and before (*blue*) and after (*red*) motion artifact correction during running periods.

(E-G) Similar to panels A-B, but for dendritic measurements. Calculations were made on dendritic spine responses (*yellow circles*). For these calculations we used the average spontaneous Ca^{2+} responses of dendritic spines, recorded in advance, instead of 1, 2, 5, and 10 AP-induced responses.

(H) Same as D but for dendrites. Note that the error following motion artefact compensation (*red*) is smaller than the average diameter of dendrites and spines. The average amplitude of the residual displacement error was small (0.118 ± 0.095 µm). The sum of the FWHM of the PSF (≈0.4 µm) and the average diameter of dendrites or spines (dendrite: 0.5-1.75 µm; spine: 0.42 ± 0.1 µm; see Holthoff et al., 2002, Konur et al., 2003) were larger than this average residual error, indicating that the special precision of motion correction is proper for dendritic and spine measurements.

(I) Multi-layer, multi-frame imaging, chessboard scanning, and multi-cube imaging have better single AP resolution in behaving animals than 3D random-access AO scanning or raster scanning. In this figure we compare the single AP resolution of six different scanning methods (multi-layer, multi-frame imaging, chessboard scanning, multi-cube imaging, 3D random-access scanning, and raster scanning). Neurons in the V1 region of the brain were labeled with GCaMP6f sensor using AAV vector and recorded at 880 nm. Standard deviation of individual Ca^{2+} transients recorded with the different scanning methods is compared to the average amplitude of single APs (see also **Figure S7** for AP amplitude) in the moving brain of behaving mice. Simultaneous scanning of over 100 neurons using chessboard scanning generated Ca^{2+} transients with a much smaller standard deviation than the average amplitude of single APs, indicating an excellent single AP resolution in behaving animals. Similarly, multi-cube scanning also has good single AP resolution. The standard deviation of Ca^{2+} transients, generated by multi-layer, multi-frame imaging, was just at the limit of single AP resolution. However, raster scanning or 3D random-access AO scanning in behaving animals cannot provide single AP resolution with the currently available GCaMP6f labeling. Although precise comparison of 2D and 3D scanning methods is not possible, we can conclude that the three new scanning strategies developed for somatic measurements provide much better signal-to-noise ratio, hence better single AP resolution, than previously used methods. P values are from left to right: 0, 1.04×10^{-10}, 2.24×10^{-1}, 3.02×10^{-14}, 6.01×10^{-11}, 3.15×10^{-9}.

See also the *Improvement in sensitivity of single AP* section in the **Supplemental Experimental Procedures.**

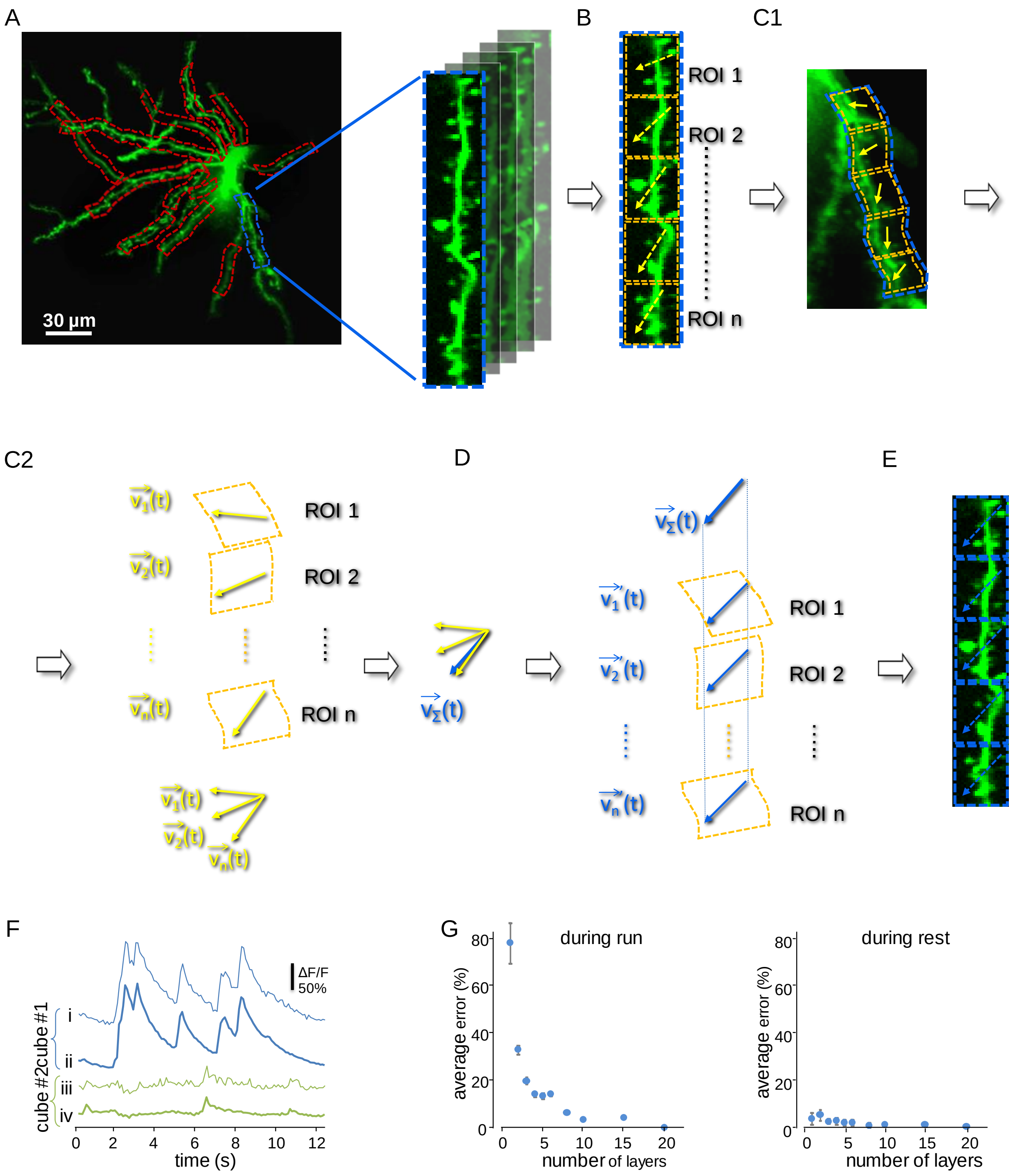

Figure S9, related to Figure 3. Motion correction in 3D.

(A-E) Schematic of 3D motion correction.

(A) The results of scanned ribbons are stored in time series of 2D frames in each dendritic region (dashed red boxes).

(B) The 2D frames of each ribbon are further sectioned to segments (ROIs) which are oriented parallel to the direction of the local AO drifts. Displacement vectors (dashed yellow arrows) are calculated separately for each segment as a function of time with autocorrelation.

(C1-C2) The displacement vectors are transformed to the original 3D Cartesian coordinate system of the sample (yellow arrows).

(D) The net displacement vector of a frame is taken as the median of the local displacement vectors of its ROIs, than this net displacement vector is transformed back to the 2D data array of segments.

(E) Finally, data were shifted in each segment with subpixel precision by the projection of the median displacement vector (blue dashed arrows).

(F-G) The efficiency of motion correction during multi-cube volume scanning.

(F) Activity of 10 neurons was recorded with multi-cube scanning. Each cube had a 30 µm × 30 µm base with 30 × 30 pixels resolution and the z dimension of each cube (the number of z layers used) was varied during the 3D measurements from 1 to 20 (z layers were evenly distributed in the total of 40 µm z range) to compare SNR of the 3D measurements and motion artefact compensation as a function of the z layers. (A) Exemplified transients were derived from recording cubes consisting of one (i and iii) and twenty (ii and iv) z layers. Note the improvement in the SNR when twenty layers were used.

(G) Ca^{2+} transients derived from recording cubes consisting of variable numbers of z layers (from 1 to 20) were subtracted from the Ca^{2+} transient recorded simultaneously with a cube of twenty layers. Then the absolute value of the relative error was averaged over time during running and rest and is shown on the y axes as mean ± SD.

See the *Motion correction in 3D* section in the **Supplemental Experimental Procedures.**

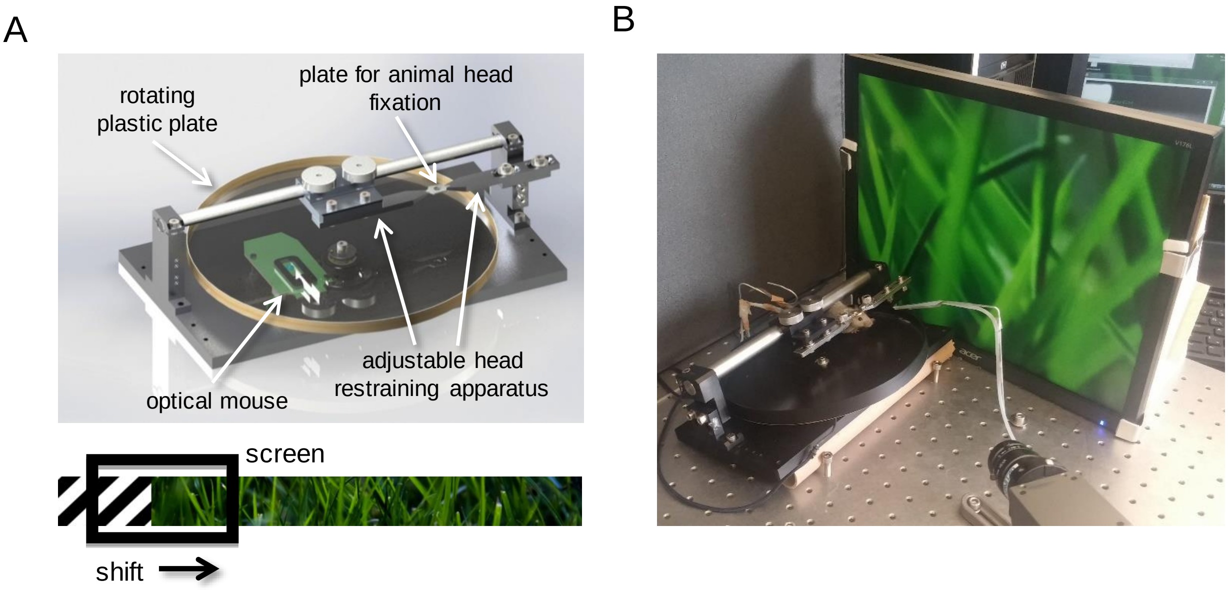

Figure S10, Experimental Procedures. Virtual reality (VR). A linear treadmill was used to record the velocity of movement during fluorescent recording. (A) Solidworks image of the virtual reality environment. The position of a rotating light plastic plate was decoded by an optical mouse mounted upside down, and loaded into the MATLAB-based program. A 10 mm steel rod was stretched at both sides to minimize movement of the head-restraining apparatus during motion. A video screen placed in front of the left eye of the mouse was continuously updated according to the position signal with the actual part of an image. We realized a linear maze by teleporting the animal to the beginning of the VR image when it reached the end. (B) Image of the VR used with the 3D DRIFT AO microscope.

See the Virtual reality environment section in the **Supplemental Experimental Procedures.**

Note: Non-linear chirps in the x axis deflectors are defined as: $f_i(x,t) = f_i(0,0) + (b_{xi} * (t - \frac{D}{2*v_a} - \frac{x}{v_a}) + c_{xi}) * \left(t - \frac{D}{2*v_a} - \frac{x}{v_a}\right)$

where i=1 or 2 indicating the first and second x deflector, respectively. See Equation S1. A similar equation is required for the y axis deflectors

In this table we determine the parameters of the non-linear chirps in the four AO deflectors (x_1, x_2, y_1, and y_2) to generate 3D drifts in any arbitrary direction and at any desired speed (v_{x0}, v_{y0}, v_{zx0} (= v_{zy0})) from a given starting point ($x_0(0)$, $y_0(0)$, $z_0(0)$)

Chirp parameters required to be expressed	Chirp parameters expressed as a function of the $x_0(0)$, $y_0(0)$, $z_0(0)$, v_{x0}, v_{y0}, and v_{zx0} (= v_{zy0}) parameters of the focal spot
Δf_{0x} (= $f_{1x}(0,0)$ - $f_{2x}(0,0)$)	$$\Delta f_{0x} = \frac{x_0(0) * F_2}{K * F_{obj} * F_1}$$
b_{x1}	$$b_{x1} = \frac{v_{zx0} * v_a}{4 * K} * \left(\frac{M + \frac{v_{zx0}}{v_{x0}} * \frac{x_0(0) * F_2}{F_{obj} * F_1}}{z_0(0) - \frac{v_{zx0}}{v_{x0}} * x_0(0)} \right)^2$$
b_{x2}	$$b_{x2} = \frac{v_{zx0} * v_a}{4 * K} * \left(\frac{M + \frac{v_{zx0}}{v_{x0}} * \frac{x_0(0) * F_2}{F_{obj} * F_1}}{z_0(0) - \frac{v_{zx0}}{v_{x0}} * x_0(0)} \right)^2$$
c_{x1}	$$c_{x1} = \frac{M * v_a}{2 * K} * \left(\frac{M + \frac{v_{zx0}}{v_{x0}} * \frac{x_0(0) * F_2}{F_{obj} * F_1}}{z_0(0) - \frac{v_{zx0}}{v_{x0}} * x_0(0)} - \frac{M}{F_{obj}} \right) + \frac{v_{x0}}{2 * K * M} * (z_0(0) - \frac{v_{zx0}}{v_{x0}} * x_0(0)\ ) * \left(\frac{M + \frac{v_{zx0}}{v_{x0}} * \frac{x_0(0) * F_2}{F_{obj} * F_1}}{z_0(0) - \frac{v_{zx0}}{v_{x0}} * x_0(0)} \right)^2$$

c_{x2}	$c_{x2} = \frac{M * v_a}{2 * K} * \left(\frac{M + \frac{v_{zx0}}{v_{x0}} * \frac{x_0(0) * F_2}{F_{obj} * F_1}}{z_0(0) - \frac{v_{zx0}}{v_{x0}} * x_0(0)} - \frac{M}{F_{obj}} \right) - \frac{v_{x0} * (z_0(0) - \frac{v_{zx0}}{v_{x0}} * x_0(0))}{2 * K * M} * \left(\frac{M + \frac{v_{zx0}}{v_{x0}} * \frac{x_0(0) * F_2}{F_{obj} * F_1}}{z_0(0) - \frac{v_{zx0}}{v_{x0}} * x_0(0)} \right)^2$
Δf_{0y} (= $f_{1y}(0,0)$ - $f_{2y}(0,0)$)	$\Delta f_{0y} = \frac{y_0(0) * F_2}{K * F_{obj} * F_1}$
b_{y1}	$b_{y1} = \frac{v_{zy0} * v_a}{4 * K} * \left(\frac{M + \frac{v_{zy0}}{v_{y0}} * \frac{y_0(0) * F_2}{F_{obj} * F_1}}{z_0(0) - \frac{v_{zy0}}{v_{y0}} * y_0(0)} \right)^2$
b_{y2}	$b_{y2} = \frac{v_{zy0} * v_a}{4 * K} * \left(\frac{M + \frac{v_{zy0}}{v_{y0}} * \frac{y_0(0) * F_2}{F_{obj} * F_1}}{z_0(0) - \frac{v_{zy0}}{v_{y0}} * y_0(0)} \right)^2$
c_{y1}	$c_{y1} = \frac{M * v_a}{2 * K} * \left(\frac{M + \frac{v_{zy0}}{v_{y0}} * \frac{y_0(0) * F_2}{F_{obj} * F_1}}{z_0(0) - \frac{v_{zy0}}{v_{y0}} * y_0(0)} - \frac{M}{f_{obj}} \right) + \frac{v_{y0} * (z_0(0) - \frac{v_{zy0}}{v_{y0}} * y_0(0))}{2 * K * M} * \left(\frac{M + \frac{v_{zy0}}{v_{y0}} * \frac{y_0(0) * F_2}{F_{obj} * F_1}}{z_0(0) - \frac{v_{zy0}}{v_{y0}} * y_0(0)} \right)^2$

c_{y2}	$$c_{y2} = \frac{M * v_a}{2 * K} * \left(\frac{M + \frac{v_{zy0}}{v_{y0}} * \frac{y_0(0) * F_2}{F_{obj} * F_1}}{z_0(0) - \frac{v_{zy0}}{v_{y0}} * y_0(0)} - \frac{M}{f_{obj}} \right) - \frac{v_{y0} * (z_0(0) - \frac{v_{zy0}}{v_{y0}} * y_0(0))}{2 * K * M} * \left(\frac{M + \frac{v_{zy0}}{v_{y0}} * \frac{y_0(0) * F_2}{F_{obj} * F_1}}{z_0(0) - \frac{v_{zy0}}{v_{y0}} * y_0(0)} \right)^2$$

Supplemental Table 1, related to Experimental Procedures. Parameters of the non-linear chirps in the four AO deflectors. The parameters required for 3D DRIFT AO scanning in the four AO deflectors: Δf_{0x}, b_{x1}, b_{x2}, c_{x1}, c_{x2}, Δf_{0y}, b_{y1}, b_{y2}, c_{y1}, and c_{y2} are expressed as a function of the initial location ($x_0(0)$, $y_0(0)$, $z_0(0)$) and vector speed (v_{x0}, v_{y0},$v_{zx0} = v_{zy0}$) of the focal spot.

wavelength	800 nm	875 nm	900 nm	925 nm	950 nm
	800 nm	875 nm	900 nm	925 nm	950 nm
GDD (fs^2)	14342	12663	12194	11660	11267
TOD (fs^3)	8543	7752	7545	7340	7153

Supplemental Table 2, related to Figure 1. Second- (GDD) and third-order temporal dispersion (TOD) of the AO deflectors as a function of wavelength.

Supplemental Movie Legends

Movie S1, related to Figure 1. Validation of 3D DRIFT AO scanning by bleaching a polyhedron shape in a homogeneous fluorescent sample.

Resolution and point stability of the 3D DRIFT AO scanning method was validated by “burning” a polyhedron shape in a homogeneous fluorescent sample using photo bleaching. Each edge of the polyhedron was scanned by a 3D line which was generated within a single AO-switch time period (33 µs). The precision and reproducibility of the 3D scanning was verified by a z-stack performed at a much lower intensity. The movie shows the inverted fluorescence intensity of the z-stack. Note the stability of the 3D lines during scanning.

Movie S2, related to Figure 1. Large AO 3D scanning volume using GECIs.

Z-stack from 650 µm to 100 µm under the pia mater in the visual cortex of a Thy1-Cre mouse was taken using the 3D DRIFT AO microscope. For cre-dependent expression of the GCaMP6f Ca^{2+} sensor we used an AAV vector injected into the V1 region. Raw fluorescence data were normalized for radially inhomogeneous illumination as previously described (Katona et al., 2012).

Movie S3, related to Figure 1. Method of selection of 3D ribbons for fast scanning of spiny dendritic segments.

The video demonstrates how guiding points are selected, how the 3D trajectory is fitted according to the guiding points, and, finally, how the plan of the ribbon is generated for 3D ribbon scanning. See also the *Selection of the 3D trajectory and the ribbons for 3D ribbon and snake scanning* section in the in the **Supplemental Experimental Procedures.**

Movie S4, related to Figure 1. 3D ribbon scanning of a spiny dendritic segment.

A 140 µm GCaMP6-labeled spiny dendritic segment situated in a 60 µm range of z scanning was imaged using 3D ribbon scanning.

Movie S5, related to Figure 1. 3D ribbon scanning of a spiny dendritic segment during visual stimulation.

Similar example as **Movie S4**. A GCaMP6-labeled spiny dendritic segment from a different layer II/III neuron was imaged using 3D ribbon scanning.

Movie S6, related to Figures 2. and 3. Multi-3D ribbon scanning; raw data recorded in behaving animal.

Twelve spiny dendritic segments of a GCaMP6-labeled layer II/III pyramidal cell located in V1 was simultaneously imaged using 3D ribbon scanning. Note the large and spatially inhomogeneous movement artifacts during recording of activity in an awake animal.

Movie S7, related to Figure 3. Multi-3D ribbon scanning movie before and after the correction of motion artifacts.

The first block is the same movie as **Movie S5. In the second block of the movie,** motion artifacts were eliminated by shifting back each frame of all regions by the local projection of the net displacement vector of the brain (see text for details). This correction was made with subpixel resolution. Note the small residual motion in the video. Finally, in the last block of the movie residual motion, artifacts were removed by repeating the cross-correlations-based displacement calculations and the consequent back shifts for each frame separately on all dendritic segments.

Movie S8, related to Figures 2-4. Representative example of cell selection and image acquisition during chessboard scanning in 3D.

Populations of V1 neurons were labeled with GCaMP6f protein expressed by non-specific promoter. First, a z-stack was obtained using the 3D AO microscope. Second, ROIs, here small squares centered on neuronal somata, were selected. Finally, the measured ROIs were arranged into the form of a chessboard. In this way, 3D activity was visualized during data acquisition, and then stored as a 2D movie.

Movie S9, related to Figure 4. Three-dimensional recording neuronal networks in V1 with chessboard scanning during visual stimulation.

The same populations of V1 neurons as in **Movie S8**. Activity was recorded in awake, head-restrained animals during visual stimulation. The upper left corner shows the video of raw fluorescence data arranged in chessboard form. The upper right corner shows the motion-corrected video converted to relative fluorescence changes ($\Delta F/F$). In the lower left corner the actual visual stimulation is presented, while in the lower right corner the transients of nine exemplified neurons are showed during the actual visual stimulation.

Movie S10, related to Figure 5. Multi-layer multi-frame imaging of neuropil activity with over 100 Hz temporal resolution.

V1 neurons were sparsely labeled using the GCaMP6f sensor and measured simultaneously in four different z planes (z_1= -380 µm, z_2= -350 µm, z_3= -295 µm, z_4= -130 µm) with 101 Hz temporal resolution during visual stimulation with moving gratings. Movies were converted to relative fluorescence changes (ΔF/F) and aligned next to each other.

Movie S11, related to Figure 6. Snake scanning of a spiny dendritic segment in the moving brain of a behaving mouse.

V1 neurons were sparsely labeled using the GCaMP6f sensor. A spiny dendritic segment was selected for volume imaging using snake scanning at 10 Hz. Motion artifacts were removed and data are shown as relative fluorescence changes (ΔF/F) overlaid on the averaged baseline fluorescence.

Movie S12, related to Figure 6. Multi-cube imaging of neuronal activity.

V1 neurons were sparsely labeled using the GCaMP6f sensor. Ten labeled neurons were centered in cubes. Volume imaging was performed simultaneously in the ten cubes with 10 Hz temporal resolution. Finally, motion artifacts were removed and data are shown as relative fluorescence changes (ΔF/F). Note the fine Ca^{2+} dynamic in the somatic and proximal dendritic segments.

Movie S13, related to Experimental Procedures. Z-stack of a GCaMP6f-labeled neuronal population.

Z-stack from 0 µm to 820 µm under the pia mater in the visual cortex of a Thy1-Cre mouse was taken using 2D galvo scanning. Detectors were fixed to the objective arm (travelling detector system) to minimize the detection pathway and maximize detection of backscattered fluorescent light. For cre-dependent expression of the GCaMP6f Ca^{2+} sensor we used an AAV vector injected into the V1 region. See **Supplemental Experimental Procedures.**

Supplemental Files

Supplemental File 1, related to Experimental Procedures. Verilog code of the signal generation.
Signal generation and high speed communication with the DAC chip performed by the FPGA (see also **Figure S3**).

Supplemental File 2, related to Experimental Procedures. MATLAB function calculating AO driving signal.
This MATLAB function returns the four center frequencies in MHz, the four frequency step speeds in MHz/s, and the four frequency derivate step values in MHz/s^2 for any desired point in the field of view with any desired speed vector.

Supplemental File 3, related to Experimental Procedures. MATLAB function calculating register values for the FPGA.
This MATLAB function returns the register values transferred to the FPGA for synthesis. Center frequencies, frequency steps, and frequency derivate steps calculated for each scanning segment are the inputs, while register values FR, FSR, FDSR are the output.

SUPPLEMENTAL EXPERIMENTAL PROCEDURES

(related to Experimental Procedures)

Mice

All experimental protocols were approved by the Animal Care and Experimentation Committee of the Institute of Experimental Medicine of the Hungarian Academy of Sciences (approval reference numbers PEI/001/194-4/2014 and PEI/001/1771-2/2015). All procedures complied with Hungarian and European regulations for animal research, as well as with the guidelines of Society for Neuroscience. The recordings from the primary visual cortex were performed on C57Bl/6J (RRID:IMSR_JAX:000664), Thy-1-Cre (RRID: IMSR_JAX:006143), and VIP-Cre (RRID: IMSR_JAX:010908) mice (P60-120). Animals were allowed free access to food and water and were maintained in temperature-, humidity-, and light-controlled conditions. For measurement of VIP neurons we used VIP-cre mice.

Surgical procedure

The surgical process was similar to that described previously (Katona et al., 2012), with some minor modifications, briefly: mice were anesthetized with a mixture of midazolam, fentanyl, and medetomidine (5 mg, 0.05 mg and 0.5 mg/kg body weight, respectively); the V1 region of the visual cortex was localized by intrinsic imaging (on average 0.5 mm anterior and 1.5 mm lateral to the lambda structure); a round craniotomy was made over the V1 using a dental drill, and was fully covered with a double cover glass, as described previously (Goldey et al., 2014). For two-photon recordings, mice were awakened from the fentanyl anesthesia with a mixture of nexodal, revetor, and flumazenil (1.2 mg, 2.5 mg, and 2.5 mg/kg body weight, respectively) and kept under calm and temperature-controlled conditions for 2-12 minutes before the experiment. Before the imaging sessions, the mice were kept head-restrained in the dark under the 3D microscope for at least 1 hour to accommodate to the setup. In some of the animals, a second or third imaging session was carried out after 24 or 48 hours, respectively.

AAV labeling

The V1 region was localized with intrinsic imaging, briefly: the skin was opened and the skull over the right hemisphere of the cortex was cleared. The intrinsic signal was recorded using the same visual stimulation protocol we used later during the two-photon imaging session. The injection procedure

was performed as described previously (Chen et al., 2013), with some modifications. A 0.5 mm hole was opened in the skull with the tip of a dental drill over the V1 cortical region (centered 1.5 mm lateral and 1.5 mm posterior to the bregma). The glass micro-pipette (tip diameter ≈10 µm) used for the injections was back-filled with 0.5 µl vector solution (≈6×10^{13} particles/ml) then injected slowly (20 nl/s for first 50 nl, and 2 nl/s for the remaining quantity) into the cortex, at a depth of 400 µm under the pia. For population imaging we used AAV9.Syn.GCaMP6s.WPRE.SV40 or AAV9.Syn.Flex.GCaMP6f.WPRE.SV40 (in the case of Thy-1-Cre and VIP-Cre animals); both viruses were from Penn Vector Core, Philadelphia, PA. For sparse labeling we diluted AAV1.hSyn.Cre.WPRE.hGH 10,000 times and injected in a mixture of AAV9.Syn.Flex.GCaMP6f.WPRE.SV40. The cranial window was implanted over the injection site 2 weeks after the injection, as described in the surgical procedure section.

***In vivo* and *in vitro* electrophysiology**

To determine the Ca^{2+} transient kinetics as a function of increasing AP number during an AP burst, first we tested the correlation *in vitro*. The mice (40 to 45 days old) were deeply anesthetized with isoflurane and then decapitated. The brain was quickly removed from the skull and put into an ice-cold cutting solution (Chiovini et al., 2010). Horizontal acute slices (thickness: 300 µm) were cut using a vibratome (Leica VT1000S). The slices were then stored at room temperature (23-25°C) in standard artificial cerebrospinal fluid containing (in mM): 126 NaCl, 2.5 KCl, 2 $CaCl_2$, 2 $MgCl_2$, 1.25 NaH_2PO_2, 26 $NaHCO_3$, and 10 glucose. It was then bubbled with carbogen gas (Rozsa et al., 2004, Chiovini et al., 2010). Pyramidal neurons were visualized for patch-clamp recordings using oblique infrared illumination and two-photon imaging (880 nm). Whole-cell current-clamp recording (MultiClamp 700B, Molecular Devices, Sunnyvale, CA, USA) was performed from cells labeled with the GCaMP6f Ca^{2+} sensor in mice that were injected at least 2 weeks before the experiment. Recordings were taken at 32-34°C and performed with 6-9 MΩ-resistance borosilicate glass electrodes filled with an intracellular solution containing (in mM): 125 K-gluconate, 20 KCl, 10 HEPES, 10 phosphocreatine di-tris-salt, 0.3 Na-GTP, 4 Mg-ATP, 10 NaCl. Data were recorded using Digidata 1440, pClamp10 (Molecular Devices, Sunnyvale, CA, USA), and MES (Femtonics Ltd., Budapest, Hungary) software. We evoked bursts of 1, 2, 3, 4, 5, 8, 10, and 14 APs using somatic current steps (500-700 pA for 5 ms) with 20 ms delay between the individual APs in the burst. Data were filtered at 3-10 kHz and recorded at 20 kHz. In some cases, a sine wave was fitted to the low-pass filtered raw traces and was then subtracted in order to remove the 50 Hz noise using MES (Femtonics Ltd., Budapest, Hungary). To determine the correlation between Ca^{2+} transient and AP number, we performed cell-attached patch-clamp recording in *in vivo* conditions. The surgery was as described above, except that a small

area, about 1 mm wide, next to the cover glasses was left uncovered: this region was used as the insertion site for the pipette, while the cells were measured under the cover glass, to limit tissue motion. For the recordings we used 7-8 MΩ-resistance borosilicate glass electrodes filled with extracellular solution, containing (in mM): 126 NaCl, 2.5 KCl, 2 $CaCl_2$, 2 $MgCl_2$, 1.25 NaH_2PO_2, and 100 µM Alexa 594 (Invitrogen) to visualize the pipette. Electrophysiological data were recorded in current-clamp mode with 0 mV holding potential simultaneously with the calcium imaging. Transients were band-pass filtered between 1 Hz and 5 kHz and the number of APs was counted manually.

Auditory discrimination task

As previously described (Pi et al., 2013), water-deprived mice were trained on an auditory discrimination task. Head-fixed and placed in an unlit chamber, they had to lick when they heard a go tone (5 kHz frequency, 0.5 s duration) to get a water reward (5 µl per trial) and not lick when they heard a no-go tone (10 kHz frequency, 0.5 s duration). The no-go tone was associated with an air puff (100 ms duration) as a punishment. To detect licking we used an infrared sensor (Island Motion Co.). Tones were generated by PulsePal (SanWorks). Acquisition of the behavioral data was performed by Bpod device (SanWorks).

Visual stimulation

During data acquisition, a visual stimulus was delivered in the form of a drifting grating as described previously (Katona et al., 2012). Briefly, each visual stimulation trial started by showing a gray screen; after 2 s, a grating appeared in the screen (spatial frequency: 0.25 cyc/°), then moved in a direction orthogonal to its orientation (for 5 s at 1cyc/s), stopped for 2 s, and finally disappeared, leaving the initial gray screen until the onset of the next stimulus. Trials with eight different grating directions were tested with an angular interval of 45°.

Virtual reality environment

During the awake experiments, the mouse was head fixed, but free to run on a custom-designed linear treadmill made of a light rotating plastic plate (**Figure S10**). Easy rotation of the plate was provided by two ball-bearings (619/8, SKF). The motion of the treadmill was recorded by a high speed (1 kHz) optical mouse (Urage reaper 3090, Hama) mounted upside down on the other side of the dial, and the angular position was recorded simultaneously at the same time as fluorescent signal. We used a self-developed script written in MATLAB to move the "wall" of the labyrinth on a

15” LCD screen according to the position signal of the optical mouse. Then we exported the output curves (velocity, position) to Excel for further analysis. Animals were habituated to the wheel for 20 minutes every second day before the experiment, but no other behavioral training was used. Traces were divided into temporal intervals, running and rest, which were separated by using a 2.6 mm/s speed threshold.

AO deflectors and drivers

The large aperture (15 mm × 15 mm) deflectors (*AO1-AO4,* **Figure S1A**) were custom manufactured from a TeO_2, uniaxial crystal (length: 29.7 mm) with high acousto-optic figure of merit (MD085, Gooch and Housego) for the 850-950 nm wavelength range with a peak bandwidth at 900 nm. The deflection configurations use the slow shear acoustic mode, propagating at small (3-10°) angles relative to the [1,1,0] crystallographic axis (Maák, 1999). The optimal propagation angle was set to 8.5° for 700 nm and 10° for 900 nm to maximize the acoustic frequency bandwidth at higher wavelengths. The propagation angles can be different for the deflectors of the z-focusing and 2D AO scanning units, but in both units the time-bandwidth products (=bandwidth × optical aperture / acoustic speed in AO deflectors) were around 1056 (=50 MHz × 15 mm / (710 m/s)) for full aperture illumination. The center acoustic frequency in both units was 80 MHz with, a 3 dB bandwidth of 50 MHz. The maximal angular deflection of a deflector was 143.5 mrad (8.21°) at 950 nm. The second- and third-order temporal dispersion (GDD and TOD) of one TeO_2 deflector varies in the 800-950 nm range from 14,342 to 11, 267 fs^2 and from 8543 to 7153 fs^3, respectively (**Table S2**). The maximal angular dispersion after the second pair of deflectors is 3.1 mrad, at the edges of the scanned region. This angular dispersion was reduced by the compensation unit to less than 1 mrad, as described earlier in Katona et al. 2012. The maximal deflection efficiency (ratio of the laser-power deflected into the first diffraction order) did not depend on crystal orientation (**Figure S2A**); however, deflector bandwidth does (**Figure S2B**). For example, deflectors developed for 900 nm (with a propagation angle of 10°) had a much broader bandwidth at longer wavelengths than those optimized for 700 nm (with a propagation angle of 8.5°) (**Figure S2B**). The new deflector’s transducers were also capable of supporting higher radio-frequency power, hence could generate more acoustic power (needed to compensate for the diffraction efficiency decrease at increased wavelengths).

The relatively high second- and third-order dispersion of the AO deflectors (**Table S2**) was within the range of the compensation capability of the motorized four-prism sequence (see the *3D microscope design* section in the **Supplemental Experimental Procedures**). We also optimized the electric circuitry, coupling the radio frequency driver to the piezo transducer, to increase the AO wave generation efficiency and bandwidth. The new matching circuit is based on an impedance

transformer that transforms the typically low impedance (5-20 Ohms) of the transducer to best approach the nominal 50 Ohms at the middle of the band. Capacitors and inductivities are added to correct for the mismatch on the sides of the band. The new circuit provides around 10-20% better energy transfer from the driver to the transducer than in former versions.

A limitation of random-access 3D point scanning is that the large optical aperture of AO deflectors must be filled by an acoustic wave to address a given scanning point which limits either the measurement speed or the number of ROIs. This is because to achieve large scanning volumes with a high spatial resolution, we need large AO deflector apertures. However, to fill these large apertures with an acoustic signal takes considerable time. Therefore, the long-duration AO switching time slows down the surface and volume scanning rate by approximately the ratio of the AO switching time and (optimal) pixel dwell time (about 0.1-1 μs) in case of point scanning. In contrast, pixel dwell time is limited only by the rate of PMT board and by the bandwidth of the PMT during 3D drifts.

3D microscope design

The improved microscope setup is constructed on the basis of the previous setup reported in our earlier work (see Supplementary Figures 1-8 and Supplementary Notes 1-8 in Katona et al., 2012). The 105 fs long laser pulses were delivered by a Ti:S laser at a 80 MHz repetition rate (Mai Tai, Spectra Physics, **Figure S1**). The coherent backreflections from optical elements were blocked by a new type of Faraday isolator (BB9-5I, EOT) that had a higher center wavelength (900 nm) and provided 5-10% higher throughput in the desired (850-950 nm) wavelength range (92% transmission at 900 nm) than the previous version (Katona et al., 2012). The elimination of thermal drift errors were realized by two automated beam stabilization units (*beam stab. #1* and *#2*). Each stabilization unit was built from two position sensors (quadrant detectors, *q1-q2, and q3-q4*) and two motorized mirrors (*m1-m2*, and *m9-m10*), wired in a feedback loop. The first beam stabilization unit contained the following items: *m1, m2, m3, m4, m5, q1*, and *q2* (see **Figure S1**), where *m1* and *m2* are silver mirrors (PF03-03-P01, Thorlabs) in piezoelectric mounts (ASM003, Thorlabs) controlled by a high voltage electronic controller (TPZ001, Thorlabs) and *m3* and *m4* are backside polished broadband dielectric mirrors (BB1-E03P, Thorlabs). The position of the laser beam is detected by quadrant detectors (*q1* and *q2*, PDQ80A and TPA101, Thorlabs). All the electronics are mounted in a control hub (TCH002, Thorlabs) and programmed to perform closed-loop beam alignment at high speed (≈300 Hz).

The temporal dispersion introduced by the acousto-optic devices and other optical elements in the assembly is compensated by the introduction of a new, motorized four-prism compressor that optimizes the dispersion compensation parallel with the wavelength tuning (**Figure 1B**). The four prism sequence (*P1-P4*) is coupled to the light pathway by two broadband dielectric mirrors *m6* and *m9* (BB1-E03, Thorlabs), and consists of Brewster angle prisms from SF11 (320-8525 apex angle 59°, Eksma Optics; **Figure S1A, B**). The apex distance between *P2* and *P3* was 692 mm at 880 nm (**Figure 1B**). The retro reflector after the fourth prism (*m7* and *m8*) was made of two square dielectric broadband mirrors (BBSQ05-E03, Thorlabs). To obtain the same level of second-order dispersion compensation, the distance between the first and second prism pairs should vary linearly with wavelength. Moreover, the angle of refraction at the first prism pair changes with wavelength, so the second pair should be shifted in the plane of refraction to meet the refracted beam at the optimal position of its aperture. Therefore, the first group of prisms (*P1* and *P2*) was fixed, and the second group (*P3* and *P4*) and the two retro mirrors (*m7* and *m8*) were translated in the horizontal plane using two motorized linear slides with orthogonal axes (**Figure 1B**). Positions could be set automatically at each target wavelength. This combination added a large negative second- (up to 75-80,000 fs^2) and third-order (up to 35-40,000 fs^3) dispersion that can be tuned with the wavelength. Moreover, we were able to skip the dispersion compensation unit (Deep See Unit, Spectra Physics) of the Mai Tai laser, which also increased total transmission by about 6% and simplified the system. The amount of dispersion compensation required wasn't a monotonic function of wavelength, meaning that the distances between the first and second group of prisms need to be varied in a range between 650 mm and 970 mm in the desired 800-950 nm wavelength range in order to get the maximal two-photon excitation at the depth. The fine-tuning of the prisms was performed by optimizing the positions of the two motorized slides based on two-photon image contrast and SNR at each wavelength.

The second stabilization unit is placed after the prism compressor and contains the following items: *m9, m10, m11, m12, m13, q3,* and *q4*. The *m9* and *m10* broadband dielectric mirrors were mounted on motorized mirror mounts (U100-AC and NSA12, Newport) and driven by Newport servo motor drivers (NSC200 and NSC-SB). The same types of quadrant detectors (q3 and q4) were used (PDQ80A and TPA101, Thorlabs), as in the first beam stabilization unit. The beam alignment was performed by the LaserControl software (Femtonics).

The beam expander consists of two achromatic lenses *L1* (ACN254-075-B, Thorlabs) and *L2* (AC508-2000-B, Thorlabs) and is connected to the optical pathway broadband dielectric mirrors (*m13, m14, m15,* and *m16*; BB1-E03 Thorlabs). *L1* and *L2* lenses were selected to set the laser beam

to match the large apertures (15 mm) of the first pair of AO deflectors. Some mirrors are omitted on the block diagram to make the beam path more manageable, these mirrors are 1 inch dielectric (BB1-E03, Thorlabs) before the beam expander, and 2 inch dielectric mirrors (BB2-E03, Thorlabs) after the beam expander.

The first two deflectors (*AO1* and *AO2*) formed two orthogonal electric cylinder lenses (*AO z-focusing unit*) and were filled with chirped acoustic waves whose frequencies were changed according to the protocol described in the **Supplemental Experimental Procedures (Equations S1-S70,** summarized in **Table S1)**. A new adjustment technique for the acousto-optical deflectors, a mechanical actuator with six degrees of mechanical freedom (instead of the former version with five degrees of freedom) meant it was possible to carefully align the deflection axes and accurately centralize the laser beam. Importantly, not only z focusing but also small extent x and y scanning was performed during 3D drifts with the AO1 and AO2 deflectors, respectively (see for example **Equations S36** and **S62**). The z-focusing unit and the 2D-AO scanning units were linked together by a telecentric relay system formed from two identical achromatic lenses of 150 mm effective focal length (*L3* and *L4*, #47-318, Edmund Optics). The lenses are arranged for best imaging of the back focal plane of the first lens to the image side focal plane of the second lens. These planes are aligned as close as possible to the intermediate planes between deflectors deflecting in the x and y directions, respectively. We used a 2-inch broadband dielectric mirror (BB3-E03, Thorlabs) to make the light way compact (*m17*). The half wave plate (*AHWP10M-980,* Thorlabs) ensures that the second pair of AO deflectors receives the entering light with a linear polarization oriented for optimal diffraction efficiency of the deflectors in the scanning unit. This wave plate is adjusted by monitoring the useful output of the scanner unit, and maximizing the deflected power. The wave plate compensates the rotation of the polarization plane caused by the first deflectors and the subsequent mirrors.

The 2D-AO scanning unit formed by two deflectors (*AO3* and *AO4*, Gooch and Housego) scans laterally and also sets the lateral and longitudinal drift of the focal spot, in cooperation with the z-focusing unit (see for example **Table S1).** The driving signals contain the appropriate radio frequency – time functions that move the focal spot along pre-defined 3D trajectories (**Supplemental Experimental Procedures, Equations S1-S70, Table S1**). Lateral scanning was minimized at the z-focusing and maximized at the 2D-AO scanning units by using the feature that only relative frequences (Δf_{0x} and Δf_{0y}) but not the absolute frequencies ($f_{1x}(0,0)$, $f_{2x}(0,0)$, $f_{1y}(0,0)$, and $f_{2y}(0,0)$) parameters were determined by the starting position, and the speed of the 3D drifts (see for example **Table S1**). The AO deflectors in the scanning unit had the same aperture diameters (15 mm)

and were made in the same crystallographic orientation as the deflectors of the focusing unit to simplify the acoustic driver frequency functions. The whole scanning assembly was based on large-aperture (15 mm) optical components. A first achromatic lens of 200 mm focal length *L5* (#47-319, Edmund Optics) focused the diffracted light beams onto the surface of a specially designed field lens *L6,* AO deflectors are imaged onto the back aperture of the objective by an achromatic lenses L5, L6 and L7(180 mm focal length; QIOPTIQ, G322246525, Linos). The principle and design of the angular dispersion compensation performed by the *L6* lens and entire *L5-L7* assembly is described in detail in Supplementary Note 2 in Katona et al. 2012.

The primary beam splitter (*m18*) was a long-pass dichroic with a cut-on wavelength of 700nm (700dcrxu, Chroma Technology). The beam splitter that separates the red and green channels (*m19*) was a long-pass dichroic with an edge at 600 nm (t600lpxr, Chroma Technology). The green absorption band pass filter (*f1*) was centered at 520 nm with a range of 60 nm (ET520/60m, Chroma Technology), and the red filter (*f4*) was centered at 650 nm with a range of 100 nm (ET650/100m, Chroma Technology). Extra IR filters (*f2* and *f3:* ET700sp-2p8, Chroma Technology) were introduced to both detection pathways to minimize the detection of the IR light backscattered into the photomultiplier units (*PMT1* and *PMT2*). The fluorescent light is collected by planoconvex lens (LA1805-A, Thorlabs) in both the green and the red channels (*L8* and *L9*). Photomultiplier cathode material was GaAsP (H10770PA-40, Hamamatsu). The entire detector unit (*m18, m19, f1-f4, L8, L9, PMT1, PMT2*) was fixed directly to the motorized objective arm to minimize the detection pathway (travelling detector system, Katona et al., 2011, Katona et al., 2012). This therefore maximized detection of backscattered fluorescent light and extended the *in vivo* depth scanning range (with 2D galvo scanning it was over 800 µm, **Movie S13**). We used a water-immersion 20× objective (XLUMPlanFI20×/1.0, Olympus) with a 1.0 numerical aperture or a 25× Nikon objective (CFI75 Apochromat 25xW MP, NA 1.1) for spine imaging.

In summary we made the following modifications to improve the transmission in the 850-950 nm range. i) We used acousto-optic deflectors with a new configuration in which the acoustic wave propagated at a higher angle (10°) relative to the [1,1,0] crystallographic axis. The new transducers also support more RF energy (**Figure S2B**). ii) The new mechanical actuators, with six degrees of mechanical freedom, allowed careful alignment of the deflection axes and accurate centralization. iii) The new Faraday isolator with a center wavelength of 900 nm provided 5-10% higher intensity transmission. iv) The second prism pair was on a two-axis motorized stage and could be shifted using linear slides and stepper motors in two perpendicular directions. Both prism positions and prism distances could be set automatically at each target wavelength (**Figure S1B**). v) We also optimized

the electric circuitry, coupling the radio frequency driver to the piezo transducer, to increase the AO wave generation efficiency and bandwidth. The new circuit transforms the typically low impedance (5-20 Ohms) of the transducer to approach the nominal 50 Ohms at the middle of the band; capacitors and inductivities are added to correct for the mismatch on the sides of the band. The new circuit provides around 10-20% better energy transfer from the driver to the transducer than the former versions. vi) We also used a new method, a UV-curing optical adhesive (NOA61, Thorlabs), to mount mirrors because previous mounting methods which used a rubber-ended screw on the side introduced stress and bent the mirror surface, resulting in a significant wavefront error. Because of the optical improvements and the increase in the efficiency of the radio frequency drive of the AO deflectors, spatial resolution and scanning volume were increased by about 15% and 36-fold, respectively. In this way, the central resolution was 405 nm × 430 nm × 2.3 µm and the maximal z-scanning range in transparent samples was over 1000 µm (**Figures S4**). New software modules were developed for fast 3D dendritic measurements, and to compensate for sample drift.

Optical performance of the 3D DRIFT AO microscope

To characterize the performance of the 3D DRIFT AO microscope we characterized transmission along the z, and lateral (x, y) axes as a function of wavelength. In a separate set of measurement performed on fluorescent beads we also recorded the field of view and resolution as a function of z. The maximal transmission of the 3D DRIFT AO scan head was over 40% at 810 nm, and it decreased as a function of wavelength with a small local maximum at 880 nm (**Figure S2C**). This allowed over 340 mW laser intensities below the objective (XLUMPlanFLN 20×, Olympus) at 880 nm when using a Mai Tai eHP laser with over 2.4 W power. Transmission for the 3D AO scan head at different states of AO z focusing (z profile) showed modest wavelength dependence, becoming slightly wider at longer wavelengths (**Figures S2G and H**). Although the laser intensity dropped relatively rapidly as a function of the AO z focusing parameter, the total z-scanning range with ≥5% relative transmission of the scanner unit was about 1500 µm at 880 nm (**Figure S2H**).

Characterization of the spatial resolution of the 3D DRIFT AO microscope

To characterize the optical properties of the 3D DRIFT AO microscope we performed similar set of measurements as in our earlier work (Katona et al., 2012) but now at 880 nm: i) the maximal lateral field of view was recorded in a 1100 µm AO z-focusing range at 880 nm (**Figure S4**), ii) a 6 µm fluorescent bead was imaged in a 1000 µm AO z-focusing range to demonstrate the preserved good somatic resolution (**Figure S4B**), iii) finally, we characterized the full width at half maximum values

(mean ± SEM) of the point spread function along the z and x axes as a function of distance along the x and z axes (**Figures S4C-F**) using small fluorescent beads (diameter: 170 nm).

Improvement in sensitivity of single AP

A large cranial window was used for the simultaneous electrophysiological (cell-attached) recordings and 3D two-photon imaging experiments to preserve high NA, even at depth. The optical aperture was extended with a U-shaped open hole for the recording pipette. Unfortunately this arrangement did not provide enough stability for the cell-attached recordings during running because the amplitude of the motion artifacts in the voltage signal was about 10-to 100-fold larger than the amplitude of single APs (data not shown). Therefore, to validate the single AP sensitivity of our recordings we performed the combined electrophysiological and imaging measurements on anesthetized mice (**Figure S7A-F**). We were able to detect single AP-induced somatic Ca^{2+} responses (**Figure S7B**). The amplitude of the somatic Ca^{2+} responses was proportional to the number of APs in the spontaneous bursts (**Figure S7E).**

Next, we wanted to show how the use of 3D DRIFT AO scanning can improve single AP detection. As is it was shown, for example, in **Figures 2D, E**, in contrast to the about 10-fold increase in the SNR during running periods, the improvement was only very modest during rest. Therefore, validation had to be done for the running periods where we were not able to perform electrophysiological recording. To do this, we calculated the relative changes in fluorescence intensity of bright fluorescent objects in the brain when they were shifted with the amplitude of brain motion recorded (at 160 Hz) in advance using the fast 3D motion-detection method (**Figure S6**). Although these bright objects did not respond to the Ca^{2+}, motion generated large amplitude fluorescence transients. The amplitude histogram indicated that about 55.35 ± 4.67 % of the motion artifacts were larger than single AP-associated Ca^{2+} responses (**Figure S7F**). With motion correction, this value dropped to 1.66 ± 0.51 % showing the efficiency of the method (**Figure S7F**).

In a different set of measurements we added series of 1, 2, 5, or 10 APs and noise to somatic and dendritic images and then shifted them rapidly with the amplitude of the brain motion detected in advance by using the fast 3D motion-detection method (at 160 Hz) (see section *"Validation of the fast 3D motion-detection method and recording brain movement"* in **Supplemental Experimental Procedures**). Then the SNR of the Ca^{2+} transients with motion correction was compared to those without motion correction (**Figures S8A-H**). Note, the 12.8 ± 3.2 -fold improvement in the SNR for detection of single APs during running when motion correction was used (**Figure S8**).

Processing of 3D data

Most of the analysis, including video rearrangement, motion correction, running average and $\Delta F/F$ calculation was performed with the built-in analysis tools in the acquisition software (MES, Femtonics Ltd., Budapest, Hungary). Raw fluorescence data (F) recorded along ribbons in 3D were spatially normalized, and then projected onto a 2D plot by applying the formula: $\Delta F/F = (F(d_L,d_{tr1},t) - F_0(d_L,d_{tr1}))/F_0(d_L,d_{tr1})$, where t denotes time, and d_L and d_{tr1} indicate the longitudinal and the transversal distance along the ribbon, respectively. A similar form was used for multi-layer, multi-frame imaging and chessboard scanning. To calculate relative fluorescence changes for snake scanning, we added one more spatial dimension (d_{tr2}) as follows: $\Delta F/F = (F(d_L,d_{tr1}, d_{tr2},t) - F_0(d_L, d_{tr1}, d_{tr2}))/F_0(d_L, d_{tr1}, d_{tr2})$. For 3D projection, 3D rendering, and 3D hyperstack analysis we used the ImageJ open-source software with some custom-written macros. A similar formula was used for cube scanning.

Selection of the 3D trajectory and the ribbons for 3D ribbon and snake scanning

To select 3D trajectories, we used the same software modules and a similar method as previously (see Movie S2 in Katona et al. 2011[1]). First a z-stack is taken then the recorded images are used as a reference to select the 3D trajectory. Guiding points are selected for example along the length of dendritic segments using the computer's mouse during scrolling up and down through the depth of the pre-recorded images by using one of the rotating encoders of the microscope control panel (**Movie S3**). After completion of the selection, 3D trajectories are instantaneously fitted to the guiding points, using piecewise cubic Hermite interpolation, and shown in the images of the z-stack in order to visualize misalignment of the 3D trajectory relative to the selected cell segments. Guiding points can be added and modified with an integrated drag-and-drop function to compensate for the misalignment during primary selection of the trajectory, or during long-term measurements. In this way, points can easily be reset to compensate for the movement of the sample. After that, a 3D ribbon is fitted to the 3D curve in such a way that the plane of the 3D ribbon is set to be parallel to the direction of the average brain motion, measured in advance on a bright fluorescent object. Alternatively, we can set the plane of the 3D ribbon to be parallel to the horizontal plane (**Movie S3**). Both methods minimize motion artifacts.

[1] http://www.pnas.org/content/suppl/2011/01/10/1009270108.DCSupplemental/sm02.wmv

Motion correction in 3D

Data resulting from the 3D ribbon scanning, multi-layer, multi-frame scanning, and chessboard scanning methods are stored in a 3D array as time series of 2D frames (**Figure S9A**). The 2D frames are sectioned to bars matching the AO drifts to form the basic unit of our motion correction method (**Figure S9B**). We selected the frame with the highest average intensity in the time series as a reference frame. Then we calculated cross-correlation between each frame and bar and the corresponding bars of the reference frame to yield a set of displacement vectors in the data space. The displacement vector for each frame and for each bar is transformed to the Cartesian coordinate system of the sample, knowing the scanning orientation for each bar (**Figure S9C**). Noise bias is avoided by calculating the displacement vector of a frame as the median of the motion vectors of its bars (**Figure S9D**). This common displacement vector of a single frame is transformed back to the data space. The resulting displacement vector for each bar in every frame is then used to shift the data of the bars using linear interpolation for subpixel precision (**Figure S9E**). Gaps are filled with data from neighboring bars, whenever possible.

The motion correction is executed off-line and, therefore, it was optimized not for the highest speed but for highest resolution, as subpixel resolution is required in compensation (**Figure 2F**). The typical execution time was about a minute. For example, for the four-layer raster scan with 133 frames and 200 x 253 pixels / layer, the motion correction algorithm ran in 66.4 ± 0.25 seconds (**Figure 5**). For the ribbon scanning of 300 frames with 30 x 1633 pixels for the entire measured region, the motion correction took 80.8 ± 0.15 s (**Figure 4**). For the chessboard scanning with 136 ROIs and 25 x 25 pixels per ROI for 154 frames, the run time of the motion correction was 67.21 ± 0.13 seconds (**Figure 3**) using a PC with an i7 processor and 12 GB of memory.

Signal generation and flow in the 3D DRIFT AO scanning two-photon microscope

The electronics of our 3D DRIFT AO microscope were developed from the microscope described in detail in Katona et al. 2012. See the *Supplementary Software 1, 2, 3* and *4* and in the *Supplementary Note 3* and *5* sections of our previous manuscript (Katona et al., 2012). Here, we detail its working principle briefly, focusing on the changes made compared to the microscope described previously. The microscope is developed from the Femto2D microscope (Femtonics Ltd., Budapest, Hungary) as previously. In this study, we improved our 3D AO imaging method (Katona et al., 2012, Chiovini et al., 2014) by using a novel AO signal synthesis card implemented in the electronics system used earlier. The card in its current state allows the generation of 10-142 MHz signals of varying amplitude with frequency chirps implementing linear and quadratic temporal dependence. Synchronizing and

commanding the cards based on **Equations S1-S70** (summarized in **Table S1**) allowed us to arbitrarily place the focal spot and let it drift along any 3D line for every (10-35 µs) AO cycle. We've also added new scanning features to the MES microscope control software (Femtonics Ltd., Budapest, Hungary), and describe here the core of the code responsible for the calculations.

Overall electronics scheme

Four radio frequency D/A converter (DAC) chips were used to generate the sine waves with continuously changing (chirped) frequencies for the four AO deflectors (**Figure S3A**) according to the strategy described in **Supplemental Experimental Procedures** (**Equations 1-70, Table S1**). The chips were built on custom designed multilayer printed circuit boards, incorporating FPGA (field-programmable gate array, Spartan-6, Xilinx) chips to generate their digital driver signal, to synchronize them, and to connect them to the Femto2D microscope's communication system. For each of the four AO deflectors, the synthesized sinusoidal output signal (in the range of 10 – 142 MHz) was coupled to a high gain, broadband amplifier (ZHL-20W-13+, Mini-Circuits) and then to a 3dB attenuator (3dB, 20W, Linearlab) before connecting to the AO deflectors. We measured the back reflection of the radio frequency (RF) driver signal from the broadband amplifier (ZHL-20W-13+, Mini-Circuits) and a successive 3 dB attenuator (3 dB, 20 W, Linearlab) output at each of the AO deflectors directly, and compensated for the RF reflection and loss to distribute RF energy more homogeneously between deflectors. This allowed higher absolute acoustic energy on the crystals, providing higher AO efficiency, and thus higher laser output under the objective and a more homogeneous illumination of the scanning volume (**Figures S2, S4A-F**).

Digital to analog conversion

To generate the frequency sweeps for the four crystals, we used DAC chips (AD9739A, Analog Devices). A detailed description of the functions of the chip can be found on the Analog Devices website[2]. DAC output was generated with an update rate of 1584 MHz according to the data fed by the FPGA. The AD9739A included two LVDS (low-voltage differential signaling) data ports (DB0 and DB1). To reduce the data interface rate to half of the DAC update rate (fDAC), two interleaved 792 MHz streams of 14 bit data were supplied to the DAC. Before operation, the chip is programmed by the FPGA via the AD9739A's SPI (serial programming interface) port. **Supplemental File 1** contains the Verilog code of the FPGA performing signal generation and communication with the DAC chip.

[2] http://www.analog.com/en/products/digital-to-analog-converters/da-converters/ad9739a.html

Signal generation

The frequency and amplitude was generated within the FPGA chips (**Figure S3B**, **Supplemental File 1**). The frequency of the sinusoid output voltage is defined by the 21 bit Frequency Register (FR, **Figure S3B**) allowing 755 Hz frequency generation precision, resulting in ≈30 nm focus pointing precision in the sample. Frequency was incremented at each clock cycle using the Frequency Step Register (FSR**,** 16 bit), while this was advanced with the value of the Frequency Derivate Step Register (FDSR, 16 bit). This mechanism allowed arbitrary sine wave generation with quadratically varied frequency profiles. Amplitude can be varied with 13 bit precision using the Amplitude Offset and Amplitude Multiply Registers (**Figure S3B**). At the end of the chain there is a RAM-table-based amplitude compensation possibility allowing the compensation for frequency-dependent output characteristics of the device. Besides compensating for amplification inhomogeneity, it is possible to compensate for signal loss at the side of the microscope's field of view and also for the amplitude inhomogeneity resulting from the fact that AO deflector driving frequencies continuously change during each focusing event. The FPGA-s internal clock frequency is 99 MHz, thus all register contents (frequency and amplitude) were updated with this pace (internal cycle). To cope with the DAC chip's data rate, 16 parallel calculations are performed at the end when sine waves are finally generated using tables stored in read-only memory blocks, and then finally transferred to the DAC chip (**Supplemental File 1**).

Signal calculation

The driver frequencies of the AO deflector crystals were calculated by the data acquisition computer before 3D scanning by an algorithm described in the calcAOum2Mhz_Drift.m MATLAB program file (**Supplemental File 2**). This routine returns the four center frequencies in MHz (*freq*), the four frequency step speeds in MHz/s (*chirp*), and the four frequency derivate step values in MHz/s^2 (*chirp2*) for any desired point (x, y, and z coordinates) in the field of view with any desired speed vector (v_x, v_y, v_z). During the measurements, coordinates and scanning speeds were defined using graphical software tools by drawing ROIs on the displayed raster-scanned images (similarly to Supplementary Movie 2 in Katona et al. 2011). We calculated the necessary register values for each scanning point using the calculations in the calcAOMhz2Reg.m MATLAB program file (**Supplemental File 3**). Register data were transferred to the FPGA to generate and finally synthetize the RF driving signals with the Femto2D microscope's digital communication system.

Data timing

Measurements were taken according to a fixed IO sequence (AO cycle, see **Figure S3C**) with a predefined IO cycle time matching the aperture of the AO deflectors (down to 18.7 µs corresponding to the 53.3 kHz maximal AO cycle rate; although the electronics allowed AO cycle rates up to 750 kHz, in practice 20-53 kHz rates were used). First the register data (FR, FSR, FDSR; see "*Uploading sweep parameters"* in **Figure S3C**) were uploaded to the FPGA's registers. Then, to start the AO cycle, the AO start synchronization signals (*sync*) were sent to all FPGA chips synchronously (**Figures S3A and S3C**). This actualized the programming registers, set the four starting frequencies and amplitudes and altered them as requested. We sampled all input channels of the Femto2D microscope (PMTs) multiple times before starting the next AO cycle (*"Downloading PMT data" in* **Figure S3C**). The number of samples taken was determined by the maximum available data transfer rate, the preset AO cycle time, and the preset enabling window of the data sampling (eliminating samples taken during the dead time). Streams of stored samples were then formed into images, or volumes, in the MATLAB layer and stored in MES's internal format. The data from the epifluorescent red and green PMTs were treated in parallel as separate channels. Data were analyzed using the MES batch folded-frame-scan, line-scan and curve analysis tools, custom scripts and also by ImageJ macros.

Detailed description of the mathematical background of 3D DRIFT AO scanning

In this section we derive a one-to-one relationship between the focal spot coordinates and speed and the chirp parameters of the AO deflectors to generate fast movement along any straight line segment in the 3D space, starting at any point in the scanning volume.

1. Elaboration of a general expression of the focal depth of the spot for nonlinearly time depending AO frequency chirps

In order to determine the relationship between the driver frequencies of the four AO deflectors and the *x, y* and *z* coordinates of the focal spot, we need the simplified transfer matrix model of the 3D microscope. Our 3D AO system (Katona et al., 2012) is symmetric along the x and y coordinates, because it is based on two x and two y cylindrical lenses, which are symmetrically arranged in the x-z and y-z planes. We therefore need to calculate the transfer matrix for one plane, for example for the x-z plane. The first and second x deflectors of our 3D scanner are in a conjugated focal plane, as they are coupled with an afocal projection lens consisting of two achromatic lenses. For simplicity, therefore, we can use them in juxtaposition during the optical calculations (**Figure S1C**).

In our paraxial model we use two lenses with F_1 and F_2 focal distances at a distance of F_1+F_2 (afocal projection) to image the two AO deflectors (AOD x_1 and AOD x_2) to the objective. $F_{objective}$ is the focal length of the objective, z_x defines the distance of the focal spot from the objective lens along the *z*-axis, and t_1 and t_2 are distances between the AO deflector and the first lens of the afocal projection, and between the second lens and the objective, respectively.

The geometrical optical description of the optical system can be performed by the ABCD matrix technique (Saleh and Malvin, 2001). The angle (α_0) and position (x_0) of the output laser beam of any optical system can be calculated from the angle (α) and position (x) of the incoming laser beam using the ABCD matrix of the system (**Equation S1**):

$$\begin{pmatrix} x_0 \\ \alpha_0 \end{pmatrix} = \mathbf{A} * \begin{pmatrix} x \\ \alpha \end{pmatrix} \quad \textbf{[Equation S1]}$$

The ABCD transfer matrix of the system shown in **Figure S1C** that transfers the rays from the output plane of the second deflector to the focal plane of the objective can be calculated as follows:

$$\boldsymbol{A} = \begin{pmatrix} 1 & z_x \\ 0 & 1 \end{pmatrix} \begin{pmatrix} 1 & 0 \\ \frac{-1}{F_{obj}} & 1 \end{pmatrix} \begin{pmatrix} 1 & t_2 \\ 0 & 1 \end{pmatrix} \begin{pmatrix} 1 & 0 \\ \frac{-1}{F_2} & 1 \end{pmatrix} \begin{pmatrix} 1 & F_1 + F_2 \\ 0 & 1 \end{pmatrix} \begin{pmatrix} 1 & 0 \\ \frac{-1}{F_1} & 1 \end{pmatrix} \begin{pmatrix} 1 & t_1 \\ 0 & 1 \end{pmatrix} \quad \textbf{[Equation S2]}$$

The product of the matrixes is quite complicated in its general form:

$$\boldsymbol{A} = \begin{pmatrix} -\frac{F_2}{F_{obj}F_1}\left(F_{obj} - z_x\right), & F_1 + F_2 - (F_1 + F_2)\frac{z_x}{F_{obj}} - \frac{F_2 t_1}{F_1} - \frac{F_1 t_2}{F_2} - \frac{F_1 z_x}{F_2} + \frac{z_x}{F_{obj}}\left(\frac{F_2 t_1}{F_1} + \frac{F_1 t_2}{F_2}\right) \\ \frac{F_2}{F_1 F_{obj}}, & \frac{F_2 t_1}{F_1 F_{obj}} - \frac{F_1}{F_2 F_{obj}}\left(F_2 + F_{obj} - t_2\right) - \frac{F_2}{F_{obj}} \end{pmatrix}$$

[Equation S3]

However, we can use the simplification below, considering that the afocal optical system produces the image of the deflector output plane on the aperture of the objective lens, with the ideal telescope imaging. In this case, $t_1 = F_1$ and $t_2 = F_2$.

With this simplification we get:

$$A = \begin{pmatrix} -\frac{F_2}{F_{obj}F_1}\left(F_{obj} - z_x\right), & -\frac{F_1 z_x}{F_2} \\ \frac{F_2}{F_1\,F_{obj}}, & -\frac{F_1}{F_2} \end{pmatrix} \quad \text{[Equation S4]}$$

Using this matrix in **Equation S1** we can calculate the angle (α_0) and coordinate (*x_0)* of any output ray in the x-z plane at a given *z* distance (z_x) from the objective from the angle (α) and position (x) taken in the plane of the last AO deflector. The same calculation can be used for the *y-z* plane.

According to the law of AO diffraction the relative angle deflection (α) following the AO deflector is proportional to the local acoustic frequency (*f*):

$$\propto = K * f \quad \text{[Equation S5]}$$

where both α and f are functions of time and the x coordinate. The time-dependence of the frequency of the first AO deflector (*x1*, **Figure S1C**) can be defined as $f_1(t) = f_1(0)+a_{x1}(t)*t$, and the space and time dependency is given as:

$$f_1(x,t) = f_1(0,0) + a_{x1}(t) * \left(t - \frac{D}{2*v_a} - \frac{x}{v_a}\right) \quad \text{[Equation S6]}$$

where *D* is half the diameter of the AO deflector, and v_a is the propagation speed of the acoustic wave within the deflector. Similarly, for the second deflector (AOD x2, **Figure S1C**):

$$f_2(x,t) = f_2(0,0) + a_{x2}(t) * \left(t - \frac{D}{2*v_a} + \frac{x}{v_a}\right) \quad \text{[Equation S7]}$$

where the *x* coordinate has the opposite sign to that in the first deflector because the sound propagates in the opposite direction (**Figure S1C**). Otherwise, they have the same scale, hence the *x* coordinate can be applied to both.

The total deflection angle after the two AO deflectors is proportional to the difference frequencies:

$$\alpha(x,t) = K * (f_1(x,t) - f_2(x,t)) = K * \left(f_1(0,0) - f_2(0,0) + a_{x1}(t) * \left(t - \frac{D}{2*v_a} - \frac{x}{v_a}\right) - a_{x2}(t) * \left(t - \frac{D}{2*v_a} + \frac{x}{v_a}\right)\right) \quad \textbf{[Equation S8]}$$

The x_0 coordinate in the target sample plane can be calculated from the matrix (**Equations S1 and S4**):

$$x_0(t) = -\frac{F_2}{F_{obj}F_1}\left(F_{obj} - z_x\right) * x - \frac{F_1 z_x}{F_2} * \alpha(x,t) \quad \textbf{[Equation S9]}$$

Hence the x-dependent terms in the expression of x_0 are:

$$-\frac{F_2}{F_{obj}F_1}\left(F_{obj} - z_x\right) + K\frac{F_1 z_x}{F_2}\frac{a_{x1}(t)+a_{x2}(t)}{v_a} \quad \textbf{[Equation S10]}$$

In order to focus all the rays on the focus point of the objective, the *x*-dependence of the x_0 coordinate should be zero. If the frequency slopes a_{x1} and a_{x2} are independent of time, we get an expression for a constant focal plane distance from the objective, z_x:

$$z_x = \frac{\frac{F_2}{F_1}}{\frac{F_2}{F_{obj}F_1} + K\frac{F_1}{F_2}\frac{(a_{x1}+a_{x2})}{v_a}} \quad \textbf{[Equation S11]}$$

If we admit the linear temporal dependence of $a_{x1}(t)$ and $a_{x2}(t)$, we will get:

$$a_{x1}(t) = b_{x1} * \left(t - \frac{D}{2v_a} - \frac{x}{v_a}\right) + c_{x1} \; and \; a_{x2}(t) = b_{x2} * \left(t - \frac{D}{2v_a} - \frac{x}{v_a}\right) + c_{x2}$$

[**Equations S12a and S12b**]

To provide ideal focusing, in a first assumption, the time-dependent and -independent terms in the *x*-dependent part of the x_0 coordinate should vanish separately for all *t* values.

$$x_0(t) = -\frac{F_2}{F_{obj}F_1}\left(F_{obj} - z_x\right) * x - \frac{F_1 z_x}{F_2} * K * \left(f_1(0,0) - f_2(0,0) + b_{x1} * \left(t - \frac{D}{2v_a} - \frac{x}{v_a}\right)^2 - b_{x2} * \right.$$

$$\left. * \left(t - \frac{D}{2v_a} + \frac{x}{v_a}\right)^2 + c_{x1} * \left(t - \frac{D}{2v_a} - \frac{x}{v_a}\right) - c_{x2} * \left(t - \frac{D}{2v_a} + \frac{x}{v_a}\right)\right) \quad \text{[Equation S13]}$$

Expanding the terms in brackets, we get separate *x*- and *t*- dependent parts:

$$x_0(t) = -\frac{F_1 z_x}{F_2} * K * (b_{x1} - b_{x2}) * \left(t - \frac{D}{2v_a}\right)^2 - \frac{F_1 z_x}{F_2} * K * (b_{x1} - b_{x2}) * \left(\frac{x}{v_a}\right)^2 + \left(-\frac{F_2}{F_{obj}F_1}\left(F_{obj} - \right.\right.$$

$$\left.\left. -z_x\right) - \frac{F_1 z_x}{F_2} * K * \left[-2 * (b_{x1} + b_{x2}) * \left(t - \frac{D}{2v_a}\right) * \frac{1}{v_a} - (c_{x1} + c_{x2}) * \frac{1}{v_a}\right]\right) * x + \frac{F_1 z_x}{F_2} * K *$$

$$\left(f_1(0,0) - f_2(0,0) + (c_{x1} - c_{x2}) * \left(t - \frac{D}{2v_a}\right)\right) \quad \text{[Equation S14]}$$

To have the beam focused, the terms containing x^2 and *x* must vanish for any x value. This implies two equations instead of only one:

$$-\frac{F_2}{F_{obj}F_1}\left(F_{obj} - z_x\right) + K\frac{F_1 z_x}{F_2}\frac{c_{x1} + c_{x2}}{v_a} + 2 * K\frac{F_1 z_x}{F_2}\frac{b_{x1} + b_{x2}}{v_a} * \left(t - \frac{D}{2v_a}\right) = 0 \quad \text{[Equation S15]}$$

and:

$$K\frac{F_1 z_x}{F_2}\frac{b_{x1} - b_{x2}}{{v_a}^2} = 0 \quad \text{[Equation S16]}$$

The second implies that $b_{x1} = b_{x2} = b_x$. This also implies that the first term on the right side in **Equation S2**, the single one that contains the term depending on t^2, vanishes. Hence we have an x_0 coordinate moving with constant velocity. If this happens at constant *z*, which is not time dependent, and $b_{x1} = b_{x2} = 0$, we get back to the simple linear temporal slope of the acoustic frequencies.

From **Equation S15** we can express the time-dependence of the z coordinate:

$$z_x(t) = \frac{\frac{F_2}{F_1}}{\frac{F_2}{F_{obj}F_1} + K\frac{F_1}{F_2}\frac{c_{x1}+c_{x2}}{v_a} + 4 * K\frac{F_1}{F_2}\frac{z_x b_x}{v_a} * \left(t - \frac{D}{2v_a}\right)} \quad \textbf{[Equation S17]}$$

We will treat separately the cases when the z_x coordinate is constant, hence the focal spot drifts within the horizontal x-y plane (see below **section #2**); and when the spot moves along arbitrary straight lines in the 3D space possibly following the axes of the structures that are measured – e.g. axons, dendrites, etc. (see below **section #3**).

2. The z_x coordinate does not depend on time

In this case, $b_{x1} = b_{x2} = 0$ as we can see from **Equations S16 and S17**. From **Equation S17**, we also see that the focal plane is constant:

$$z_x = \frac{\frac{F_2}{F_1}}{\frac{F_2}{F_{obj}F_1} + K\frac{F_1}{F_2}\frac{c_{x1}+c_{x2}}{v_a}} \quad \textbf{[Equation S18]}$$

If we set a desired z_x plane, we get for the following relationship between the required c_{x1} and c_{x2} parameters:

$$c_{x1} + c_{x2} = v_a \frac{F_2^2}{K * z_x * F_{obj} F_1^2}\left(F_{obj} - z_x\right) \quad \textbf{[Equation S19]}$$

The temporal variation of the x_0 coordinate in this case is given by:

$$x_0(t) = -\frac{F_1 z_x}{F_2} * K * \left(f_1(0,0) - f_2(0,0) + (c_{x1} - c_{x2}) * \left(t - \frac{D}{2v_a}\right)\right) \quad \textbf{[Equation S20]}$$

If we replace z_x with its expression from **Equation S18**, we get for the x_0 coordinate:

$$x_0(t) = -\frac{F_1}{F_2} * \frac{\frac{F_2}{F_1}}{\frac{F_2}{F_{obj}F_1} + K\frac{F_1}{F_2}\frac{c_{x1}+c_{x2}}{v_a}} * K * \left(f_1(0,0) - f_2(0,0) + (c_{x1} - c_{x2}) * \left(t - \frac{D}{2v_a}\right)\right)$$

[**Equation S21**]

after simplification to:

$$x_0(t) = -\frac{K}{\frac{F_2}{F_{obj}F_1} + K\frac{F_1}{F_2}\frac{c_{x1}+c_{x2}}{v_a}} * \left(f_1(0,0) - f_2(0,0) + (c_{x1} - c_{x2}) * \left(t - \frac{D}{2v_a}\right)\right)$$

[**Equation S22**]

We express the initial velocity and acceleration of the focal spot along the x_0 coordinate:

$$v_{x0} = -\frac{K}{\frac{F_2}{F_{obj}F_1}+K\frac{F_1}{F_2}\frac{c_{x1}+c_{x2}}{v_a}} * \left((c_{x1} - c_{x2})\right)$$ [**Equation S23**]

further simplified:

$$v_{x0} = -\frac{K*z_x*F_1}{F_2} * \left((c_{x1} - c_{x2})\right)$$ [**Equation S24**]

and:

$$a_{x0} = -\frac{4K}{\frac{F_2}{F_{obj}F_1}+K\frac{F_1}{F_2}\frac{c_{x1}+c_{x2}}{v_a}} * (b_{x1}) = 0$$ [**Equation S25**]

The last equation shows that in the x-z plane the focal spot cannot be accelerated; it drifts with constant velocity v_{x0}, which is the same for the duration of the frequency chirp's. When we want to calculate the values of the required frequency slopes to get a moving focal point characterized by the following parameters: starting *x* coordinate x_0, distance from the objective z_x, velocity along the x axis v_{x0}, we need to use the expression for $c_{x1} + c_{x2}$ (**Equation S19**) and $c_{x1} - c_{x2}$ **(Equation24).**

For c_{x1} and c_{x2} we get:

$$c_{x1} - c_{x2} = \frac{-F_2}{K*F_1*z_x}(v_{x0})$$ [**Equation S26**]

$$c_{x1} + c_{x2} = v_a\frac{F_2^2}{K*z_x*F_{obj}*F_1^2}\left(F_{obj} - z_x\right)$$ [**Equation S27**]

Adding and subtracting the above two equations, we get the results:

$$c_{x1} = \frac{-F_2}{2*K*F_1*z_x} * \left(v_{x0} - \frac{v_a*F_2}{F_1*F_{obj}} \left(F_{obj} - z_x \right) \right) \quad \textbf{[Equation S28]}$$

$$c_{x2} = \frac{f_2}{2*K*F_1*z_x} * \left(v_{x0} + \frac{v_a*F_2}{F_1*F_{obj}} \left(F_{obj} - z_x \right) \right) \quad \textbf{[Equation S29]}$$

In summary, we can say that it is possible to drift the focal spot at a constant velocity along lines lying in horizontal planes (perpendicular to the objective axis); the focal distance z_x can be set by the acoustic frequency chirps in the AO deflectors. The ranges of z_x and v_{x0} available cannot be deduced from this analysis, they are limited by the frequency bandwidths of the AO devices that limit the temporal length of the chirp sequences of a given slope.

3. The z_x coordinate depends on time

If we want to drift the spot in the sample space along the z axis within one AO switching time period, we have to allow for temporal change of the z_x coordinate. The formula:

$$z_x(t) = \frac{\frac{F_2}{F_1}}{\frac{F_2}{F_{obj}F_1} + K \frac{F_1}{F_2} \frac{a_{x1}(t) + a_{x2}(t)}{v_a}} \quad \textbf{[Equation S30]}$$

comes from the constraint to focus all rays emerging from the AO cells onto a single focal spot after the objective (see **Equation S18** for the time-independent z_x).

From **Equations S12, S13, and S30** we get:

$$-\frac{F_2}{F_{obj}F_1} \left(F_{obj} - z_x \right) + K \frac{F_1 z_x}{F_2} \frac{c_{x1} + c_{x2}}{v_a} + 2 * K \frac{F_1 z_x}{F_2} \frac{b_{x1} + b_{x2}}{v_a} * \left(t - \frac{D}{2v_a} \right) = 0 \quad \textbf{[Equation S31]}$$

hence:

$$z_x(t) = \frac{\frac{F_2}{F_1}}{\frac{F_2}{F_{obj}F_1} + K \frac{F_1}{F_2} \frac{c_{x1} + c_{x2}}{v_a} + 2*K \frac{F_1}{F_2} \frac{b_{x1} + b_{x2}}{v_a} * \left(t - \frac{D}{2v_a} \right)} \quad \textbf{[Equation S32]}$$

This equation has, however, a non-linear temporal dependence. Therefore, we need its Taylor series to simplify further calculations:

$$z_x(t) = \frac{\frac{F_2}{F_1}}{\frac{F_2}{F_{obj}F_1} + K\frac{F_1}{F_2} * \frac{c_{x1}+c_{x2}}{v_a} - 2 * K\frac{F_1}{F_2}\frac{b_{x1}+b_{x2}}{v_a} * \left(\frac{D}{2v_a}\right)} + \frac{-2K\frac{b_{x1}+b_{x2}}{v_a}}{\left(\frac{F_2}{F_{obj}F_1} + K\frac{F_1}{F_2} * \frac{c_{x1}+c_{x2}}{v_a} - 2 * K\frac{F_1}{F_2}\frac{b_{x1}+b_{x2}}{v_a} * \left(\frac{D}{2v_a}\right)\right)^2} * t + \frac{\left(2K\frac{b_{x1}+b_{x2}}{v_a}\right)^2 * \frac{F_1}{F_2}}{\left(\frac{F_2}{F_{obj}F_1} + K\frac{F_1}{F_2} * \frac{c_{x1}+c_{x2}}{v_a} - 2 * K\frac{F_1}{F_2}\frac{b_{x1}+b_{x2}}{v_a} * \left(\frac{D}{2v_a}\right)\right)^3} * t^2 + \cdots$$

[**Equation S33**]

To have a nearly constant velocity, the second and higher order terms in the Taylor series should be small, or nearly vanish: this imposes constraints on the b_{x1}, b_{x2}, c_{x1}, and c_{x2} values. Our simplest presumption is that the linear part will dominate time dependence over the quadratic part, which means that the ratio of their coefficients should be small:

$$\frac{\left(K\frac{b_{x1}+b_{x2}}{v_a}\right) * \frac{F_1}{F_2}}{\left(\frac{F_2}{F_{obj}F_1} + K\frac{F_1}{F_2} * \frac{c_{x1}+c_{x2}}{v_a} - 2 * K\frac{F_1}{F_2}\frac{b_{x1}+b_{x2}}{v_a} * \left(\frac{D}{2v}\right)\right)} \ll 1$$ [**Equation S34**]

However, the second member in the sum, the velocity along the z axis in the z-x plane (v_{zx}), is also similarly expressed:

$$v_{zx} = \frac{\left(-2K\frac{b_{x1}+b_{x2}}{v_a}\right)}{\left(\frac{F_2}{F_{obj}F_1} + K\frac{F_1}{F_2} * \frac{c_{x1}+c_{x2}}{v_a} - 2 * K\frac{F_1}{F_2}\frac{b_{x1}+b_{x2}}{v_a} * \left(\frac{D}{2v_a}\right)\right)^2}$$ [**Equation S35**]

From **Equation S16** we have $b_{x1} = b_{x2} = b_x$, and this is not zero in this case. We need other constraints to express b_x, and further constants.

The formula for the x_0 coordinate (from **Equation S9**) is:

$$x_0(t) = -\frac{F_2}{F_{obj}F_1}\left(F_{obj} - z_x(t)\right) * x - \frac{F_1 z_x(t)}{F_2} * \alpha(x,t)$$

$$= -\frac{F_2}{F_{obj}F_1}\left(F_{obj} - \frac{\frac{F_2}{F_1}}{\frac{F_2}{F_{obj}F_1} + K\frac{F_1}{F_2}\frac{c_{x1}+c_{x2}}{v_a} + 2 * K\frac{F_1}{F_2}\frac{b_{x1}+b_{x2}}{v_a} * \left(t - \frac{D}{2v_a}\right)}\right) * x$$

$$- \frac{\frac{F_2}{F_1}}{\frac{F_2}{F_{obj}F_1} + K\frac{F_1}{F_2}\frac{c_{x1}+c_{x2}}{v_a} + 2 * K\frac{F_1}{F_2}\frac{b_{x1}+b_{x2}}{v_a} * \left(t - \frac{D}{2v_a}\right)} * \frac{F_1}{F_2} * K$$

$$* \left(f_{x1}(0,0) - f_{x2}(0,0) + (b_{x1}) * \left(t - \frac{D}{2v_a} - \frac{x}{v_a}\right)^2 - (b_{x2}) * \left(t - \frac{D}{2v_a} + \frac{x}{v_a}\right)^2\right.$$

$$\left. + (c_{x1}) * \left(t - \frac{D}{2v_a} - \frac{x}{v_a}\right) - c_{x2} * \left(t - \frac{D}{2v_a} + \frac{x}{v_a}\right)\right)$$

$$= -\frac{1}{\frac{F_2}{F_{obj}F_1} + K\frac{F_1}{F_2}\frac{c_{x1}+c_{x2}}{v_a} + 2 * K\frac{F_1}{F_2}\frac{b_{x1}+b_{x2}}{v_a} * \left(t - \frac{D}{2v_a}\right)} * K$$

$$* \left[f_{x1}(0,0) - f_{x2}(0,0) + \left(t - \frac{D}{2v_a}\right) * (c_{x1} - c_{x2})\right]$$

[**Equation S36**]

To find the drift velocity along the *x* axis we should differentiate the above function with respect to *t*:

$$v_x(t) = \frac{dx_0(t)}{dt} = +\frac{2 * K\frac{F_1}{F_2}\frac{b_{x1}+b_{x2}}{v_a}}{\left(\frac{F_2}{F_{obj}F_1} + K\frac{F_1}{F_2}\frac{c_{x1}+c_{x2}}{v_a} + 2 * K\frac{F_1}{F_2}\frac{b_{x1}+b_{x2}}{v_a} * \left(t - \frac{D}{2v_a}\right)\right)^2} * K$$

$$* \left[f_{x1}(0,0) - f_{x2}(0,0) + \left(t - \frac{D}{2v_a}\right) * (c_{x1} - c_{x2})\right]$$

$$- \frac{1}{\frac{F_2}{F_{obj}F_1} + K\frac{F_1}{F_2}\frac{c_{x1}+c_{x2}}{v_a} + 2 * K\frac{F_1}{F_2}\frac{b_{x1}+b_{x2}}{v_a} * \left(t - \frac{D}{2v_a}\right)} * K * [(c_{x1} - c_{x2})]$$

[**Equation S37**]

Taken at t = 0, we can determine the initial value v_{x0} of the drift velocity component along the x axis:

$$v_{x0} = +\frac{2*K\frac{F_1}{F_2}\frac{b_{x1}+b_{x2}}{v_a}}{\left(\frac{F_2}{F_{obj}F_1}+K\frac{F_1}{F_2}\frac{c_{x1}+c_{x2}}{v_a}+2*K\frac{F_1}{F_2}\frac{b_{x1}+b_{x2}}{v_a}*\left(\frac{D}{2v_a}\right)\right)^2}*K$$
$$*\left[f_{1x}(0,0)-f_{2x}(0,0)+\left(-\frac{D}{2v_a}\right)*(c_{x1}-c_{x2})\right]$$
$$-\frac{1}{\frac{F_2}{F_{obj}F_1}+K\frac{F_1}{F_2}\frac{c_{x1}+c_{x2}}{v_a}+2*K\frac{F_1}{F_2}\frac{b_{x1}+b_{x2}}{v_a}*\left(-\frac{D}{2v_a}\right)}*K*[(c_{x1}-c_{x2})]$$

[**Equation S38**]

If we take b_x from the expression of v_{zx} (**Equation S35**), and introduce it into **Equation S38** we will have an equation (**Equation S39**) that gives a constraint for the choice of c_{x1} and c_{x2}. This constraint relates c_{x1} and c_{x2} to v_{x0} and v_{zx}:

$$K*(v_x-r*\Delta f_{0x}*v_{zx})*\left(1-\frac{v_{zx}*D}{v_a*F_{obj}}\right)*q$$
$$=\frac{F_2}{F_{obj}F_1}*(v_x-r*\Delta f_{0x}*v_{zx})^2+\frac{v_{zx}*D*r^2}{{v_a}^2}*(v_x-r*\Delta f_{0x}*v_{zx})*p*q+\frac{r}{v_a}$$
$$*(v_x-r*\Delta f_{0x}*v_{zx})^2*p+\frac{{v_{zx}}^2*D^2*r^3}{4*{v_a}^3}*p*q^2+\frac{{v_{zx}}^2*D^2*r*K}{4*{v_a}^2*F_{obj}},*q^2$$

[**Equation S39**]

Here we introduced the following notations:

$$r := K\frac{F_1}{F_2}$$ [**Equation S40**]

$$\Delta f_0 := f_{1x}(0,0)-f_{2x}(0,0)$$ [**Equation S41**]

$$q := c_{x1}-c_{x2}$$ [**Equation S42**]

$$p := c_{x1}+c_{x2}$$ [**Equation S43**]

We can express p from **Equation S39,** resulting in a relationship between p and q:

$$p = \frac{\left(1-\frac{D*v_{zx}}{v_a*F_{obj}}\right)*K*H*q-\frac{F_2}{F_1*F_{obj}}*H^2-\frac{v_{zx}^2*D^2*r*K}{4*v_a^2*F_{obj}}*q^2}{\frac{r}{v_a}*\left(\frac{v_{zx}*D*r*q}{2*v_a}+H\right)^2} \quad \textbf{[Equation S44]}$$

where we introduced the notation:

$$H \coloneqq v_x - r*\Delta f_{0x}*v_{zx} \quad \textbf{[Equation S45]}$$

These are general equations that apply to all possible trajectories. Practically, we can analyze the motion of the spot along different trajectories separately.

4. Motion in 3D space along straight line segments

A practically important possibility would be to set a linear trajectory for the drifting spot, following e.g. the axis of a measured dendrite or axon. This is a general 3D line, with arbitrary angles relative to the axes. The projections of this 3D line onto the x-z and y-z planes are also lines that can be treated separately. We are dealing now with the projection on the x-z plane. The projection on the y-z plane can be handled similarly; they are however not completely independent, as will be shown later. If the spot is accelerated on the trajectory, the acceleration and initial velocity are also projected on the x-z and y-z planes. We name the two orthogonal components of the initial velocity in the x-z plane as v_{x0} and v_{zx0} which are parallel to the x and z axis, respectively. Therefore, in the x-z plane we have for the projection of the line trajectory:

$$z(t) = \frac{v_{zx0}}{v_{x0}} * x_0(t) + n \quad \textbf{[Equation S46]}$$

To calculate the chirp parameters we must insert the temporal dependence of the *z(t)* and $x_0(t)$ functions, expressed in the **Equations S33** and **S36**, respectively.

We introduce the following notations:

$$\tilde{u} := \frac{F_2}{F_{obj}F_1} + K\frac{F_1}{F_2}\frac{c_{x1}+c_{x2}}{v_a} \text{ [Equation S47]}$$

$$B = -2 * K\frac{F_1}{F_2}\frac{b_{x1}+b_{x2}}{v_a} \text{ [Equation S48]}$$

$$t' = t - \frac{D}{2v_a} \text{ [Equation S49]}$$

$$M = \frac{F_2}{F_1} \text{ [Equation S50]}$$

Introducing these notations and the temporal dependences from **Equations S33** and **S36** into **Equation S46**, we get the projection of the 3D line:

$$\frac{\mathrm{M}}{\tilde{u}-\mathrm{B}*\mathrm{t}'} = -\frac{v_{zx0}}{v_{x0}} * \frac{\mathrm{K}}{\tilde{u}-\mathrm{B}*\mathrm{t}'}(\mathrm{q} * \mathrm{t}' + \Delta \mathrm{f}_{0x}) + \mathrm{n} \quad \text{[Equation S51]}$$

After some simplification we get:

$$M = -\frac{v_{zx0}}{v_{x0}} * K * \Delta f_{0x} + n * \tilde{u} - \left(\frac{v_{zx0}}{v_{x0}} * K * q + n * B\right) * t' \quad \text{[Equation S52]}$$

This equation must be fulfilled for each time point t’. To be valid for each t’, we must impose the following:

$$M + \frac{v_{zx0}}{v_{x0}} * K * \Delta f_{0x} - n * \tilde{u} = 0 \quad \text{[Equation S53]}$$

and:

$$\frac{v_{zx0}}{v_{x0}} * K * q + n * B = 0 \quad \text{[Equation S54]}$$

The first equation (**Equation S53**) gives:

$$\tilde{u} = \frac{M + \frac{v_{zx0}}{v_{x0}} * K * \Delta f_{0x}}{n} \text{ [Equation S55]}$$

Introducing $\tilde{u}$ from **Equation S47**:

$$\frac{F_2}{F_{obj}F_1} + K\frac{F_1}{F_2}\frac{c_{x1}+c_{x2}}{v_a} = \frac{M+\frac{v_{zx0}}{v_{x0}}*K*\Delta f_{0x}}{n} \quad \textbf{[Equation S56]}$$

From this equation we can express *p* (defined by **Equation S43**) as follows:

$$p = c_{x1} + c_{x2} = \frac{v_a*M}{K} * \left(\frac{M+\frac{v_{zx0}}{v_{x0}}*K*\Delta f_{0x}}{n} - \frac{M}{F_{obj}}\right) \textbf{[Equation S57]}$$

To express $b_{x1} = b_{x2} = b$ and $q = c_{x1} - c_{x2}$, we need another constraint, that can be set from the desired value of the initial velocity v_{zx0}.

We take the derivative of *z(t)* (**Equation S33**) at *t = 0*, to find the initial velocity value, using the notations in **Equations S47** and **S48**:

$$v_{zx0} := v_{zx}(0) = -\frac{B*M}{\tilde{u}^2} \textbf{[Equation S58]}$$

Expressing *B* from **Equation S58**:

$$B = -\frac{v_{zx0}}{M} * \left(\frac{M+\frac{v_{zx0}}{v_{x0}}*K*\Delta f_{0x}}{n}\right)^2 \quad \textbf{[Equation S59]}$$

Introducing the expression of *B* from **Equation S48**, we can yield the parameter *b*:

$$b = \frac{v_{zx0}*v_a}{4*K} * \left(\frac{M+\frac{v_{zx0}}{v_{x0}}*K*\Delta f_{0x}}{n}\right)^2 \textbf{[Equation S60]}$$

To express *q* (defined by **Equation S42**) **we use Equations S54 and S59**:

$$q = c_{x1} - c_{x2} = n * \frac{v_{x0}}{M*K} * \left(\frac{M+\frac{v_{zx0}}{v_{x0}}*K*\Delta f_{0x}}{n}\right)^2 \quad \textbf{[Equation S61]}$$

Finally, we can express c_{x1} and c_{x2} by adding and subtracting *q and p* (**Equations S57 and S61)**:

$$c_{x1} = \frac{M*v_a}{2*K} * \left(\frac{M+\frac{v_{zx0}}{v_{x0}}*K*\Delta f_{0x}}{n} - \frac{M}{F_{obj}}\right) + \frac{v_{x0}*n}{2*K*M} * \left(\frac{M+\frac{v_{zx0}}{v_{x0}}*K*\Delta f_{0x}}{n}\right)^2 \quad \textbf{[Equation S62]}$$

and:

$$c_{x2} = \frac{M*v_a}{2*K} * \left(\frac{M+\frac{v_{zx0}}{v_{x0}}*K*\Delta f_{0x}}{n} - \frac{M}{F_{obj}}\right) - \frac{v_{x0}*n}{2*K*M} * \left(\frac{M+\frac{v_{zx0}}{v_{x0}}*K*\Delta f_{0x}}{n}\right)^2 \quad \text{[Equation S63]}$$

The crucial parameter Δf_{0x} can be calculated from the initially set *$x_0(0)$ at t′ = 0. We then have:*

$$\Delta f_{0x} = \frac{x_0(0)*F_2}{K*F_{obj}*F_1} \quad \text{[Equation S64]}$$

Some characteristic parameters of our AO devices are: *K* = 0.002 rad/MHz, v = 650*10^6 µm/s, the magnification M = 1, the initial frequency difference Δf = 10 MHz, and the movement parameters: m = 2, v_{z0} = 1 µm/µs, n = $f_{objective}$–4 µm. For these values, the c_{x1} value results in 3 kHz/µs, whereas c_{x2} = 17 kHz/s. The acceleration a_{zx} in the z direction is approximately 0.1 m/s^2 with these parameters.

Finally, we summarize our results. Here we demonstrate how it is possible to calculate the parameters for the non-linear chirped driver function, in order to move the focal spot from a given point with a given initial speed along a line path in the x-z plane. The parameters of the line path are selected according to the general formula, in 3D:

$$x_0(t) = x_0(0) + t * v_{x0}$$

$$y_0(t) = y_0(0) + t * v_{y0} \quad \textbf{[Equations S65]}$$

$$z_0(t) = z_0(0) + t * v_{z0}$$

Since the deflectors are deflecting in the x-z and y-z planes, transforming **Equation S65** into the equations that describe the line projections on these planes:

$$z_x(t) = m * x_0(t) + n = z_0(0) + \frac{v_{zx0}}{v_{x0}} * x_0(t) - \frac{v_{zx0}}{v_{x0}} * x_0(0) \quad \text{[Equation S66a]}$$

$$z_y(t) = k * y_0(t) + l = z_0(0) + \frac{v_{zy0}}{v_{y0}} * y_0(t) - \frac{v_{zy0}}{v_{y0}} * y_0(0) \quad \text{[Equation S66b]}$$

With these, we imply that $v_{zx0} = v_{zy0} = v_{z0}$, and:

$$m = \frac{v_{zx0}}{v_{x0}} \quad \text{[Equation S67]}$$

$$k = \frac{v_{zx0}}{v_{y0}} \quad \textbf{[Equation S68]}$$

$$n = z_0(0) - \frac{v_{zx0}}{v_{x0}} * x_0(0) \quad \textbf{[Equation S69]}$$

$$l = z_0(0) - \frac{v_{zx0}}{v_{y0}} * y_0(0) \quad \textbf{[Equation S70]}$$

To steer the deflectors, we need to determine the Δf_{0x}, b_{x1}, b_{x2}, c_{x1}, and c_{x2} parameters in the x-z plane as a function of the selected $x_0(0)$, $z_0(0)$, v_{x0}, and v_{zx0} parameters of the trajectory and drift. The same is valid for the y-z plane: here we determine Δf_{0y}, b_{y1}, b_{y2}, c_{y1}, and c_{y2} for the desired $y_0(0)$, $z_0(0)$, v_{y0}, and v_{zy0} of the trajectory. These transformations are summarized in **Supplemental Table 1**. Note that Δf_{0x} and Δf_{0y} are not fully determined; here we have an extra freedom to select from frequency ranges of the first (f_1) and second (f_2) group of AO deflectors to keep them in the middle of the bandwidth during 3D scanning.

The spot will then keep its shape during the drift, since the corresponding constraint is fulfilled in both planes. The initial velocities v_{x0} and v_{y0} along the x and y coordinates determine the *m* and *k* parameters, together with the initial velocity $v_{zx} = v_{zy}$ set for z (**Equations S67 and S68**) and the acceleration values are also determined by these parameters. The resulting acceleration values are usually low within the practical parameter sets, therefore the velocity of the spot will not change drastically for trajectories which are not too long.

Limitations of 3D DRIFT AO microscopy

All scanning patterns demonstrated in the manuscript are composed of a series of straight 3D line sections generated by 3D DRIFT AO scanning (from now on defined as “drifts”). The 3D position and, size, and the 3D angle of each drift are unrelated to the next drift (random access positioning). Therefore, in the first approach, we determined the limitations of arbitrary scanning patterns by providing the limiting factors of drifts. These are the followings: i) optics; ii) AO deflector signal generation electronics and bandwidth of the acousto-optic deflectors; iii) AO cycle time; and iv) elongation of the PSF during the fast 3D drifts.

i) Optics. 3D drifts are optically limited by the projector optics and the objective to the maximal 3D scanning volume which has already been demonstrated above (see **Figures S4**).

ii) Signal generation electronics and bandwidth of the acousto-optic deflectors***.*** The AO signal generator electronics did not limit the speed of drifts, as the FPGA can generate sweeps traversing the entire bandwidth (from 10 MHz to 142 MHz) within 0.66 µs, which is a much shorter time than the 18.8 µs switching time calculated from the maximal AO cycle frequency (53 kHz). We demonstrated high drift speeds of up to 10 mm/ms (**Figure S5B**). The values of possible v_{x0} velocities depend on the starting x_0 and $z_x = z_y$ parameters. When the acoustic frequency bandwidth is identical for both deflectors, the limitation in the velocity range is that the time needed for the frequency to change from the starting frequency to the end frequency of a sweep sequence (at limit this is the edge of the bandwidth) must be bigger than the aperture time D/v_a. For $x_0(0)=0$ values we get the highest range for the velocity v_x, in this case the frequency goes through a half bandwidth during one sweep sequence, hence:

$$\frac{-K*v_a*BW/2}{M*D} + M * v_a * \left(1 - \frac{z_x}{F_{obj}}\right) < v_x < \frac{K*v_a*BW/2}{M*D} - M * v_a * \left(1 - \frac{z_x}{F_{obj}}\right) \quad \text{[Equation S71]}$$

The same holds also for the y direction. The limits of v_x and v_y are also depending on the $z–F_{obj}$ coordinate difference, since part of the available bandwidth is used to change the z distance during sweeping.

iii) AO cycle time. The drift time was limited by the AO cycle time (30-53 kHz). An AO cycle time below 18.8 µs caused a marked reduction in the signal strength.

iv) Elongation of the PSF. Fast 3D drifts can elongate the PSF due to two factors. First, movement of the PSF within a single PMT integration time window will be relevant at higher 3D drift speeds and, second, AO deflector pointing and focusing could be worse at higher speeds. To measure the net effect of these two factors, we imaged fluorescent beads with fast 3D DRIFT AO scanning (**Figure S5**). We found that at very high scanning speeds PSF is indeed elongated; however, this elongation is mainly governed by the smoothing effect of the movement of the PSF within a single PMT integration period (**Figures S5B and S5C**). This means that PSF elongation can be canceled by simply increasing PMT sampling speed in a future version of the electronics. Also, PSF elongation is less than 20% at drift speed matching scanning speed used elsewhere for LOTOS scanning (Chen et al., 2011).

Limitation for volume and surface elements

In the previous paragraph we summarized the limitation of individual drifts. If we generate surface or volume elements, we need to use multiple drifts. In this case we need to consider the following limitations for the repetition rate of the measurement:

$$v_{measurement} = \frac{AO_v}{\left\lceil \frac{pixel_x}{K} \right\rceil * pixel_y * pixel_z * N} \qquad \textbf{[Equation S72]}$$

where $v_{measurement}$ is in frames per second or volumes per second, AO_v is the frequency of the AO scanning cycle (30-53 kHz) and $\lceil \propto \rceil$ indicates the ceiling (the smallest integer greater than or equal to $\propto$). *N* is the number of (same-sized) ROIs, and $pixel_x$ and $pixel_y$ are the number of pixels required to resolve each ROI along the fast (corresponding to $pixel_x$) and slow axes (corresponding to $pixel_y$ and $pixel_z$) of 3D scanning. $Pixel_z$ =1 used for methods with surface scanning. The fast axis is the direction of the fast 3D AO drifts, and the slow axes are perpendicular to that. *K* is the number of pixels sampled in one AO cycle. The maximal value of *K* in the current system is (*PMT sampling rate*)/*AOv* = 10.5 MHz / 53 kHz ≈ 200. For scanning of *N* different regions we need to use a modified formula:

$$v_{measurement} = \frac{AO_v}{\sum_{i=1}^{N} \left(\left\lceil \frac{pixel_{x_i}}{K} \right\rceil * pixel_{y_i} * pixel_{z_i} \right)} \qquad \textbf{[Equation S73]}$$

The maximal value of N can be high as it is only limited by MATLAB and the memory of the PC. Simultaneous measurement of 530,000 ROIs was possible (data not shown).

The maximal bandwidth of the AO deflectors of ≈60 MHz (**Figure S2B**) can be addressed with 4 kHz accuracy, providing a maximal resolution of 15,000 pixels, equivalent to 33 nm spatial accuracy (discretization) for the ≈500 µm lateral field of view. However, the maximal useful pixel number is limited by the optical resolution of the system (**Figures S4**). Calculating this by using the average lateral resolution (0.8 µm) for the maximal field of view (500 µm), and considering the Nyquist law, the maximal number of useful pixels is about (500/0.8) *2 = 1250 pixels. For the z direction: (700/4.5) *2 = 311, since the average optical resolution along the z axis is 4.5 µm.

Example

According to **Equations S72 and S73** the measurement speed is inversely proportional to the number of (same sized) ROIs. For example, 100 ROIs with 10 x 50 pixel frames can be measured with 53,000

Hz / (1 × 10 × 1 × 100) = 53 Hz according to **Equation S72,** and the speed is 10-fold lower (5.3 Hz) when considering 1000 neurons.

Validation of the fast 3D motion-detection method and recording brain movement

To validate the fast 3D motion-detection method (shown in **Figure 2A**) with an independent technique, we moved fluorescent beads along the x, y, or z axes by using fast piezo actuators (ASM003 Thorlabs) driven by sine wave in the 0-160 Hz frequency range (**Figure S6A**). The high resonant frequency (2 kHz) of the piezo actuator provided a flat mechanical transmission in the 0-160 Hz frequency range. We recorded the position of the fluorescent beads by imaging them with the fast 3D motion-detection method by using small xy and xz frames (xy: 15 µm × 15 µm; xz: 15 µm × 30 µm) which were centered to the beads. To determine the position of the moving beads along a given axis, the recorded fluorescence was projected onto that particular axis and the maximum was determined by Gaussian fitting at each time point (**Figure S6B**). Then we plotted the position of the maximums as a function of time and fitted it with a sine curve to determine amplitude and phase shifts as a function of the driver frequency (**Figure S6C**). The standard deviation of the difference between the recorded bead position and the fitted sine wave was small ($SD_{moving\ pos.\ err.}$= 0.109 ± 0.004 µm), being similar to the standard deviation of the position of the beads which were fixed to the animal holders ($SD_{fixed\ bead\ pos.}$= 0.075 ± 0.031 µm). These low standard deviations, and the flat amplitude and phase transfer functions of the position measurements (**Figure S6D**) demonstrated that the fast 3D motion-detection method can be used to record brain motion in the 0-160 Hz range during *in vivo* recordings.

Next we recorded the position of small fluorescent objects during *in vivo* recordings by imaging them using the fast 3D motion-detection method by using small xy and yz frames (xy: 15 µm × 15 µm; xz: 15 µm × 20 µm) which were centered to the small fluorescent objects. To calculate movement along a given axis, fluorescence data were projected to that particular axis and the position of the object was determined by Gaussian fitting at each time point as before (**Figure S6E**). In parallel, we recorded movement of mice on the virtual reality wheel (**Supplemental Experimental Procedures**) to separate periods of movement and rest (**Figure S6F**). Then we calculated the average Fourier spectra of the movement of bright objects during the running and rest periods (30 s long transients, n=15-17 per case) and compared it to the spectrum of fixed fluorescent beads (**Figure S6G**). Motion artifacts in awake animals contributed only to the 0-50 Hz range of the Fourier spectra (**Figure S6G**). In summary, these data showed that the fast 3D motion-detection method has the required bandwidth to detect *in vivo* motion with about 0.1 µm accuracy; therefore, it was used to

detect motion trajectories from behaving animals. The data recorded by the fast 3D motion-detection method were then used to quantify the motion elimination capability of the new scanning methods of the 3D DRIFT AO microscopy (**Figures 2 and S8A-S8H**).

SUPPLEMENTAL NOTE

SNR and speed improvement for 2D scanning

To record neuronal signaling and action potentials (APs) high repetition rates are necessary. When measuring the light signal from an ROI, noise is dominated by the photon shot noise. As we measure a low number of photons (λ), the mean signal (μ) captured from an ROI and its variance (σ) follow a Poisson distribution:

$$SNR := \frac{\mu}{\sqrt{\sigma}} = \frac{e\lambda}{e\sqrt{\lambda}} = \sqrt{\lambda} \quad \textbf{[Equation S74]}$$

where e means the instrument response amplitude for a single photon. Thus the SNR is determined by the total number of photons collected from a structure within a unit of time. On the other hand, the total number of photons can be calculated as:

$$\lambda = \eta \cdot \varphi \cdot \tau \quad \textbf{[Equation S75]}$$

where η is the system's total efficiency of detecting an emitted photon (constant), ϕ is the emitted photon flux, and τ is the time spent on the ROI. Because increasing excitation in living samples quickly results in photobleaching and photodamage, we cannot increase ϕ above a certain level (determined experimentally for each sample preparation), so the SNR can only be increased by the time fraction spent on the ROIs. To increase SNR we should stop recording entire images, and instead of sampling all the pixels in images, we need to scan repetitively only the regions containing the information required.

Scanning only the ROIs allows the time fraction that the scanner spends on the ROIs to be increased and, therefore, ultimately increases the SNR of the information collected from the ROIs. Here's one example: we want to record activity in *n* ROIs, each of area *a*, situated in one focal plane by using raster scanning. If we use a classical raster scanning method, we need to record the overall frame of area *A* which includes all ROIs. For one ROI we can calculate the SNR as follows (see **Equations S74** and S**75**):

$$(SNR_{raster})^2 = \lambda_{raster} = \eta \cdot \varphi \cdot \tau_{raster} = \eta \cdot \varphi \cdot \frac{a}{A} \cdot \mathrm{T}_{raster} \quad \textbf{[Equation S76]}$$

where τ_{raster} is the time spent in a single raster during the total measurement time, T_{raster} is the total measurement time and λ_{raster} is the total number of photons collected from the ROI in T_{raster} time. If we take advantage of random-access scanning methods by scanning only these *n* ROIs, we can calculate the SNR of one ROI as follows:

$$(SNR_{ROI})^2 = \lambda_{ROI} = \eta \cdot \varphi \cdot \tau_{ROI} = \eta \cdot \varphi \cdot \frac{a}{n \cdot a} \cdot \mathrm{T}_{ROIs} \qquad \textbf{[Equation S77]}$$

where T_{ROIs} is the total measurement time, λ_{ROI} is the total number of photons collected from one ROI using ROI scanning, τ_{ROI} is the total time spent on one ROI during the T_{ROIs} measurement time.

If we now divide **Equation S76** by **Equation S77** we get the increase in the SNR of ROI scanning over that of raster scanning:

$$\left(SNR_{gain}\right)^2 := \frac{(SNR_{ROI})^2}{(SNR_{raster})^2} = \frac{A}{n \cdot a} * \frac{\mathrm{T}_{ROIs}}{\mathrm{T}_{raster}} \qquad \textbf{[Equation S78]}$$

This means that if the ROIs are small and dispersed in the (2D) field of view (FOV), which is the usual case, *A* will be larger compared than $n \cdot a$, and thus the SNR can be increased significantly by repetitively scanning only the information-containing areas, instead of raster scanning the full (mostly not labeled) area. Quantitatively, the SNR_{gain} is equal to the ratio of the total raster scanned area (*A*) versus the area of the ROIs $(n \cdot a)$ when the total scanning time is kept constant ($T_{ROIs} = T_{raster}$). This ratio depends on the shape and extent of the ROIs, i.e. the geometry to be scanned. Alternatively, we can keep the SNR constant ($SNR_{ROI} = SNR_{raster}$) and this will result in increased measurement speed when using ROI scanning. As the ratio of T_{raster} and T_{ROIs} measurement times is equivalent to the ratio of measurement speeds of ROI and raster-scanning methods, it can be defined as the gain in the measurement speed:

$$v_{gain} := \frac{\mathrm{T}_{raster}}{\mathrm{T}_{ROIs}} = \frac{v_{ROIs}}{v_{raster}} \qquad \textbf{[Equation S79]}$$

Therefore we can express **Equation S78** in the following form:

$$\left(SNR_{gain}\right)^2 * v_{gain} = \frac{A}{n \cdot a} \qquad \textbf{[Equation S80]}$$

We can generalize our formula for N different ROIs:

$$\left(SNR_{gain}\right)^2 * v_{gain} = \frac{A}{\sum_{i=1}^{N} a_i} \quad \textbf{[Equation S81]}$$

where a_i is the area of ROI number *i*. Importantly, the SNR_{gain} is the same in all a_i regions, independent of size.

SNR and speed improvement for 3D AO scanning

As stated above, when we switch from 2D to 3D by using AO scanning, it can significantly increase the product of the SNR of the signal measured from an ROI in given time unit, as compared to classical raster scanning of the entire volume. Quantitatively, by extending **Equation S81** for imaging in 3D yields:

$$\left(SNR_{gain}\right)^2 * v_{gain} = \frac{V_{total}}{\sum_{i=1}^{N} V_i} \quad \textbf{[Equation S82]}$$

where V_{total} is the total scanned volume and V_i is the volume of ROI number *i*. Importantly, the SNR_{gain} is the same in all V_i regions, independent of size.

When considering measurement of *N* different regions with $pixel_{xi} \times pixel_{yi} \times pixel_{zi}$ resolution in a scanning cubature of $pixel_{xmax} \times pixel_{ymax} \times pixel_{zmax}$ **Equation S82** can be formulated as:

$$\left(SNR_{gain}\right)^2 * v_{gain} = \frac{pixel_{x_{max}} * pixel_{y_{max}} * pixel_{z_{max}}}{\sum_{i=1}^{N}\left(pixel_{x_i} * pixel_{y_i} * pixel_{z_i}\right)} \quad \textbf{[Equation S83]}$$

The ≈60 MHz maximal bandwidth of the AO deflectors (**Figure S2B**) can be addressed with 4 kHz accuracy, providing a maximal resolution of 15,000 pixels, equivalent to 33 nm spatial accuracy (discretization) for the ≈500 µm lateral field of view. However, the maximal useful pixel number is limited by the optical resolution of the system (**Figure S4**). Calculating it by using the average lateral resolution (0.8 µm) for the maximal field of view (500 µm) and considering the Nyquist law, the maximal number of useful pixels is about 500/0.8 *2 = 1250 pixels. For the z direction: 700/4.5 *2 = 311, since the average optical resolution along the z axis is 4.5 µm.

An improvement of over six orders of magnitude in the product of SNR and measurement speed gains

In this section we show examples for the calculation of improvement in the SNR and measurement speed when 3D DRIFT AO scanning is used. In a typical *in vivo* measurement, where we use the currently available maximal scanning volume (500 µm × 500 µm × 650 µm with 0.4 µm × 0.43 µm × 2.3 µm central resolution, **Figure S4G**) and using a value of about 0.8 µm × 0.8 µm × 4.5 µm for the volume averaged resolution (**Figure S4C-F**), the gain in the SNR and measurement speed can be calculated from **Equation S82** as:

$$\left(SNR_{gain}\right)^2 * v_{gain} = \frac{V_{total}}{\sum_{i=1}^{N} V_i} = \frac{500\ \mu\text{m} \times 500\ \mu\text{m} \times 650\ \mu\text{m}}{0.8\ \mu\text{m} \times L \times 4.5\ \mu\text{m} \times N_{3D}} = \frac{4.5*10^7}{N_{3D} \times L} \approx \frac{1.8*10^6}{N_{3D}} \quad \textbf{[Equation S84]}$$

where N_{3D} is the number of 3D lines generated and L is the average length of the 3D lines (for which we substituted 25 µm to simplify the formula as we typically used a series of 25 µm 3D lines to cover somata in neuronal network measurements). **Equation S84** shows a very large increase in measurement speed, about six orders of magnitude per number of 3D lines higher, when compared to raster scanning at the same SNR level.

We can calculate the following formula for random-access point scanning (where L is ≈0.8 µm) from **Equations S84 and S82**:

$$\left(SNR_{gain}\right)^2 * v_{gain} \approx \frac{5.64*10^7}{points} \quad \textbf{[Equation S85]}$$

where *points* means the number of PSF sized volume elements that form the object.

Quantitative example of calculations of SNR and measurement speed gains

More quantitatively, if we want, for example, to record 136 somata with chessboard scanning using 25 x 25 pixel frames for each soma (as shown in **Figures 4A-D**),

the maximal gain in SNR and measurement speed relative to raster scanning of the entire volume, from **Equation S84** is:

$$\left(SNR_{gain}\right)^2 * v_{gain} \approx 663 \qquad \textbf{[Equation S86]}$$

corresponding to a 663-fold higher measurement speed when the SNR level is kept constant. When the measurement speed is kept constant (v_{gain}=1) the SNR will increase by a factor:

$$\left(SNR_{gain}\right)\Big|_{v=const.} = 25.7 \quad \textbf{[Equation S87]}$$

due to the much higher integration time in each measured point.

In summary, 3D AO scanning can improve the SNR and measurement speed by several orders of magnitude because the relative ratio of the ROIs to the entire scanning volume is small in a typical measurement, therefore no measurement time is wasted scanning unnecessary regions.

The LOTOS approach with 3D DRIFT AO microscopy

The LOTOS approach states that faster scanning results harm the sample less (Chen et al., 2012). Earlier attempts which demonstrated the advantage of the LOTOS approach included line-scan rate of 100 kHz with 2D acousto-optical setup (Chen et al., 2012) or line-scan rate of 24 kHz using a resonant (mirror) scanner (12 kHz bidirectional; Varga et al., 2011). Our method, with its line-scan rate of 53 kHz, is in the previously validated frequency range.

Practically, the repetition rate of measurement is what counts, i.e. how regularly the excitation beam returns to the same point during scanning. At higher repetition rate we can keep the same SNR for the Ca2+ transients recorded while the cumulative laser intensity (integrated for a given pixel) can be decreased or, alternatively, we can get a higher SNR while keeping the same cumulative laser intensity. A lower cumulative laser intensity results in a less tissue damage: Tissue damage is inversely (and non-linearly) proportional to laser intensity (see for example: Ji et al., 2008). Chen et al. claimed to achieve 1 kHz frame repetition rates, while Varga et al. claimed 0.2 kHz. With our 3D DRIFT AO microscope we can achieve the 1 kHz measurement repetition rate during 3D recording of 5 neurons by using 10 x 50 pixel frames for each soma in chessboard scanning mode. Similarly, it is possible to measure 25 neurons with the lower 0.2 kHz repetition rate.

Besides being able to select multiple separate regions for scanning, and even in 3D, we were able to utilize smaller pixel numbers (thus proportionally larger repetition rates) because of the greater flexibility of the ROI selection in our 3D DRIFT AO setup. We can place small rectangular ROIs to match the dendrite in any 3D orientation, while Chen et al. and Varga et al. can only place rectangles in the focal plane, having the fast/long axis set by the mechanics of the microscope).

In summary, we can say that the spatial and temporal flexibility of 3D DRIFT AO microscopy not only extends the LOTOS approach to 3D but can also improve unitization by providing a higher and flexibly adjustable measurement repetition rate.

Supplemental discussion

It has been reported that for many cortical neurons, synaptic integration occurs not only at the axon initial segment but also within the apical and basal dendritic tree (Schiller et al., 2000, Magee and Johnston, 2005, Johnston and Narayanan, 2008, Larkum et al., 2009). Here, dendritic segments form non-linear computational subunits which also interact with each other, for example through local regenerative activities generated by non-linear voltage-gated ion channels (Polsky et al., 2004, Chiovini et al., 2014, Tran-Van-Minh et al., 2015). However, in many cases, the direct result of local dendritic computational events remains hidden in somatic recordings (Schiller et al., 2000, Larkum et al., 2009, Araya, 2014). To understand computation in neuronal networks we also need novel methods, such as our 3D scanning methods, for the simultaneous measurement of multiple spiny dendritic segments. The increased SNR and preserved high spatial and temporal resolution, allow, among other advantages, a better understanding of spike-timing-dependent plasticity and the underlying mechanisms during *in vivo* conditions (Bloodgood and Sabatini, 2007, Harvey et al., 2008, Sjostrom et al., 2008), dendritic computation between multiple spiny and aspiny dendritic segments (Losonczy and Magee, 2006, Chiovini et al., 2014), multisensory integration (Olcese et al., 2013). These 3D scanning methods may also provide the key to understanding synchronization processes mediated by neuronal circuitry locally and on a larger scale: these are thought to be important in the integrative functions of the nervous system (Womelsdorf et al., 2014) or in different diseases (Engel et al., 2013).

References:

Bloodgood BL, Sabatini BL (2007) Ca(2+) signaling in dendritic spines. Current opinion in neurobiology 17:345-351.

Chen TW, Wardill TJ, Sun Y, Pulver SR, Renninger SL, Baohan A, Schreiter ER, Kerr RA, Orger MB, Jayaraman V, Looger LL, Svoboda K, Kim DS (2013) Ultrasensitive fluorescent proteins for imaging neuronal activity. Nature 499:295-300.

Chen X, Leischner U, Rochefort NL, Nelken I, Konnerth A (2011) Functional mapping of single spines in cortical neurons in vivo. Nature 475:501-505.

Chen X, Leischner U, Varga Z, Jia H, Deca D, Rochefort NL, Konnerth A (2012) LOTOS-based two-photon calcium imaging of dendritic spines in vivo. Nature protocols 7:1818-1829.

Chiovini B, Turi GF, Katona G, Kaszas A, Erdelyi F, Szabo G, Monyer H, Csakanyi A, Vizi ES, Rozsa B (2010) Enhanced dendritic action potential backpropagation in parvalbumin-positive basket cells during sharp wave activity. Neurochemical research 35:2086-2095.

Chiovini B, Turi GF, Katona G, Kaszas A, Palfi D, Maak P, Szalay G, Szabo MF, Szabo G, Szadai Z, Kali S, Rozsa B (2014) Dendritic spikes induce ripples in parvalbumin interneurons during hippocampal sharp waves. Neuron 82:908-924.

Engel J, Jr., Thompson PM, Stern JM, Staba RJ, Bragin A, Mody I (2013) Connectomics and epilepsy. Current opinion in neurology 26:186-194.

Goldey GJ, Roumis DK, Glickfeld LL, Kerlin AM, Reid RC, Bonin V, Schafer DP, Andermann ML (2014) Removable cranial windows for long-term imaging in awake mice. Nature protocols 9:2515-2538.

Harvey CD, Yasuda R, Zhong H, Svoboda K (2008) The spread of Ras activity triggered by activation of a single dendritic spine. Science 321:136-140.

Holthoff K, Tsay D, Yuste R (2002) Calcium dynamics of spines depend on their dendritic location. Neuron 33:425-437.

Ji N, Magee JC, Betzig E (2008) High-speed, low-photodamage nonlinear imaging using passive pulse splitters. Nature methods 5:197-202.

Katona G, Kaszas A, Turi GF, Hajos N, Tamas G, Vizi ES, Rozsa B (2011) Roller Coaster Scanning reveals spontaneous triggering of dendritic spikes in CA1 interneurons. Proceedings of the National Academy of Sciences of the United States of America 108:2148-2153.

Katona G, Szalay G, Maak P, Kaszas A, Veress M, Hillier D, Chiovini B, Vizi ES, Roska B, Rozsa B (2012) Fast two-photon in vivo imaging with three-dimensional random-access scanning in large tissue volumes. Nature methods 9:201-208.

Konur S, Rabinowitz D, Fenstermaker VL, Yuste R (2003) Systematic regulation of spine sizes and densities in pyramidal neurons. Journal of neurobiology 56:95-112.

Losonczy A, Magee JC (2006) Integrative properties of radial oblique dendrites in hippocampal CA1 pyramidal neurons. Neuron 50:291-307.

Maák PJ, László; Barócsi, Attila; Richter, Péter (1999) Improved design method for acousto-optic light deflectors. Optics Communications 172:297-324.

Olcese U, Iurilli G, Medini P (2013) Cellular and synaptic architecture of multisensory integration in the mouse neocortex. Neuron 79:579-593.

Pi HJ, Hangya B, Kvitsiani D, Sanders JI, Huang ZJ, Kepecs A (2013) Cortical interneurons that specialize in disinhibitory control. Nature 503:521-524.

Rozsa B, Zelles T, Vizi ES, Lendvai B (2004) Distance-dependent scaling of calcium transients evoked by backpropagating spikes and synaptic activity in dendrites of hippocampal interneurons. The Journal of neuroscience : the official journal of the Society for Neuroscience 24:661-670.

Saleh BAE, Malvin CT (2001) Ray Optics: Wiley Online Library.

Sjostrom PJ, Rancz EA, Roth A, Hausser M (2008) Dendritic excitability and synaptic plasticity. Physiological reviews 88:769-840.

Varga Z, Jia H, Sakmann B, Konnerth A (2011) Dendritic coding of multiple sensory inputs in single cortical neurons in vivo. Proc Natl Acad Sci U S A 108:15420-15425.

Womelsdorf T, Valiante TA, Sahin NT, Miller KJ, Tiesinga P (2014) Dynamic circuit motifs underlying rhythmic gain control, gating and integration. Nature neuroscience 17:1031-1039.
