# Supplementary figures and images for: Fast 3D Imaging of Spine, Dendritic, and Neuronal Assemblies in Behaving Animals
Source: Neuron. 2016 Nov 23;92(4):723–38. doi: 10.1016/j.neuron.2016.10.002 (PMC5167293; doi:10.1016/j.neuron.2016.10.002)

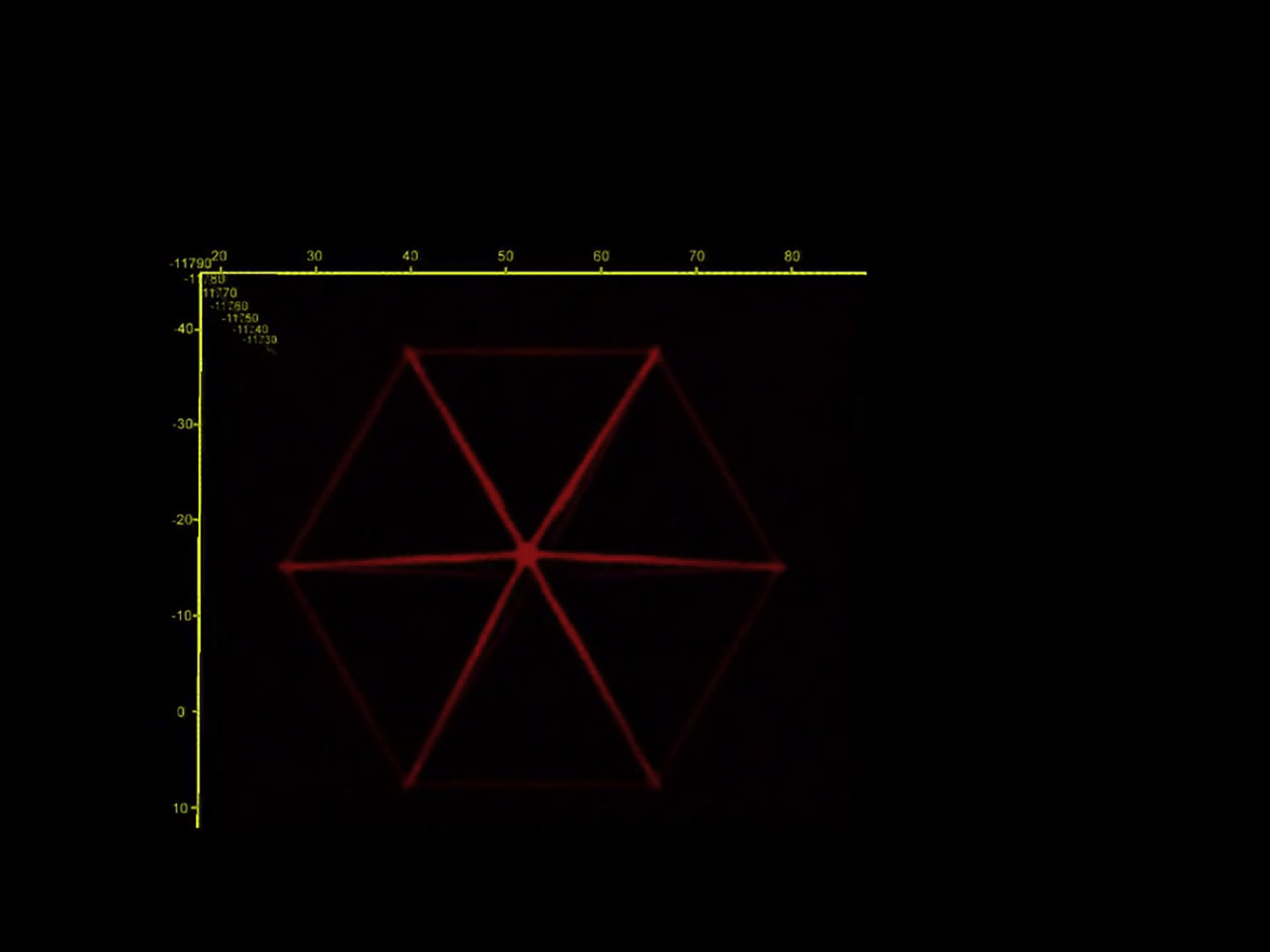

Supplement: Movie S1. Validation of 3D DRIFT AO Scanning by Bleaching a Polyhedron Shape in a Homogeneous Fluorescent Sample — Related to Figure 1. Resolution and point stability of the 3D DRIFT AO scanning method was validated by “burning” a polyhedron shape in a homogeneous fluorescent sample using photo bleaching. Each edge of the polyhedron was scanned by a 3D line that was generated within a single AO-switch time period (33 μs). The precision and reproducibility of the 3D scanning were verified by a z stack performed at a much lower intensity. The movie shows the inverted fluorescence intensity of the z stack. Note the stability of the 3D lines during scanning. [file mmc2.jpg]

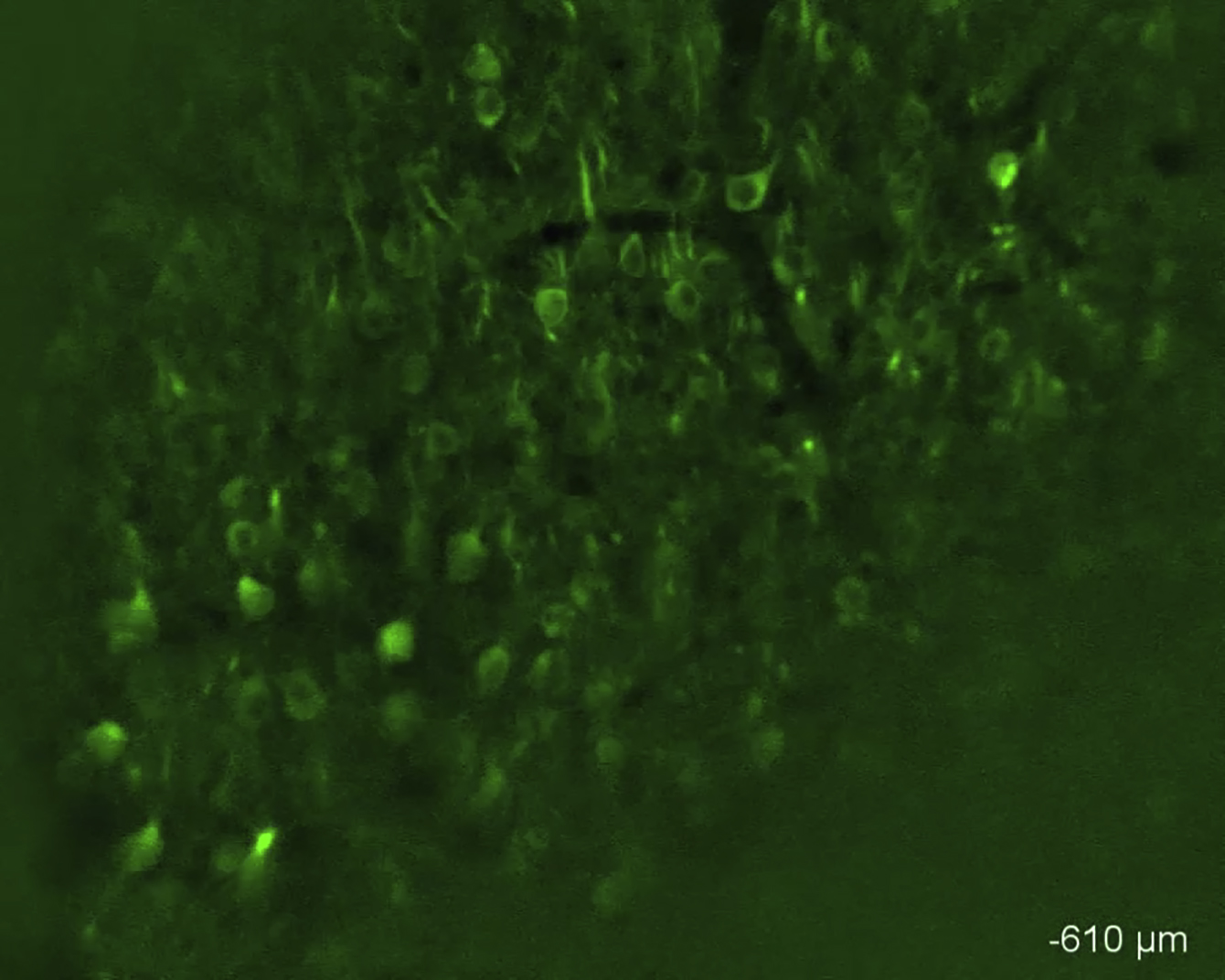

Supplement: Movie S2. Large AO 3D Scanning Volume Using GECIs — Related to Figure 1. z stack from 650 to 100 μm under the pia mater in the visual cortex of a Thy1-Cre mouse was taken using the 3D DRIFT AO microscope. For cre-dependent expression of the GCaMP6f Ca2+ sensor, we used an AAV vector injected into the V1 region. Raw fluorescence data were normalized for radially inhomogeneous illumination as previously described (Katona et al., 2012). [file mmc3.jpg]

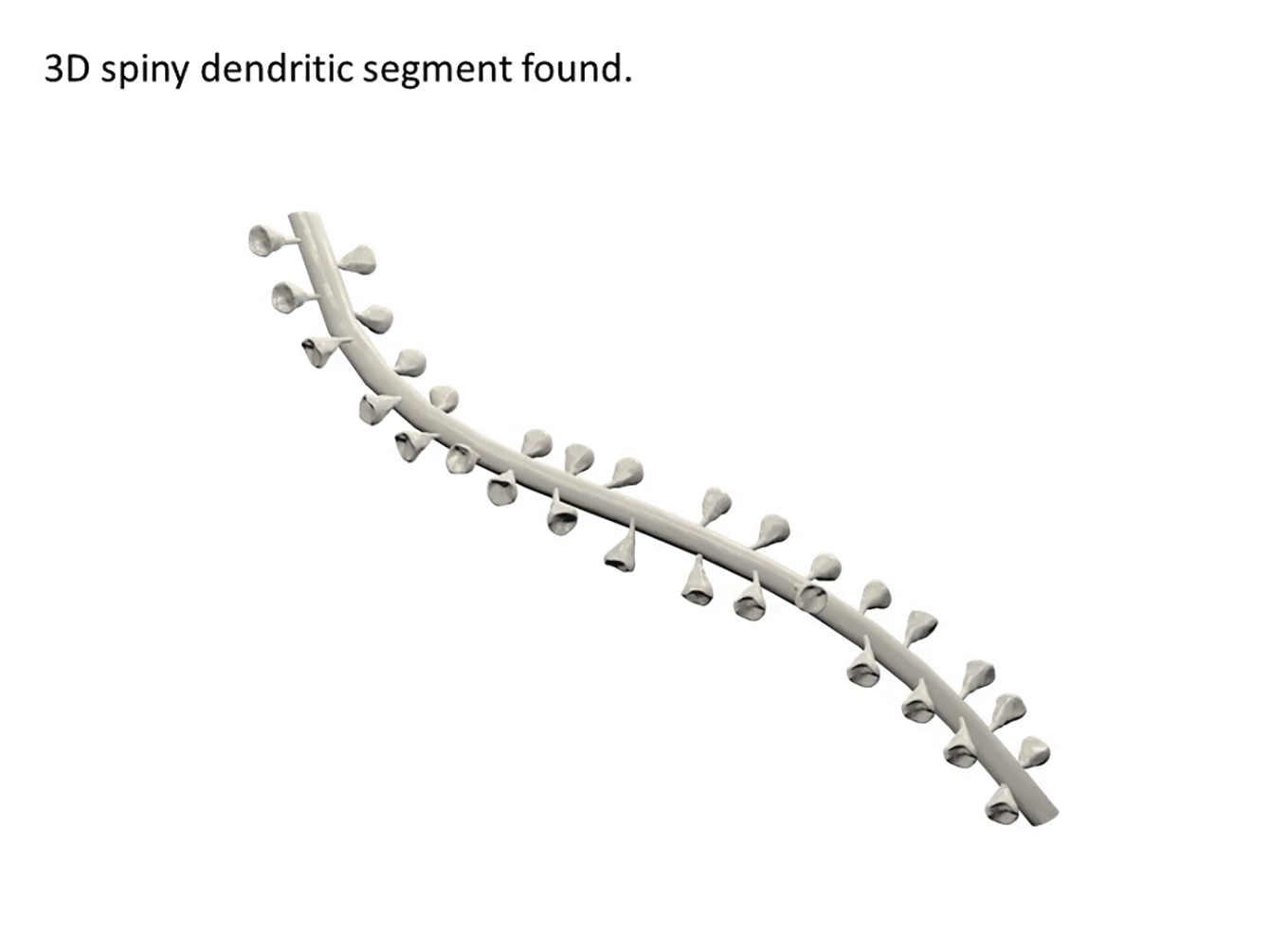

Supplement: Movie S3. Method of Selection of 3D Ribbons for Fast Scanning of Spiny Dendritic Segments — Related to Figure 1. The video demonstrates how guiding points are selected, how the 3D trajectory is fitted according to the guiding points, and, finally, how the plan of the ribbon is generated for 3D ribbon scanning. See also the “Selection of the 3D trajectory and the ribbons for 3D ribbon and snake scanning” section in the Supplemental Experimental Procedures. [file mmc4.jpg]

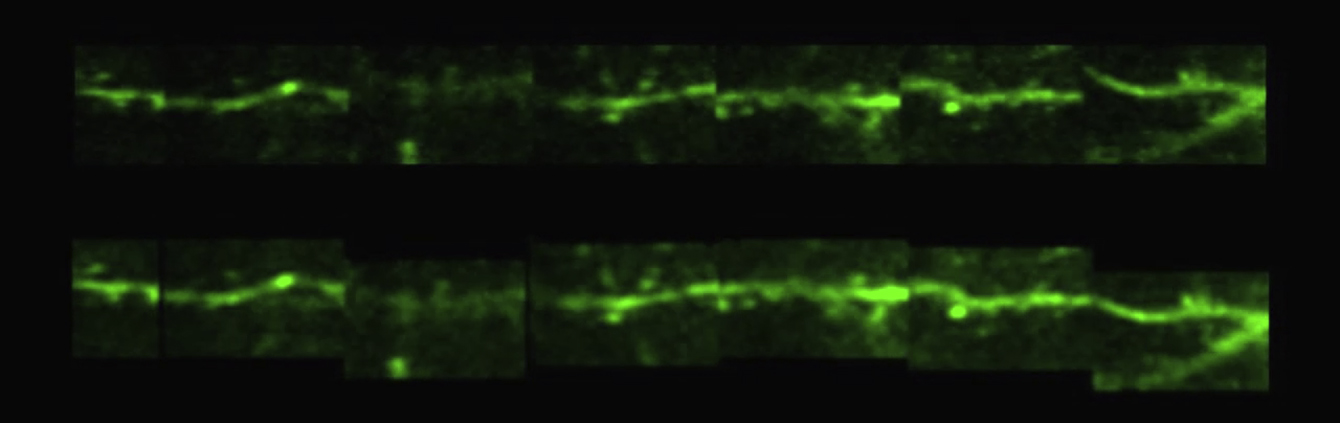

Supplement: Movie S4. 3D Ribbon Scanning of a Spiny Dendritic Segment — Related to Figure 1. A 140 μm GCaMP6-labeled spiny dendritic segment situated in a 60 μm range of z-scanning was imaged using 3D ribbon scanning. [file mmc5.jpg]

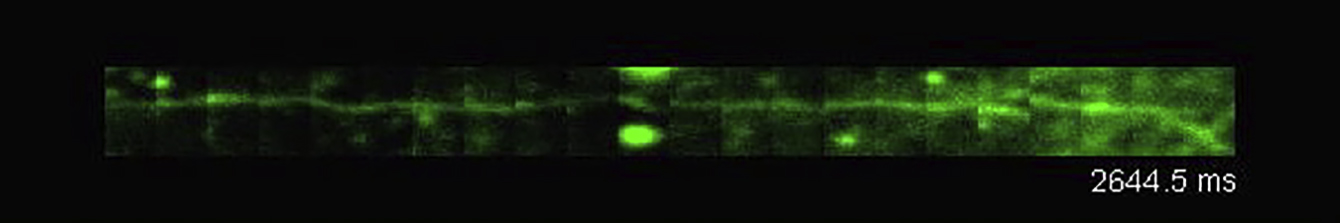

Supplement: Movie S5. 3D Ribbon Scanning of a Spiny Dendritic Segment during Visual Stimulation — Related to Figure 1. Similar example as Movie S4. A GCaMP6-labeled spiny dendritic segment from a different layer II/III neuron was imaged using 3D ribbon scanning. [file mmc6.jpg]

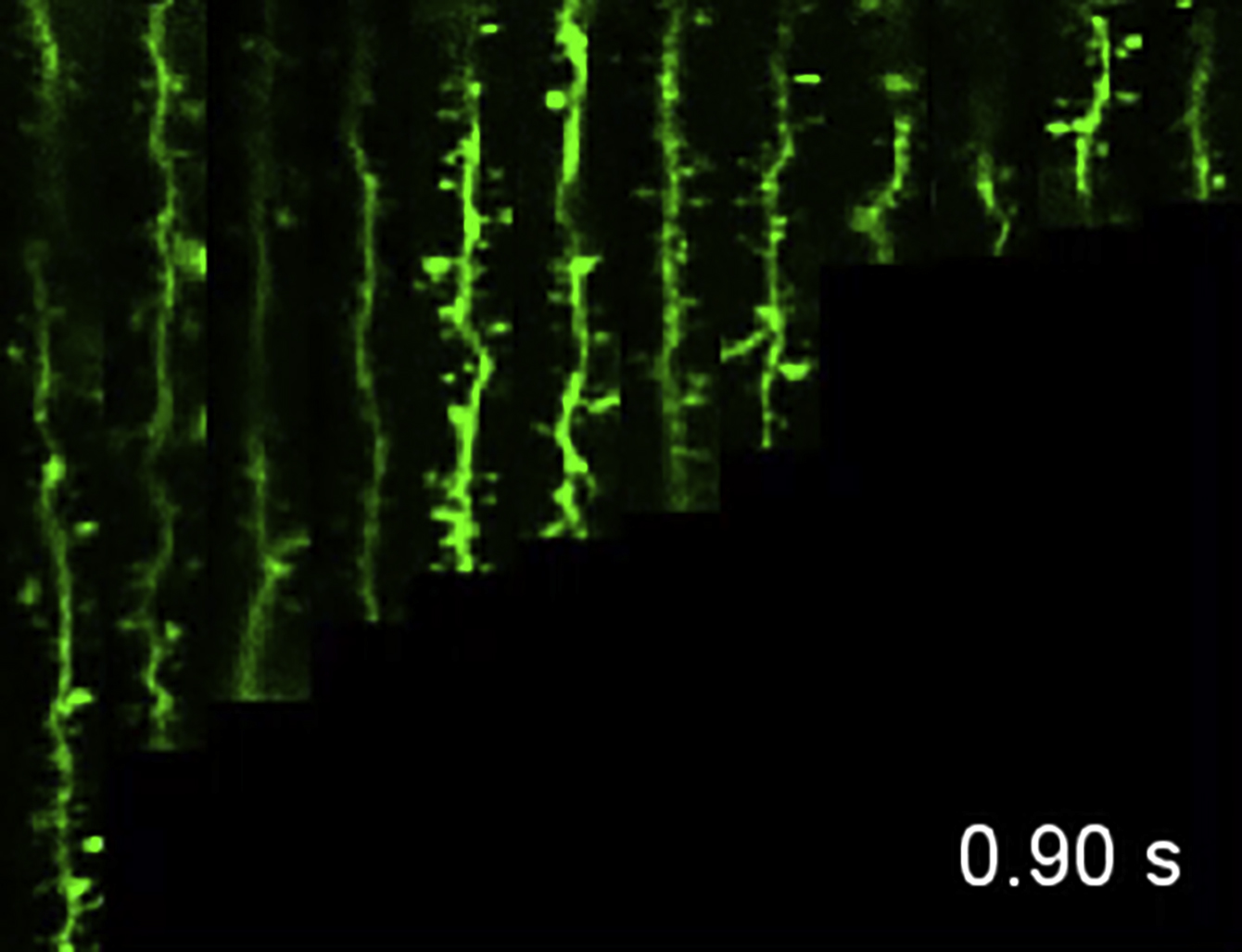

Supplement: Movie S6. Multi-3D Ribbon Scanning: Raw Data Recorded in Behaving Animal — Related to Figures 2 and 3. Twelve spiny dendritic segments of a GCaMP6-labeled layer II/III pyramidal cell located in V1 were simultaneously imaged using 3D ribbon scanning. Note the large and spatially inhomogeneous movement artifacts during recording of activity in an awake animal. [file mmc7.jpg]

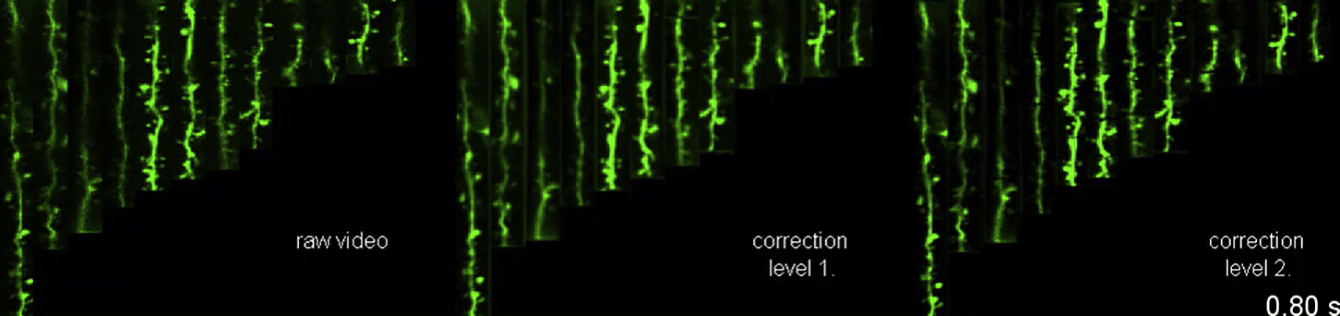

Supplement: Movie S7. Multi-3D Ribbon Scanning Movie Before and After the Correction of Motion Artifacts — Related to Figure 3. The first block is the same movie as Movie S5. In the second block of the movie, motion artifacts were eliminated by shifting back each frame of all regions by the local projection of the net displacement vector of the brain (see text for details). This correction was made with subpixel resolution. Note the small residual motion in the video. Finally, in the last block of the movie residual motion, artifacts were removed by repeating the cross-correlation-based displacement calculations and the consequent back shifts for each frame separately on all dendritic segments. [file mmc8.jpg]

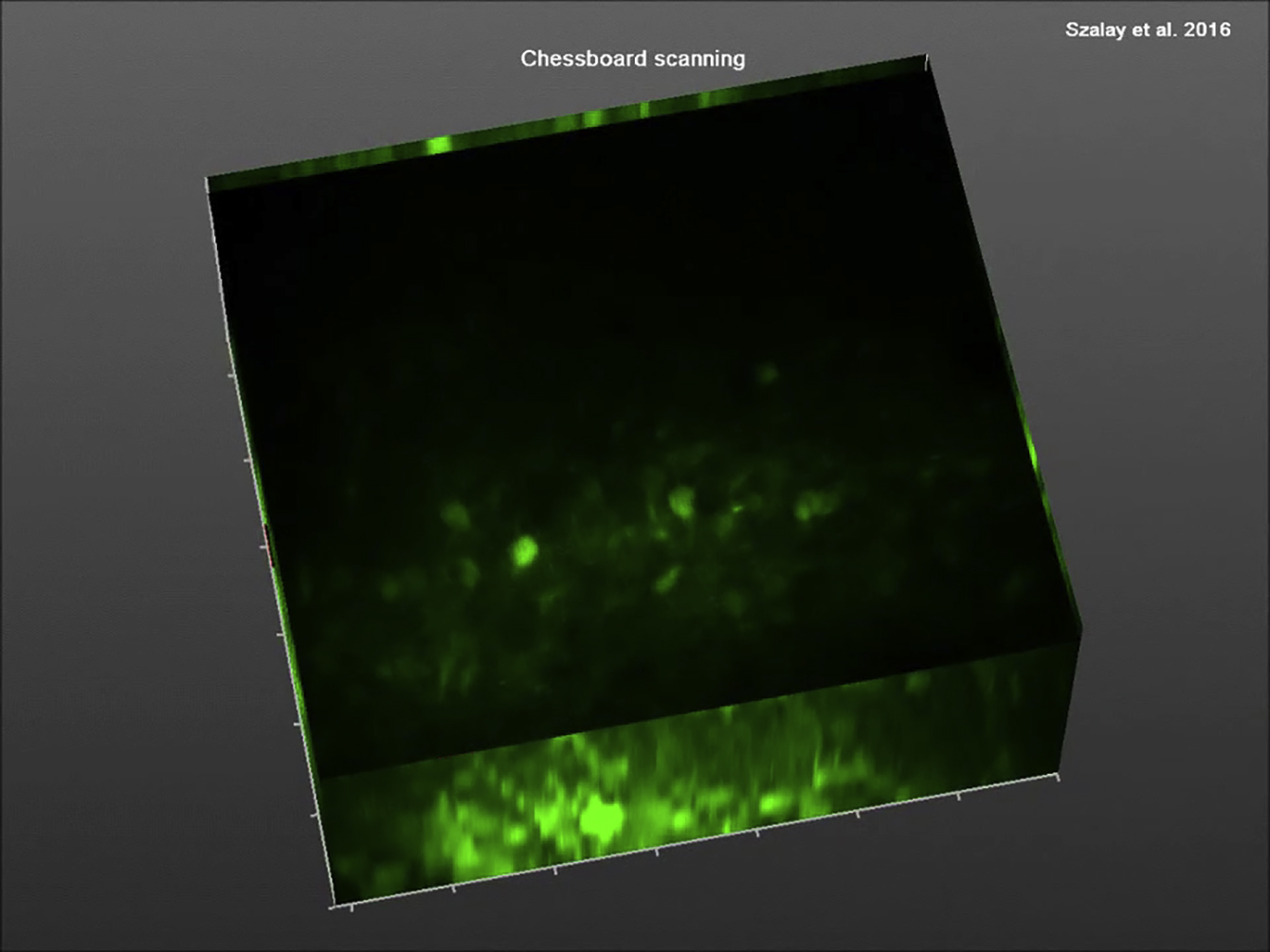

Supplement: Movie S8. Representative Example of Cell Selection and Image Acquisition during Chessboard Scanning in 3D — Related to Figures 2, 3, and 4. Populations of V1 neurons were labeled with GCaMP6f protein expressed by non-specific promoter. First, a z stack was obtained using the 3D AO microscope. Second, ROIs, here small squares centered on neuronal somata, were selected. Finally, the measured ROIs were arranged into the form of a chessboard. In this way, 3D activity was visualized during data acquisition and then stored as a 2D movie. [file mmc9.jpg]

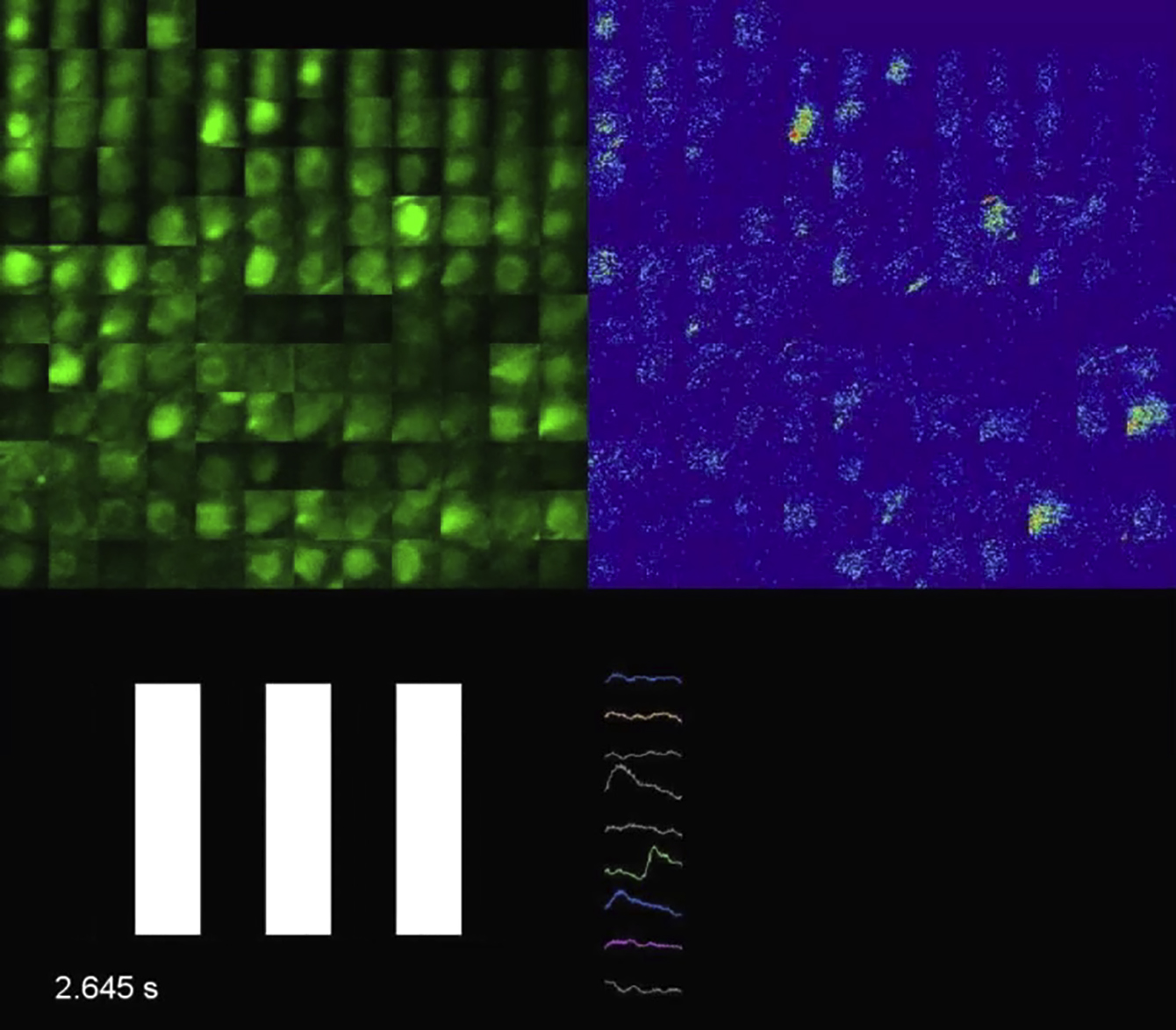

Supplement: Movie S9. Three-Dimensional Recording Neuronal Networks in V1 with Chessboard Scanning during Visual Stimulation — Related to Figure 4. The same populations of V1 neurons as in Movie S8. Activity was recorded in awake, head-restrained animals during visual stimulation. The upper left corner shows the video of raw fluorescence data arranged in chessboard form. The upper right corner shows the motion-corrected video converted to relative fluorescence changes (ΔF/F). In the lower left corner, the actual visual stimulation is presented, while in the lower right corner, the transients of nine exemplified neurons are shown during the actual visual stimulation. [file mmc10.jpg]

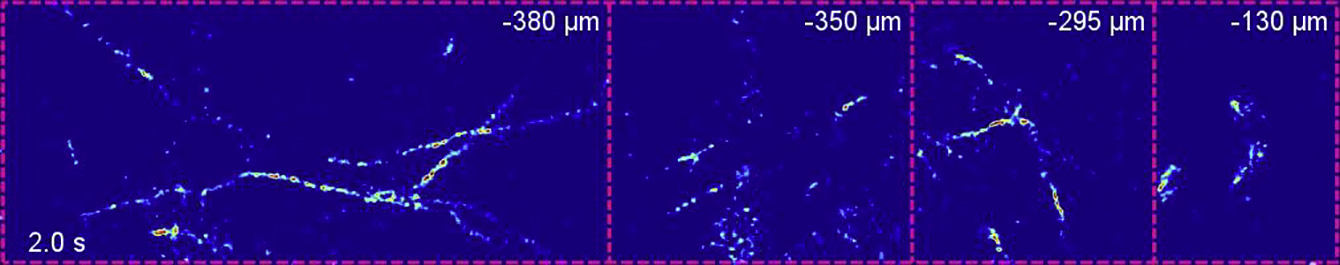

Supplement: Movie S10. Multi-Layer, Multi-Frame Imaging of Neuropil Activity with Over 100 Hz Temporal Resolution — Related to Figure 5. V1 neurons were sparsely labeled using the GCaMP6f sensor and measured simultaneously in four different z planes (z1 = −380 μm, z2 = −350 μm, z3 = −295 μm, z4 = −130 μm) with 101 Hz temporal resolution during visual stimulation with moving gratings. Movies were converted to relative fluorescence changes (ΔF/F) and aligned next to each other. [file mmc11.jpg]

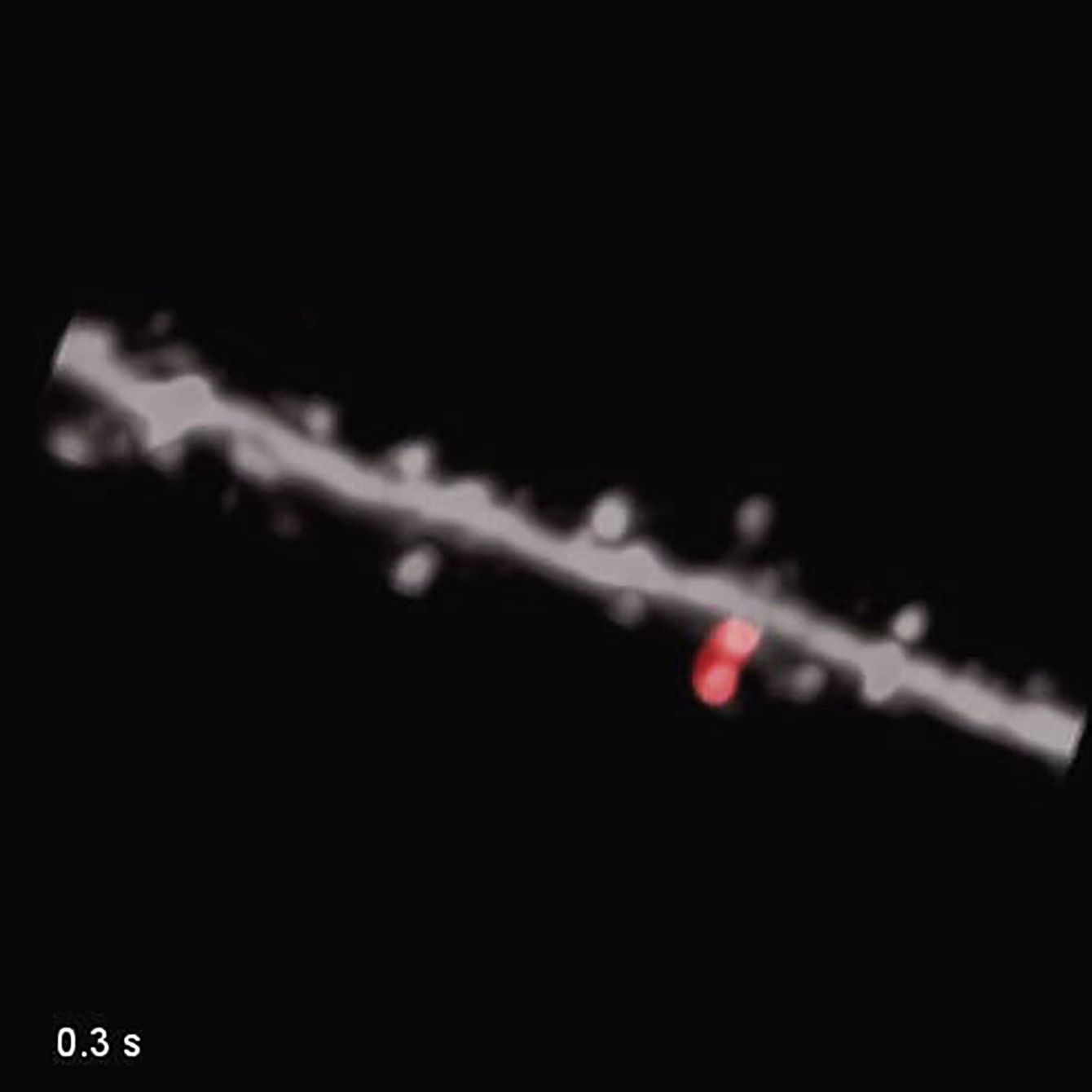

Supplement: Movie S11. Snake Scanning of a Spiny Dendritic Segment in the Moving Brain of a Behaving Mouse — Related to Figure 6. V1 neurons were sparsely labeled using the GCaMP6f sensor. A spiny dendritic segment was selected for volume imaging using snake scanning at 10 Hz. Motion artifacts were removed and data are shown as relative fluorescence changes (ΔF/F) overlaid on the averaged baseline fluorescence. [file mmc12.jpg]

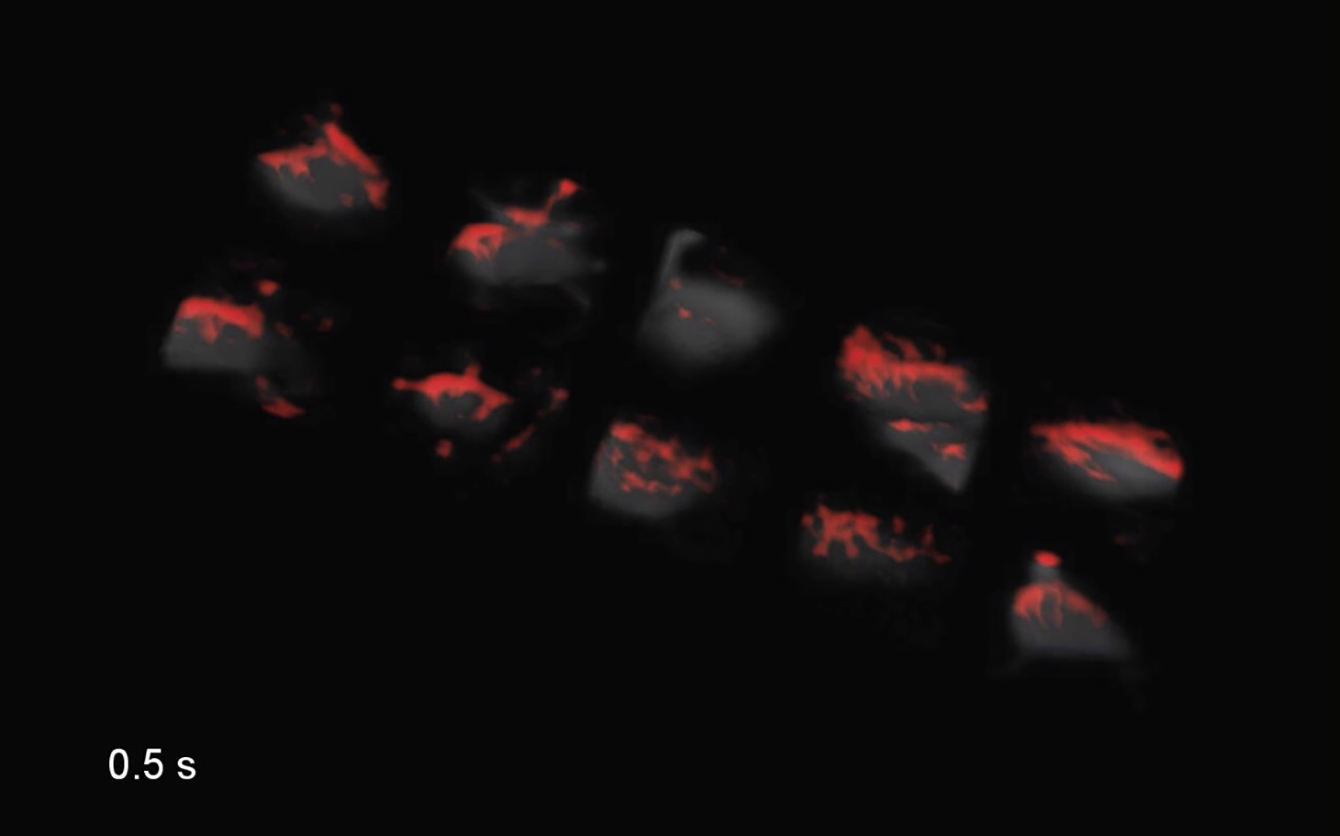

Supplement: Movie S12. Multi-Cube Imaging of Neuronal Activity — Related to Figure 6. V1 neurons were sparsely labeled using the GCaMP6f sensor. Ten labeled neurons were centered in cubes. Volume imaging was performed simultaneously in the ten cubes with 10 Hz temporal resolution. Finally, motion artifacts were removed and data are shown as relative fluorescence changes (ΔF/F). Note the fine Ca2+ dynamic in the somatic and proximal dendritic segments. [file mmc13.jpg]

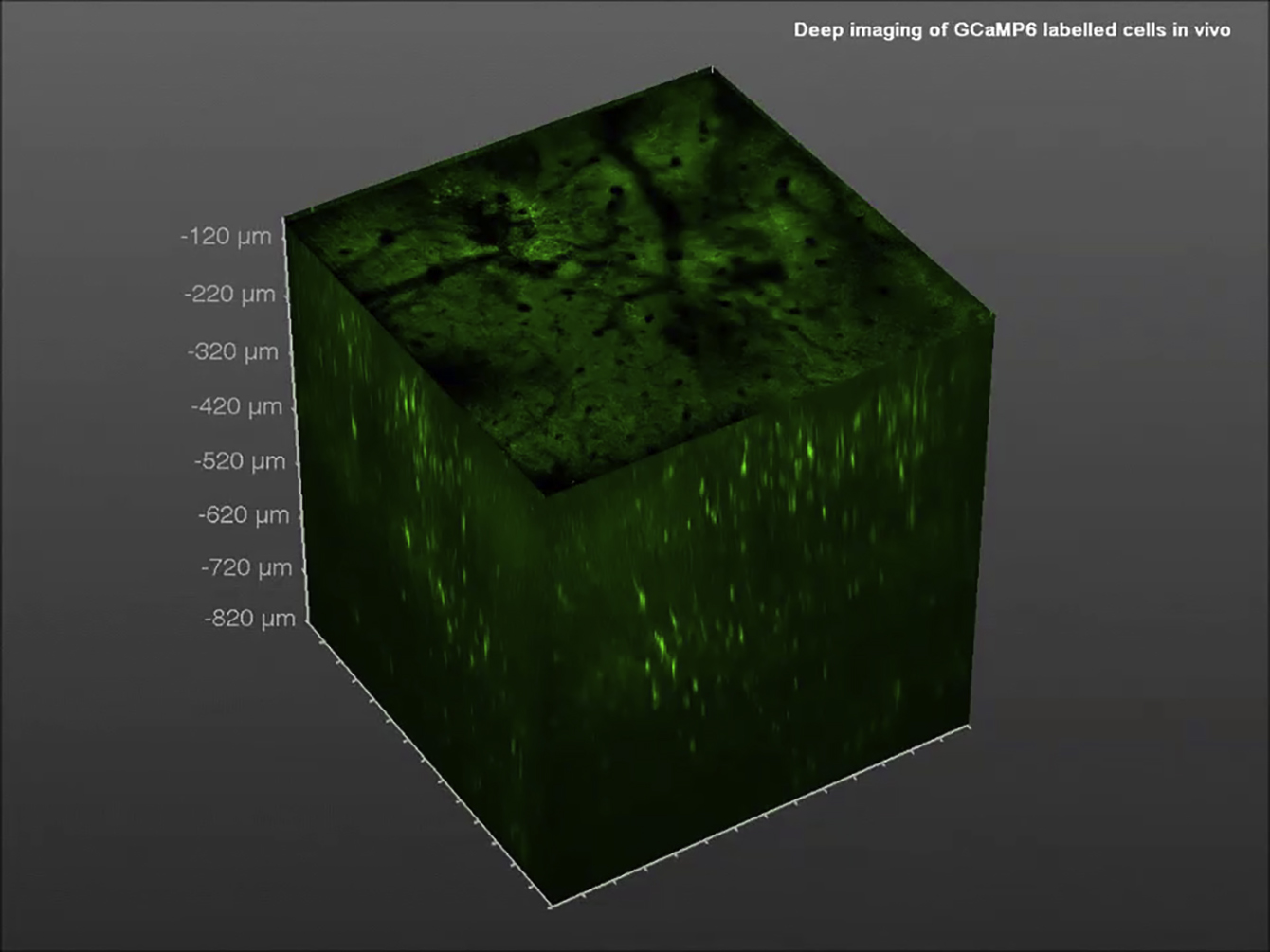

Supplement: Movie S13. z Stack of a GCaMP6f-Labeled Neuronal Population — Related to Experimental Procedures. z stack from 0 to 820 μm under the pia mater in the visual cortex of a Thy1-Cre mouse was taken using 2D galvo scanning. Detectors were fixed to the objective arm (traveling detector system) to minimize the detection pathway and maximize detection of backscattered fluorescent light. For cre-dependent expression of the GCaMP6f Ca2+ sensor, we used an AAV vector injected into the V1 region. See Supplemental Experimental Procedures. [file mmc14.jpg]
